# Reactions of 3-aryl-1-(trifluoromethyl)prop-2-yn-1-iminium salts with 1,3-dienes and styrenes

**DOI:** 10.3762/bjoc.16.173

**Published:** 2020-08-24

**Authors:** Thomas Schneider, Michael Keim, Bianca Seitz, Gerhard Maas

**Affiliations:** 1 Institute of Organic Chemistry I, Ulm University, Albert-Einstein-Allee 11, D-89081 Ulm, Germany

**Keywords:** alkynes, aromatic substitution, cyclization, cycloaddition, iminium salts

## Abstract

3-Aryl-1-(trifluoromethyl)prop-2-yn-1-iminium triflate salts represent a novel, highly reactive class of acetylenic iminium salts. Herein we present several reactions which are based on the electron-poor acetylenic bond and on the high electrophilicity of the CF_3_-substituted iminium group. These salts were found to be highly reactive dienophiles in Diels–Alder reactions with cyclopentadiene, 2,3-dimethylbutadiene and even anthracene. At higher temperature, the cycloadducts undergo an intramolecular S_E_(Ar) reaction leading to condensed carbocycles incorporating a 1-(trifluoromethyl)-1-(dimethylamine)indene ring system. With styrenes and some substituted styrenes, cascade reactions take place, which likely include cyclobutene and several cationic intermediates and mainly yield 2-(1-phenylvinyl)indenes. In a similar reaction cascade, a fulvene derivative was obtained with 1,4-diphenylbutadiene as the substrate.

## Introduction

In recent years, a trifluoromethyl substituent has been included quite often in the design of compounds which were developed for applications in various fields, such as biological and medicinal chemistry, agrochemicals, transition metal ligands, and materials science [1–5]. The particular characteristics of the C‒F bond [6–7] are the basis for the electronic and steric properties of the CF_3_ group, such as a strong electron-withdrawing (−I) effect, the accumulation of negative charge density in a relatively small volume and the low polarizability of the fluoro atoms. These and other substituent effects can modulate the conformational, physicochemical and electronic properties of a molecule.

Two major strategies exist to introduce a CF_3_ group into a target molecule: formation of a carbon‒ or heteroatom‒CF_3_ bond [8–9] and the use of preformed CF_3_-substituted building blocks. During our studies on acetylenic iminium salts, among the numerous CF_3_-substituted building blocks α-(trifluoromethyl)iminium salts RCH(CF_3_)=N^+^R’_2_ X^−^ attracted our attention, because a) the CF_3_ group should significantly increase the electrophilicity of the iminium functional group and b) these salts are known to react with a variety of nucleophiles to afford products containing a C(CF_3_)NR_2_ moiety. In particular C(CF_3_)NHR and C(CF_3_)NH_2_ groups are of interest as pharmacophores in the design of bioactive compounds [10–13].

Simple α-(trifluoromethyl)iminium salts (RCH(CF_3_)=N^+^Me_2_ X^−^, R= H, CF_3_) with weakly nucleophilic or non-nucleophilic anions can be isolated [14–15], but in organic synthesis, they are most often generated in situ from suitable precursors and are directly exposed to diverse nucleophiles. Such transformations have been achieved using CH_2_(CF_3_)NR_2_ as the iminium ion precursors [15], trifluoroacetaldehyde hemiaminals [16–18], *N*,*O*-acetals [19] and *N*,*N*-acetals [20], 2-(trifluoromethyl)-1,3-oxazolidines [21], and *N*-(*tert*-butylsulfinyl)trifluoroacetaldimine [11,22]. Other synthetic approaches to α-CF_3_-substituted amines include nucleophilic trifluoromethylation strategies [17], such as the reaction of trifluoroacetaldehyde hemiaminals with enolizable carbonyl compounds in the presence of a strong base [23], the reaction of aldiminium salts with (trifluoromethyl)trimethylsilane/Lewis base [24], and the preparation of secondary α-(trifluoromethyl)propargylamines from imines CF_3_CH=NR and lithium acetylides [25]. By a photoredox-catalytic process, primary α-(trifluoromethyl)-α-(4-pyridyl)benzylamines were obtained from α-(trifluoromethyl)-benzaldoximes and 4-cyanopyridine [26].

We have recently introduced a new class of acetylenic iminium salts, namely 1-(trifluoromethyl)prop-2-yn-1-iminium triflates R‒C≡C‒C(CF_3_)=N^+^Me_2_ TfO^−^ [27]. As a first synthetic application, we have reported the phospha-Michael addition providing 3-(triphenylphosphonio)-1-(trifluoromethyl)-1-(dimethylamino)allenes, which were subsequently transformed into α-(trifluoromethyl)pyrroles. In the present paper, we consider the reactivity of the electrophilic (“electron-poor”) acetylenic bond toward 1,3-dienes, and show how the expected [4 + 2] or [2 + 2] cycloaddition products can enter subsequent cascade reactions toward carbocycles which incorporate a C(CF_3_)NMe_2_ structural unit.

## Results and Discussion

**Scheme 1:**
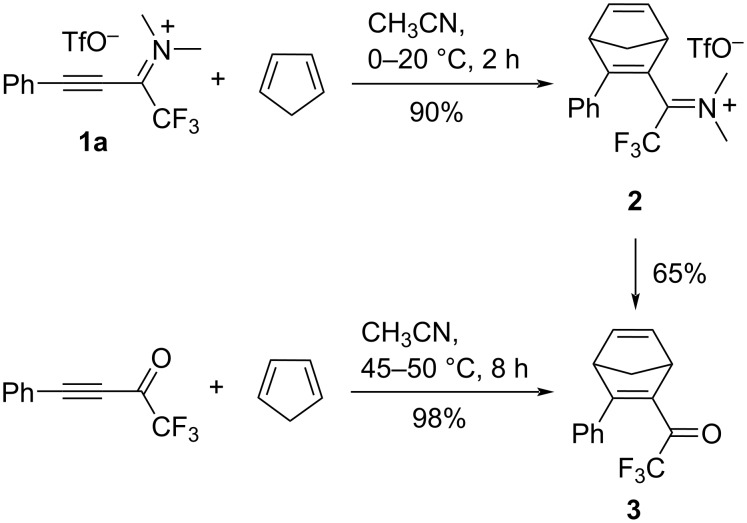
Diels–Alder reaction of propyn-1-iminium salt **1a** compared with the reported [29] reaction of 4-phenyl-1,1,1-trifluorobut-3-yn-2-one.

The Diels–Alder reaction of 1-CF_3_-substituted propyn-1-iminium salt **1a** with cyclopentadiene was carried out in order to assess the dienophilic reactivity of the cation. High conversion into cycloaddition product **2** was observed already within one hour at 0 °C. Because of its high hydrolytic lability, adduct **2** was not isolated but directly converted into the norbornadienyl trifluoromethyl ketone **3** (Scheme 1). The smooth [4 + 2] cycloaddition of **1a** as compared to comparably harsh thermal conditions of other propyne ketiminium salts with an internal acetylenic bond reveals the activating influence of the CF_3_ group, which has both an electronic (electron-withdrawing) and steric (e.g., CF_3_ vs a phenyl substituent [28]) component. Moreover, a comparison with the thermal conditions of the Diels–Alder reaction of 4-phenyl-1,1,1-trifluorobut-3-yn-2-one and cyclopentadiene [29] confirms the expected accelerating effect of the iminium activation.

**Scheme 2:**
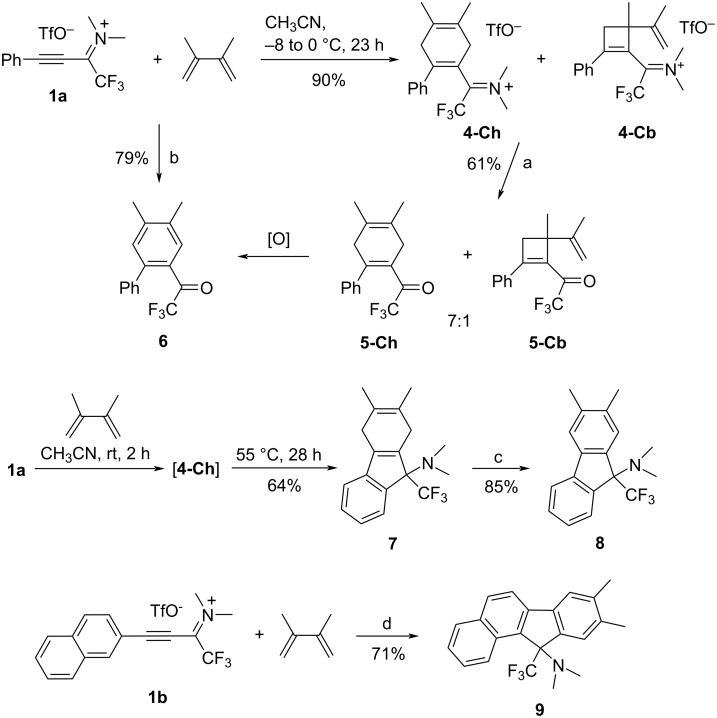
Sequential Diels–Alder/intramolecular S_E_(Ar) reaction of propyn-1-iminium triflates **1a**,**b**. Conditions: a) CH_3_CN, aqueous K_2_CO_3_, 15 min; b) 1. **1a** + 2,3-dimethylbutadiene, dry CH_2_Cl_2_, 0 °C, 30 min, then rt, 2 h; 2. *o*-chloranil, CH_2_Cl_2_, rt, 20 h; 3. K_2_CO_3_, CH_3_CN/H_2_O, rt, 2 h; c) *o*-chloranil, dry CH_2_Cl_2_, rt, 22 h, then K_2_CO_3_, H_2_O; d) 1. dry CH_3_CN, rt, 1 h; 2. 50 °C, 22 h; 3. *o*-chloranil, CH_2_Cl_2_, rt, 12 h.

The Diels–Alder reaction of alkyne **1a** with 2,3-dimethylbutadiene also occurred under very mild conditions and yielded the iminium-substituted 1,4-cyclohexadiene **4-Ch** (Scheme 2), which, due to its high sensitivity toward moisture, was not purified but was further converted in two steps into cyclohexadienyl ketone **5-Ch** by intentional hydrolysis followed by dehydrogenating aromatization leading to biphenyl-2-yl trifluoromethyl ketone **6**. The latter product was more effectively prepared in a one-pot cycloaddition/hydrolysis/aromatization sequence. ^1^H NMR spectra of unpurified 1,4-cyclohexadien-1-iminium salt **4-Ch** and 1,4-cyclohexadien-1-yl ketone **5-Ch** indicated the presence of a minor byproduct. In the case of **5-Ch**, obtained as an oil, the two components could not be separated by column chromatography; however, the ^1^H NMR spectrum suggested the cyclobutene structure **5-Cb** for the byproduct. Thus, signals of the two methyl groups (δ 1.63, 1.78 ppm) and two terminal olefinic protons (δ 4.88 ppm, m) are observed, and the ring-CH_2_ protons appear as an AB spin system (δ 2.75 and 2.92 ppm, ^2^*J* = 15.2 Hz). Obviously, **4-Cb** and **5-Cb** result from a formal [2 + 2] cycloaddition of **1a** and 2,3-dimethylbutadiene (DMBD), the regioselectivity of which is as expected for a highly asynchronous transition state with effective stabilization of the positive charge or a two-step ionic process (vide infra). The high electrophilic power of the 1-CF_3_-substituted propyn-1-iminium ion presumably renders an ionic [2 + 2] cycloaddition pathway competitive with the Diels–Alder reaction. The few reported examples of cyclobutene formation from alkynes and unactivated 1,3-dienes include the sensitized photocycloaddition of phenylacetylene and DMBD [30] and the gold(I)-catalyzed reaction of phenylacetylene and DMBD or isoprene [31]. On the other hand, the 1,1-diphenylpropargyl cation was found to react with 2,4-dimethyl-1,3-pentadiene to afford a product derived from an initial [4 + 2] cycloaddition [32].

When the Diels–Alder reaction of **1a** with DMBD was carried out at room temperature instead of 0 °C, ^19^F NMR monitoring of the reaction’s progress indicated the appearance of a second product beside the 1,4-cyclohexadien-1-iminium salt **4-Ch**. Further investigations revealed that the new product was the dihydrofluorene **7**, resulting from **4-Ch** by an intramolecular electrophilic aromatic substitution with the reactive (trifluoromethyl)iminium group as the electrophile (Scheme 2). The thermal conversion of **4-Ch** into **7** was optimized and finally allowed the preparation of the latter from **1a** in a one-pot, two-step, temperature-dependent Diels–Alder reaction/intramolecular S_E_(Ar) reaction sequence in good yield. Dehydrogenation of **7** with chloranil then provided 9-(dimethylamino)-9-(trifluoromethyl)fluorene **8**.

In an analogous reaction sequence, 11*H*-benzo[*a*]fluorene derivative **9** was obtained from 3-(2-naphthyl)propyn-1-iminium salt **1b** and 2,3-dimethylbutadiene (Scheme 2) in a one-pot three-step sequence. On the other hand, the successful thermal iminium-ion induced S_E_(Ar) reactions shown in Scheme 2 were not applicable to the iminium-substituted norbornadiene **2**, which suffered undefined polymerization on moderate heating in various solvents.

In contrast to so far unknown 9‑amino-9-(trifluoromethyl)-9*H*-fluorenes, compounds containing a 9-(trifluoromethyl)-9*H*-fluoren-9‑ol partial structure can be found in the patent literature [33–35], where they have been claimed for their pyruvate dehydrogenase kinase (PDHK) inhibitory activity.

**Scheme 3:**
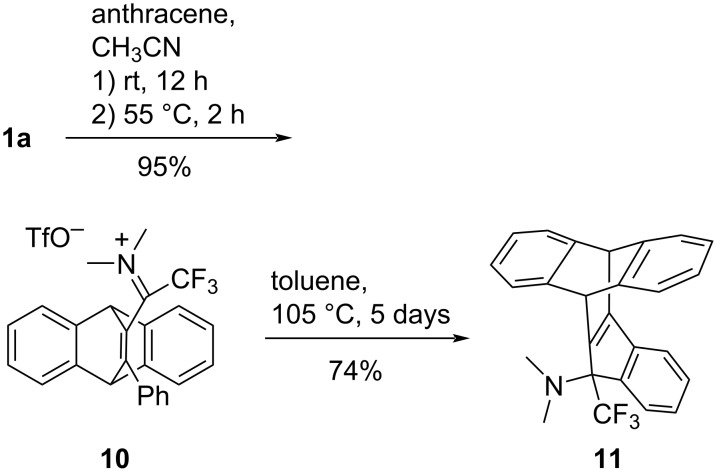
Diels–Alder reaction of **1a** and anthracene followed by an intramolecular S_E_(Ar) reaction.

**Figure 1:**
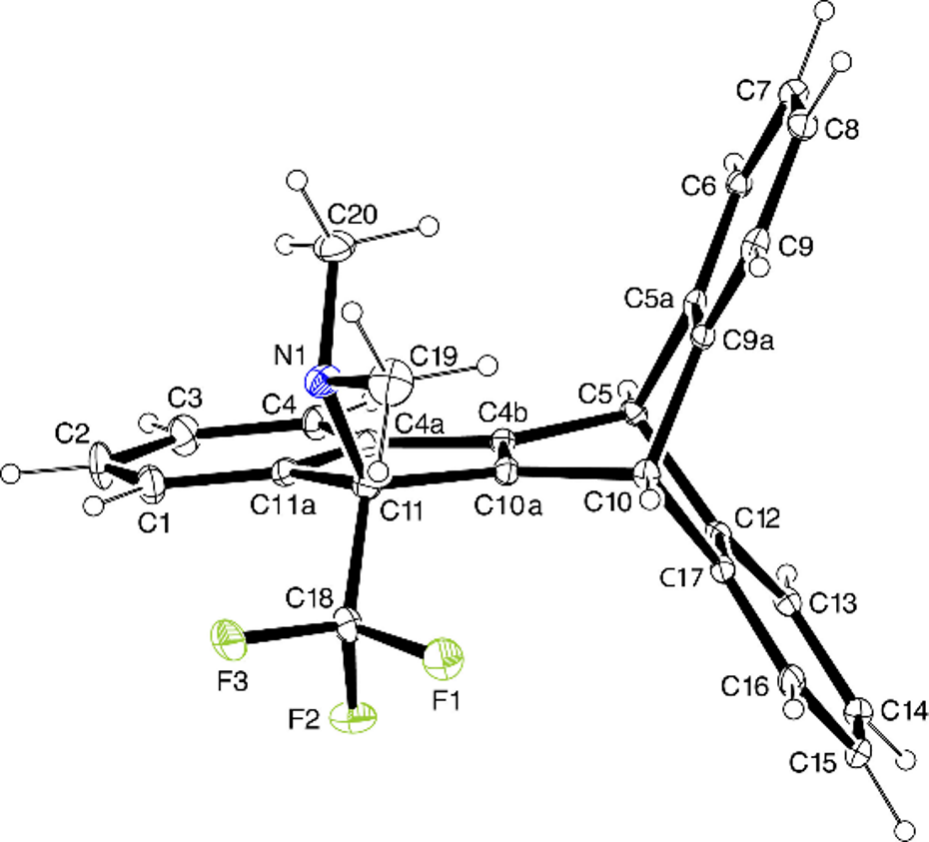
Solid-state molecular structure of **11** (ORTEP plot).

The remarkable dienophilic reactivity of 1-CF_3_-substituted propyn-1-iminium salts is also exemplified by the Diels–Alder reaction of **1a** with anthracene (Scheme 3). After 12 h at room temperature, a 91% conversion was observed, and subsequent moderate heating gave the cycloaddition product **10** in 95% yield. The subsequent iminium-induced electrophilic cyclization required extended heating in refluxing toluene and finally furnished the neutral polycycle **11** in good yield. The paddlewheel-shaped structure of **11** was established by an XRD structure determination and is shown in Figure 1.

The reactivity of **1a** in the Diels–Alder reaction with anthracene may be compared with that of other dienophiles. Thus, other propyn-1-iminium salts with an internal C,C triple bond and not containing a CF_3_ substituent (toluene, 120 °C, several hours [28]), DMAD (no solvent, 170–180 °C, 1 h [36]) and hexafluoro-2-butyne (200 °C, 2 h [37]) react only under harsher conditions, whereas terminal propyn-1-iminium salts (CH_2_Cl_2_, rt, 2‒4 h [38]) and tetracyanoethylene (rt, 12 h [39]) were found to react equally well or even faster than **1a**. Although these comparisons are only qualitative, they suggest that 1-CF_3_-substituted propyn-1-iminium salts have a high electrophilicity power and therefore, are candidates for polar Diels–Alder reactions [40–41].

The thermal cyclization of Diels–Alder adducts as shown in Scheme 2 and Scheme 3 appear to be the first intramolecular S_E_(Ar) reactions of an α-(trifluoromethyl)iminium functional group. Analogous intermolecular reactions of CF_3_-substituted iminium groups with electron-rich (hetero)aromatics are known [18,24]. Furthermore, 1-(trifluoromethyl)indenes have recently been generated by cationic cyclization of β-aryl trifluoromethyl enones under superacid conditions [42–44].

Styrenes are also known to behave as dienes in [4 + 2] cycloaddition reactions [45]. Thus, while styrene and maleic anhydride react only at elevated temperatures, with the more electrophilic (methoxycarbonyl)maleic anhydride two 2:1 adducts are already formed at room temperature: one by two consecutive Diels–Alder reactions, the other one by a Diels–Alder/ene reaction sequence [46]. Taking into account the presumably high electrophilic character of the 1-(trifluoromethyl)propyn-1-iminium ion, for its reaction with styrenes we could not exclude a priori an initial electrophilic addition at the olefinic bond (with formation of a benzyl cation intermediate).

The reaction of propyn-1-iminium salt **1a** with styrene in acetonitrile was considered first and was monitored by ^19^F NMR spectroscopy. Whereas no reaction appeared to occur at room temperature, a slow transformation into two fluorine-containing products was observed at 70 °C, which after neutralization and work-up were identified by their NMR and analytical data as 2-(1-phenylvinyl)indene **12a** and a small amount of benzo[*a*]fluorene **13a** (δ^F^ = −69.74 and −69.20 ppm, respectively).

**Scheme 4:**
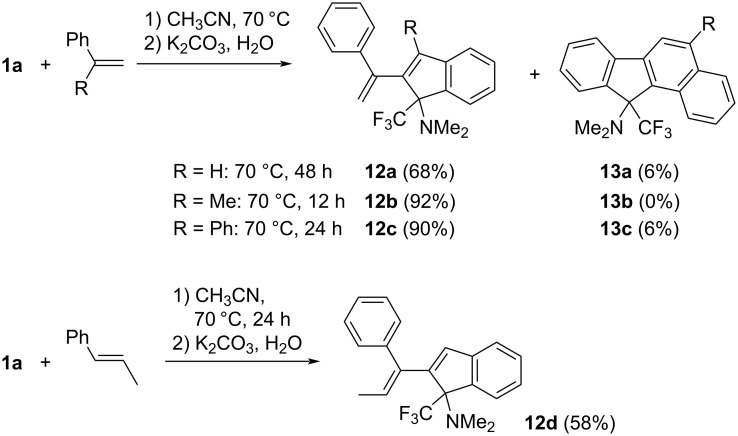
Reactions of propyn-1-iminium salt **1a** with styrenes.

**Figure 2:**
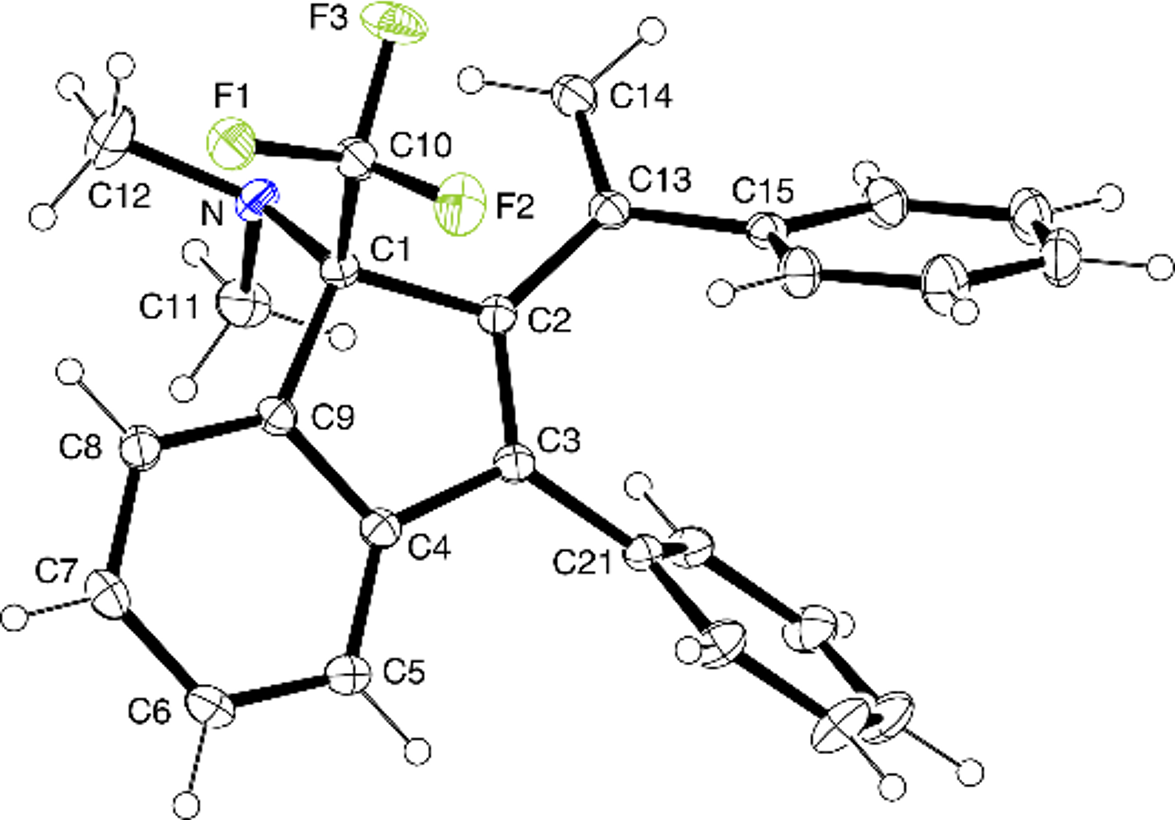
Solid-state molecular structure of **12c** (ORTEP plot).

**Figure 3:**
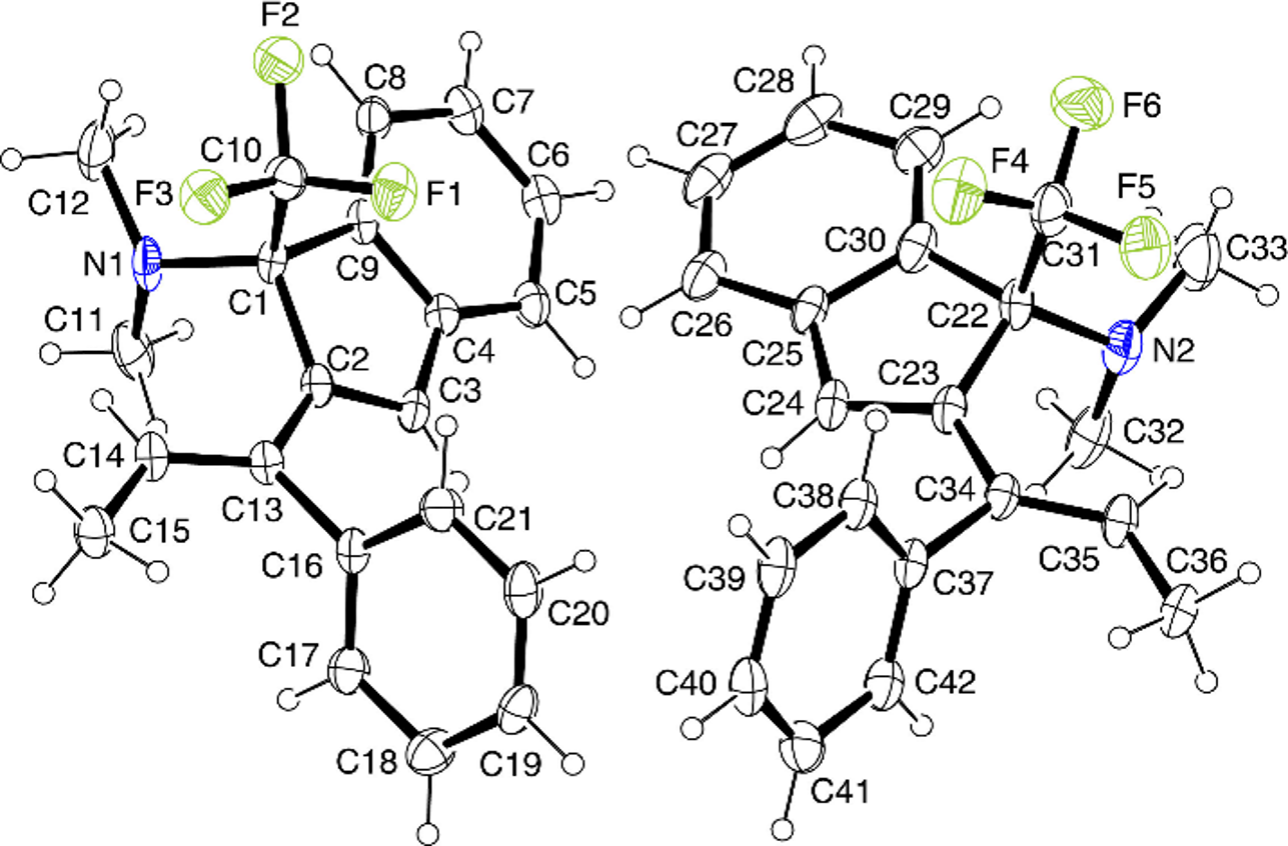
Solid-state molecular structure of **12d** (ORTEP plot). Both the *R* and the *S* enantiomer are present in the acentric unit cell of the crystal (space group *P*2_1_, *Z* = 4).

The results obtained with styrenes bearing a substituent at the olefinic bond provide useful information with respect to the reaction mechanism. Thus, the reaction of **1a** with α-methylstyrene or 1,1-diphenylethene proceeded at a faster rate than with styrene and yielded 3-methyl- and 3-phenyl-2-vinylindenes **12b** and **12c** (structure confirmed by an XRD analysis, see Figure 2) in high yields. Benzo[*a*]fluorenes were found to a minor extent (**13c**) or not at all (**13b**) (Scheme 4). A remarkable stereochemical aspect accompanied the reaction of **1a** with (*E*)-1-phenyl-1-propene leading to 2-((*E*)-1-phenylprop-1-enyl)indene **12d**, where *trans(Ph,Me)*→*cis* isomerization at the olefinic bond has occurred. The *E*-configuration was assigned based on NOESY NMR experiments and confirmed by an X-ray structure determination (see Figure 3).

**Scheme 5:**
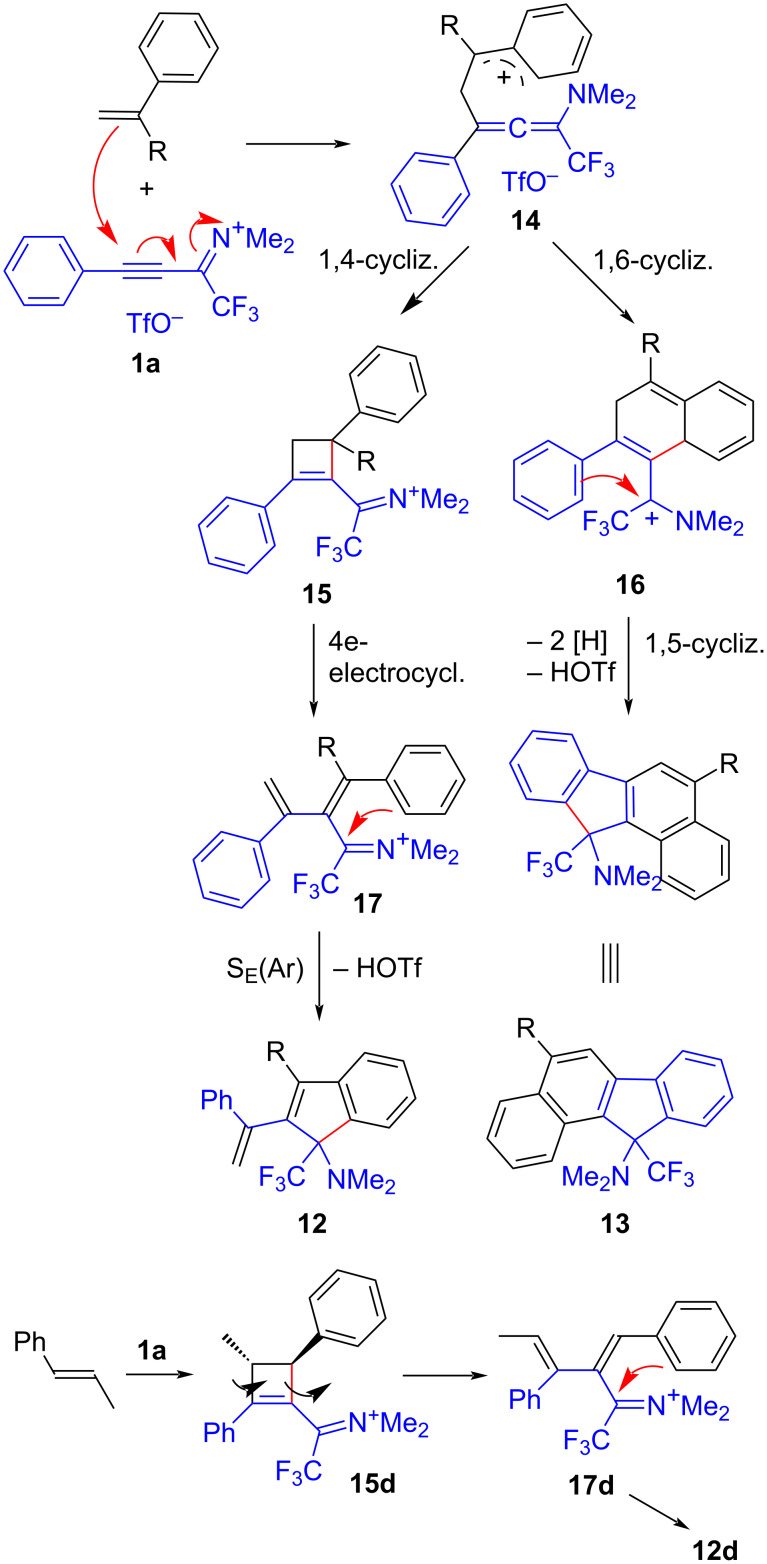
A mechanistic proposal for the reaction of alkyne **1a** with styrenes.

A mechanistic scheme for the formation of indenes **12** and benzo[*a*]fluorenes **13** is proposed in Scheme 5. The electrophilic propyn-1-iminium ion **1a** adds chemoselectively (by conjugate addition) and regioselectively (Markovnikov-type addition) at the olefinic bond of the styrene to form the resonance-stabilized benzyl cation **14** which can add intramolecularly to the nucleophilic central carbon atom of the aminoallene moiety, either by an electrophilic 1,4-cyclization yielding a cyclobutene **15** or by an 1,6-cyclization yielding a dihydronaphthalene **16**. Formally speaking, **15** results from a [2 + 2] cycloaddition and **16** from a [4 + 2] cycloaddition (Diels–Alder reaction). Under the reaction conditions, cyclobutene **15** undergoes a fast electrocyclic ring opening leading to a butadiene **17**, which is finally transformed into 2-(1-phenylvinyl)indene **12** through an intramolecular iminium ion-induced 1,5-cyclization. The same cyclization type together with oxidative aromatization converts dihydronaphthalenes **16** into benzo[*a*]fluorenes **13**. The stereochemistry of **12d** can be explained by a stereoselective formation of *trans*-3,4-disubstituted cyclobutene **15d** and subsequent conrotatory electrocyclic ring-opening, from which *Z*(1,2),*E*(3,4)-configured butadiene intermediate **17d** results.

**Scheme 6:**
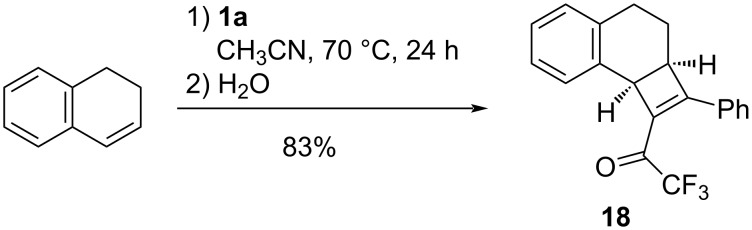
Reaction of alkyne **1a** with 1,2-dihydronaphthalene.

The intermediacy of a cyclobutene **15** in the mechanistic scenario of Scheme 5 is corroborated by the isolation of cyclobutene **18** from the reaction of **1a** with 1,2-dihydronaphthalene, a cyclic styrene derivative (Scheme 6; compare also the cyclobutene byproduct in Scheme 2). The structure of **18** was derived from its ^1^H and ^13^C NMR chemical shifts; a NOE NMR experiment indicated the vicinity of the phenyl ring and the CH_2_CH part or the molecule in line with the expected orientation of the cycloaddition. *Cis*-annelated cyclobutene **18** (^1^H NMR: ^3^*J*_H,H_ = 3.8 Hz for the angular protons) and the iminium-substituted primary cycloadduct are not expected to undergo a fast ring opening under moderate thermal conditions, because the orbital-symmetry-allowed concerted conrotatory process [47–48] would create a strained *cis*,*trans*-dihydrobenzo[8]annulene ring system.

Cyclobutenes resulting from a [2 + 2] cycloaddition of electrophilic alkynes and alkenes under moderate thermal conditions have been isolated also from the reaction of CF_3_-free propyniminium salts with cyclic enol ethers [49] and of other very electrophilic alkynes (i.e., Lewis acid-activated acetylenic esters [50], 1-(trifluoroacetyl)-3-haloacetylenes [44] and 4-chloro-2-oxobut-3-ynoic esters [51–53] with unactivated alkenes (including cyclohexene [51], which did not react with **1a**). For the 3-arylpropyn-1-iminium ions **1**, a charge delocalization can be assumed, which is described by the resonance structure of a 1-aryl-3-aminoallenyl cation, hence their electronic structure shows a certain analogy to the triphenylpropargyl/triphenylallenyl cation. It has been reported that this cation reacts with cyclopentadiene in two different ways: concerted [4 + 2] cycloaddition and a stepwise [2 + 2] cycloaddition via an allenyl-cyclopentenyl cation (which could be trapped with OH^−^) [54–55].

**Scheme 7:**
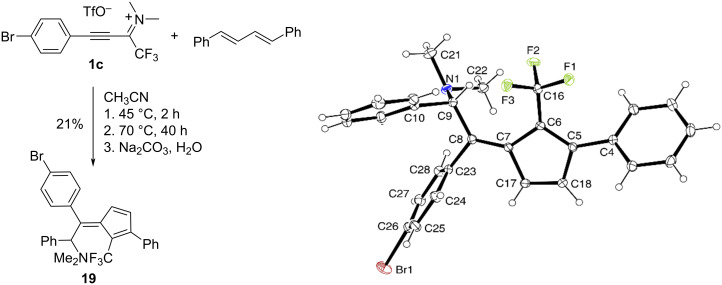
Synthesis and solid-state molecular structure (ORTEP plot) of pentafulvene **19**; selected bond distances (Å), see molecule plot for atom numbering: C8‒C7 1.370(2), C7‒C17 1.481(2), C17‒C18 1.343(2), C18‒C5 1.471(2), C5‒C6 1.374(2), C6‒C7 1.496(2).

Styrene structural moieties are also present in (*E*,*E*)-1,4-diphenylbuta-1,3-diene. Therefore, it was of interest to know whether it would react with propyn-1-iminium salts **1** as a styrene or a 1,3-diene. With 3-(4-bromophenyl)propyn-1-iminium salt **1c** in acetonitrile, no reaction was observed at 20 °C, but within two hours at 45 °C, an unclean reaction took place, which became evident by a multitude of ^19^F NMR signals. Assuming that some of the signals represented products that would easily undergo further thermal reactions, the solution was additionally heated at 70 °C for 40 hours. The resulting reaction mixture still contained several products, of which only the major fluorine-containing component (δ^F^ = −46.76 ppm, a value quite different from those of products **12** and **13**) could finally be isolated in modest yield and was identified by an X-ray structure analysis as the CF_3_-substituted fulvene **19** (Scheme 7).

**Scheme 8:**
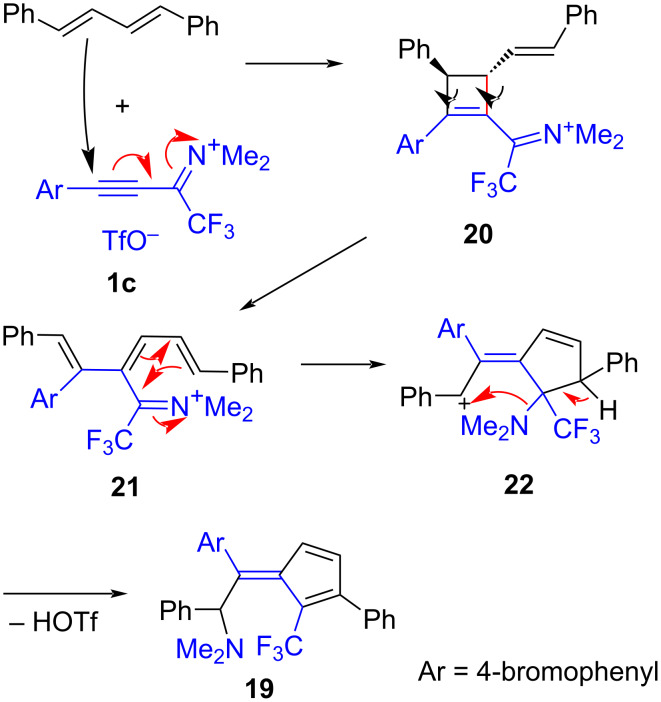
Proposed mechanistic pathway leading to fulvene **19**.

A reaction pathway leading to fulvene **19** is proposed in Scheme 8. It begins with the formal [2 + 2] cycloaddition of **1c** and the diene component, which is probably a two-step process as shown in Scheme 5. Cyclobutene **20** is prone to a thermally induced conrotatory electrocyclic ring-opening, which yields iminium-substituted triene **21**. In a similar reaction, an α-phenyliminium salt structurally analogous to **21** could be isolated [56]. A cationic 1,5-cylization converts **21** into cyclopentene **22**, from which fulvene **19** is formed by deprotonation and a formal 1,4-shift of the NMe_2_ group. The details of this rearrangement are not known, an *N*,*N*-dimethyldihydropyrrolium intermediate may be involved.

## Conclusion

This study has uncovered new applications of 3-aryl-1-(trifluoromethyl)prop-2-yn-1-iminium ions as CF_3_-substituted C_3_ building blocks. They are not only very electrophilic dienophiles in Diels–Alder reactions with normal electron demand (HOMO_diene_‒LUMO_dienophile_ controlled, in the language of FMO theory), but also represent powerful 1,3-biselectrophiles. Thus, Diels–Alder reactions followed by an intramolecular S_E_(Ar) reaction of the α-(trifluoromethyl)iminium functional group were achieved as a two-step one-pot synthesis. On the other hand, an electrophilic (Markownikow type) addition of the propyn-1-iminium ion via its C3-position to the olefinic bond of styrenes initiated a reaction cascade which was again terminated by the already mentioned cyclization step through intramolecular electrophilic aromatic substitution, resulting in the formation of 2-(1-phenylvinyl)-1-(trifluoromethyl)-1-(dimethylamino)indenes as the major products. Various other synthetic applications of these reactive propyn-1-iminium salts will be reported in due course.

## Supporting Information

File 1 Experimental procedures, NMR (^1^H, ^13^C, ^19^F) and IR spectra of synthesized compounds.

File 2 Crystal and structure refinement data for compounds **11**, **12c**, **12d** and **19**.
