# Supplementary material for: Reactions of 3-aryl-1-(trifluoromethyl)prop-2-yn-1-iminium salts with 1,3-dienes and styrenes
Source: Beilstein J Org Chem. 2020 Aug 24;16:2064–72. doi: 10.3762/bjoc.16.173 (PMC7476585; doi:10.3762/bjoc.16.173)
Supplement: File 1 — Experimental procedures, NMR (1H, 13C, 19F) and IR spectra of synthesized compounds. [file Beilstein_J_Org_Chem-16-2064-s001.pdf]

BEILSTEIN JOURNAL OF ORGANIC CHEMISTRY

Supporting Information

for

Reactions of 3-aryl-1-(trifluoromethyl)prop-2-yn-1-iminium salts with 1,3-dienes and styrenes

Thomas Schneider, Michael Keim, Bianca Seitz and Gerhard Maas

Beilstein J. Org. Chem. **2020,** *16,* 2064–2072. doi:10.3762/bjoc.16.173

Experimental procedures, NMR (^{1}H, ^{13}C, ^{19}F) and IR spectra of synthesized compounds

License and Terms: This is a supporting information file under the terms of the Creative Commons Attribution License (https://creativecommons.org/licenses/by/4.0). Please note that the reuse, redistribution and reproduction in particular requires that the authors and source are credited.
The license is subject to the *Beilstein Journal of Organic Chemistry* terms and conditions: (https://www.beilstein-journals.org/bjoc)

Table of Contents

1. Experimental procedures S2

 1.1. Methods and materials S2

 1.2. Reactions of propyn-1-iminium salts **1a,b** with 1,3-dienes S2

 1.3. Reactions of salt **1a** with styrene derivatives S8

2. NMR (^{1}H, ^{13}C, ^{19}F) and IR spectra of synthesized compounds S13

3. References S46

1. Experimental procedures

1.1. Methods and materials

All reactions involving the moisture-sensitive iminium salts were carried out in rigorously dried glassware under an argon atmosphere. Thick-walled Schlenk tubes with a screw cap werre used for all reactions that required elevated temperatures. Solvents were dried by established procedures and stored over molecular sieves (4 Å; 3 Å for acetonitrile) under argon. Column chromatography was performed on silica gel 60 (0.063–0.200 mm). Preparative LPLC: Merck Knauer 50 mL, Merck Lobar® packed column B (310-25), LiChroprep® Si 60 (40–63 µm). Preparative HPLC: Beckman System Gold, Varian Dynamax 250×21.4 mm, Microsorb 100-5 Si. For the LPLC and HPLC separations, the crude product mixtures were first passed through a pad of silica gel in order to remove oligomeric components and inorganic salts, the R_f values reported in this context are those which were observed by TLC using the same eluent. Melting points were determined in open capillaries with a Büchi B-540 instrument at a heating rate of 2 °C/min.

IR spectra of solid samples prepared as KBr pellets or oils between NaCl plates were recorded on a Bruker Vector 22 FT-IR (software: Bruker, Opus NT 2.06) or a Perkin-Elmer Spectrum BX II instrument. Wavenumbers ($\tilde{\nu}_{max}$, cm^{-1}) are reported, relative intensities are given as vs (very strong), s (strong), m (medium) and w (weak). NMR spectra were recorded on a Bruker Avance 400 spectrometer (operating at 400.13 MHz for ^{1}H, 100.61 MHz for ^{13}C, 376.47 MHz for ^{19}F) and a Bruker Avance 500 spectrometer (500.14 MHz for ^{1}H and 125.77 MHz for ^{13}C). NMR chemical shifts (δ) are reported in ppm; for the ^{1}H and ^{13}C spectra the solvent signal served for internal calibration [^{1}H NMR: δ($CHCl_3$) 7.26 (s), ^{13}C NMR: δ($CDCl_3$) 77.16 (t)], for ^{19}F NMR spectra hexafluorobenzene was used (δ(C_6F_6) -164.90). ^{13}C and ^{19}F NMR spectra were recorded in the proton-decoupled mode. When necessary, NMR signal assignments (^{1}H, ^{13}C) were taken from H,H-COSY, C,H-COSY, HMBC und NOESY spectra. Mass spectra were recorded with the following instruments: Finnigan-MAT SSQ-7000 (CI, 100 eV) and SolariX (HRMS: ESI, MALDI), Q Exactive™ Hybrid Quadrupole-Orbitrap (ESI). Propyne iminium salts **1a–c** were prepared as described in lit. [1].

1.2. Reactions of propyn-1-iminium salts 1a,b with 1,3-dienes

2,2,2-Trifluoro-1-(3-phenylbicyclo[2.2.1]hepta-2,5-dien-2-yl)ethan-1-one (3)

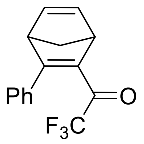

A solution of propyn-1-iminium triflate **1a** (550 mg, 1.47 mmol) in dry acetonitrile (2 mL) was placed in a Schlenk tube flushed with argon, cooled at 0 °C, and cyclopentadiene (430 mg, 6.46 mmol) was added dropwise. The cooling bath was removed and the solution was stirred for additional 2 hours. The formed (norbornadien-2-yl)methanaminium salt **2** (90% yield based on ^{19}F NMR integration) was hydrolyzed in situ with saturated aqueous K_2CO_3 (50 mL). After 10 min, the mixture was extracted with Et_2O (2 × 50 mL) followed by EtOAc (50 mL), the combined organic phases were dried (Na_2SO_4) and the solvents were evaporated. Chromatographic purification (silica gel, eluent cyclohexane/EtOAc (80:1), R_f = 0.79) furnished **3** as a yellow oil (250 mg, 0.95 mmol, 65%). The compound was prone to a slow, undefined decomposition even when stored in a freezer at −18 °C.

^{1}H NMR ($CDCl_3$, 400 MHz): δ = 2.20–2.32 (m, 2H, CH_2), 3.97 (s, 1H, CH), 4.21 (s, 1H, CH), 6.93–7.01 (m, 2H, CH), 7.41–7.43 (m, 3H), 7.62–7.64 (m, 2H).

^{13}C NMR ($CDCl_3$, 101 MHz): δ = 52.17 (q, $^4J_{C,F}$ = 2.7 Hz, C-2), 59.29 (C-4), 70.00 (CH_2), 116.78 (q, $^1J_{C,F}$ = 292.2 Hz, CF_3), 128.12, 128.21, 130.51, 134.79, 140.11, 140.36, 143.93, 177.42 (q, $^2J_{C,F}$ = 34.8 Hz, C=O), 177.93.

^{19}F NMR ($CDCl_3$): δ = -78.09 ppm.

The NMR data agree with the reported ones [Zenova, A. Y.; Borisenko, A. A.; Platonov, V. V.; Proskurnina, M. V.; Zefirov, N. S. *Russ. J. Org. Chem.* **1996**, *32*, 951–954; *Zh. Org. Khim.* **1996**, *32*, 992–995].

IR (NaCl): $\tilde{\nu}$ = 1732 (m), 1691 (s), 1550 (m), 1283 (m), 1205 (vs), 1145 (vs), 1021 (m), 760 (m), 718 (m), 696 (m) cm^{-1}.

MS (CI, 100 eV): *m/z* (%) = 265 (85) $[M+H]^+$, 195 (41) $[M–CF_3]^+$. $C_{15}H_{11}F_3O$ (264.24).

N-(1-(4,5-Dimethyl-3,6-dihydro-[1,1'-biphenyl]-2-yl)-2,2,2-trifluoroethylidene)-*N*-methylmethanaminium triflate (4-Ch)

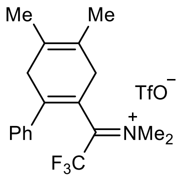

To propyn-1-iminium salt **1a** (551 mg, 1.47 mmol) was added dry CH_3CN (2 mL) and the suspension was cooled to −8 °C. 2,3-Dimethylbuta-1,3-diene (348 mg, 4.24 mmol) was added and after 3 h at this temperature the suspension had changed to a clear solution. After stirring for 20 h at 0 °C, the volatiles were removed at 0.1 mbar to give **4-Ch** (605 mg, 1.32 mmol, 90%) as a dark red powder, which was used without further purification.

^{1}H NMR ($CDCl_3$, 400 MHz): δ = 1.72 (s, 3H, CH_3), 1.74 (s, 3H, CH_3), 2.87–2.94 (m, 1H, CH_2), 3.02–3.26 (m, 3H, CH_2), 3.72 (s, 3H, N^+CH_3), 3.78 (s, 3H, N^+CH_3), 7.09–7.11 (m, 2H, H_{Ph}), 7.44–7.45 (m, 3H, H_{Ph}) ppm.

^{13}C NMR ($CDCl_3$, 101 MHz): δ = 17.89 (CCH_3), 18.15 (CCH_3), 33.49 (CH_2), 38.96 (CH_2), 47.53 (NCH_3), 50.18 (NCH_3), 116.93 (q, $^1J_{C,F}$ = 288.8 Hz, CF_3), 120.65 (q, $^1J_{C,F}$ = 320.0 Hz, TfO^-), 121.5, 122.2, 122.54, 126.05, 129.88, 130.49, 138.44, 148.61, 164.47 (C=N) ppm.

^{19}F NMR ($CDCl_3$): δ = -65.71 (CF_3), -81.48 (TfO^-) ppm.

$C_{19}H_{21}F_6NO_3S$ (457.43). A correct elemental analysis was not obtained.

1-(4,5-Dimethyl-3,6-dihydro-[1,1'-biphenyl]-2-yl)-2,2,2-trifluoroethan-1-one (5-Ch) and 2,2,2-trifluoro-1-(4-methyl-2-phenyl-4-(prop-1-en-2-yl)cyclobut-1-en-1-yl)ethan-1-one (5-Cb)

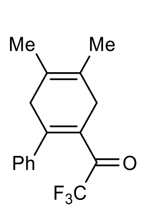

Crude cyclohexadienyliminium salt **4-Ch** (498 mg, 1.09 mmol) was dissolved in dry acetonitrile (2 mL) and cooled to 0 °C. After addition of an aqueous K_2CO_3 solution (8 mL), the mixture was stirred for 15 min followed by extraction with ether. The organic phase was dried (Na_2SO_4) and the volatile components were removed under reduced pressure. Column chromatography of the residue (*n*-hexane/toluene (4:1), R_f = 0.63) furnished a yellowish oil, which consisted of **5-Ch** and **5-Cb** in a 7:1 mole ratio (187 mg, 0.67 mmol, 61%). The two compounds could not be separated.

Cyclohexadiene **5-Ch:** ^{1}H NMR ($CDCl_3$, 400 MHz): δ = 1.70 (s, 3H, CH_3), 1.73 (s, 3H, CH_3), 3.02–3.09 (m, 4H, CH_2), 7.13–7.15 (m, 2H, H_{Ph}), 7.32–7.35 (m, 3H, H_{Ph}) ppm.

^{13}C NMR ($CDCl_3$, 101 MHz): δ = 18.03 (CH_3), 18.07 (CH_3), 34.59 (expanded: q, $^4J_{C,F}$ = 1.1 Hz, F_3C-CO-CCH_2), 40.09 (CH_2), 115.63 (q, $^1J_{C,F}$ = 293.0 Hz, CF_3), 121.99, 122.30, 127.42, 127.87, 128.57, 128.69, 140.35, 146.73, 188.10 (q, $^2J_{C,F}$ = 35.3 Hz, C=O).

^{19}F NMR ($CDCl_3$): δ = -77.66 (CF_3) ppm.

MS (CI, 100 eV): *m/z* (%) = 281 (16) $[M+H]^+$, 280 (18) $[M]^+$, 279 $[M–H]^+$ (100). $C_{16}H_{15}F_3O$ (280.28 g/mol).

Cyclobutene **5-Cb**: ^{1}H NMR ($CDCl_3$): δ = 1.63 (s, 3H), 1.78 (s, 3H), 2.75/2.92 (AB spin system, 2H, $J_{H,H}$ = 15.2 Hz), 4.87–4.89 (m, 2H), 7.43–7.52 (m, 3H), 7.99–8.02 (dd, 2H) ppm. ^{19}F NMR ($CDCl_3$): δ = -78.57 (CF_3) ppm.

1-(4,5-Dimethyl-[1,1'-biphenyl]-2-yl)-2,2,2-trifluoroethan-1-one (6)

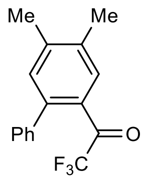

To propyn-1-iminium salt **1a** (912 mg, 2.43 mmol) was added dry CH_3CN (6 mL) and the suspension was cooled to 0 °C. 2,3-Dimethylbuta-1,3-diene (454 mg, 5.54 mmol) was added and after 30 min, the mixture was warmed to rt. After stirring for 2 h, the solution was cooled to 0 °C and *o*-chloranil (599 mg, 2.44 mmol, dissolved in 1 mL of dry CH_2Cl_2) was added and stirring was continued for 20 h. Then, a saturated aqueous K_2CO_3 solution (10 mL) and CH_3CN (5 mL) were added and after stirring for 2 h, the mixture was extraced with ether. The organic phase was dried (Na_2SO_4) and the volatiles were removed under reduced pressure. Filtration of the residue over silica gel (cyclohexane/EtOAc = 20:1) followed by column chromatography (cyclohexane/$CHCl_3$ = 18:5, R_f = 0.43) gave **6** (532 mg, 1.91 mmol, 79%) as a yellow liquid.

^{1}H NMR ($CDCl_3$, 400 MHz): δ = 2.37 (s, 6 H, CH_3), 7.22–7.26 (m, 3 H, H_{Ph}), 7.36–7.42 (m, 3 H, H_{Ph}), 7.53 (s, 1 H, H_{Ph}).

^{13}C NMR ($CDCl_3$, 100 MHz): δ = 19.55 (CH_3), 20.18 (CH_3), 116.23 (q, $^1J_{C,F}$ = 292.7 Hz, CF_3), 127.74, 128.57, 128.86, 129.08, 130.40 (q, $^3J_{C,F}$ = 2.4 Hz, C_{Ph}), 133.02, 136.15, 140.09, 141.64, 142.94, 184.89 (q, $^2J_{C,F}$ = 34.98 Hz, C=O).

^{19}F NMR ($CDCl_3$): δ = -75.64 (CF_3).

IR (NaCl): $\tilde{\nu}$ = 1722 (s, C=O), 1608 (m), 1549 (m), 1484 (m), 1444 (m), 1191 (s), 1145 (s), 1019 (m), 892 (m), 765 (m), 700 (m) cm^{-1}.

MS (CI, 100 eV): *m/z* (%) = 278 (75) $[M]^+$, 209 (77) $[M–CF_3]^+$, 69 (100) $[CF_3]^+$.

Anal. calcd (%) for $C_{16}H_{13}F_3O$ (278.27): C 69.06, H 4.71; found: C 68.92, H 4.95.

N,N,2,3-Tetramethyl-9-(trifluoromethyl)-4,9-dihydro-1*H*-fluoren-9-amine (7)

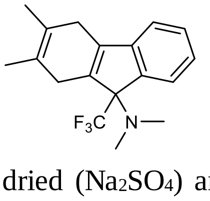

Under an argon atmosphere, propyn-1-iminium salt **1a** (580 mg, 1.55 mmol) was dissolved in dry acetonitrile (2 mL) and 2,3-dimethylbutadiene (339 mg, 4.13 mmol) was added. The solution was stirred at rt for 2 hours, then at 55 °C for 28 hours, and finally allowed to assume room temperature. After addition of aqueous K_2CO_3 (50 mL) and extraction with EtOAc (2 × 50 mL), the combined organic phases were dried (Na_2SO_4) and the volatiles were evaporated. The residual yellow oil was purified by column

chromatography (silica gel, eluent cyclohexane/EtOAc (40:3), R_f = 0.74). Colorless solid (305 mg, 0.99 mmol, 64% yield), m.p. 83 °C.

^{1}H NMR ($CDCl_3$, 400 MHz): δ = 1.78 (s, 6H, CH_3), 2.31 (s, 6H, CH_3), 2.88–2.90 (m, 2H, CH_2), 2.98–3.00 (m, 2H, CH_2), 7.15–7.22 (m, 2H, H_{Ar}), 7.35 (dt, 1H, H_{Ar}), 7.53 (d, $^{3}J_{H,H}$ = 7.4 Hz, 1H, H_{Ar}) ppm.

^{13}C NMR ($CDCl_3$, 101 MHz): δ = 18.91 (CH_3), 19.04 (CH_3), 30.53 (CH_2), 31.33 (CH_2), 40.25 ($N(CH_3)_2$), 77.52 (q, $^{2}J_{C,F}$ = 26.2 Hz), 118.76, 122.14, 123.58, 125.22, 125.72, 125.97 (q, $^{1}J_{C,F}$ = 285.2 Hz, CF_3), 129.22, 137.82, 138.22, 138.54, 145.16 ppm.

^{19}F NMR ($CDCl_3$): δ = -70.89 ppm.

IR (KBr): $\tilde{\nu}$ = 1472 (m), 1447 (m), 1277 (s), 1196 (m), 1186 (m), 1164 (s), 1142 (s), 1096 (m), 1083 (m), 1034 (m), 751 (m), 726 (m) cm^{-1}.

MS (CI, 100 eV): *m/z* (%) = 308 (100) [*M*+H]$^+$, 307 (36), 306 (53), 288 (62).

HRMS (ESI): *m/z* = 308.16197 [*M*+H]$^+$; calcd for $[C_{18}H_{21}F_3N]^+$: 308.16261.

Anal. calcd (%) for $C_{18}H_{20}F_3N$ (307.35): C 70.34, H 6.56, N 4.56; found: C 70.36, H 6.34, N 4.46.

_N,N_,2,3-Tetramethyl-9-(trifluoromethyl)-9_H_-fluoren-9-amine (8)

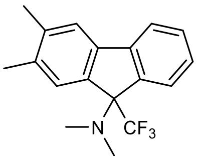

Under an argon atmosphere, dihydrofluorene **7** (476 mg, 1.55 mmol) was dissolved in dry CH_2Cl_2, a solution of *ortho*-chloranil in dry CH_2Cl_2 (2 mL) was added, and the mixture was stirred at rt during 22 hours. After addition of aqueous K_2CO_3 (50 mL) and extraction with Et_2O (50 mL), the combined organic phases were dried (Na_2SO_4), the solvent was evaporated and the residue was purified by column chromatography (silica gel, eluent cyclohexane/EtOAc (20:1), R_f = 0.30). Yellow solid (400 mg, 1.31 mmol, 85% yield), m.p. 77 °C.

^{1}H NMR ($CDCl_3$, 400 MHz): δ = 2.27 (s, 6H, $N(CH_3)_2$), 2.34 (s, 3H, CH_3), 2.35 (s, 3H, CH_3), 7.29 (t, $^{3}J_{H,H}$ = 7.6 Hz, 1H), 7.40–7.45 (m, 2H), 7.47 (s, 1H), 7.64 (d, $^{3}J_{H,H}$ = 7.7 Hz, 1H) ppm.

^{13}C NMR ($CDCl_3$, 101 MHz): δ = 20.30 (CH_3), 20.46 (CH_3), 40.76 (q, $^{4}J_{C,F}$ = 2.0 Hz, $N(CH_3)_2$), 76.00 (q, $^{2}J_{C,F}$ = 27.0 Hz), 119.80, 121.36, 126.32 (q, $^{1}J_{C,F}$ = 282.2 Hz, CF_3), 126.32 (q, $J_{C,F}$ = 1.3 Hz), 127.20, 127.32 (q, $J_{C,F}$ = 1.4 Hz), 129.72, 136.63, 137.84 (q, $J_{C,F}$ = 0.8 Hz), 138.43, 139.48, 140.05 (q, $J_{C,F}$ = 0.7 Hz), 142.04 ppm.

^{19}F NMR ($CDCl_3$ MHz): δ = -71.99 ppm.

IR (KBr): $\tilde{\nu}$ = 1603 (w), 1451 (m), 1284 (m), 1217 (m), 1145 (vs), 1114 (m), 1039 (s), 945 (m), 742 (m) cm^{-1}.

MS (CI, 100 eV): *m/z* (%) = 306 (57) [*M*+H]$^+$, 305 (70), 286 [*M*–F]$^+$ (41), 261 [*M*–$N(CH_3)_2$]$^+$ (55), 236 [*M*–CF_3] (100).

Anal. calcd (%) for $C_{18}H_{18}F_3N$ (305.34): C 70.80, H 5.94, N 4.59; found: C 70.65, H 5.91, N 4.60.

_N,N_,8,9-Tetramethyl-11-(trifluoromethyl)-11_H_-benzo[_a_]fluoren-11-amine (9)

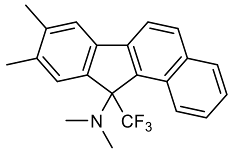

Under an argon atmosphere, alkyne **1b** (437 mg, 1.03 mmol) was dissolved in dry CH_3CN (2 mL), 2,3-dimethylbutadiene (299 mg, 3.64 mmol) was added and the mixture was stirred for 1 hour at rt, then for 22 hours at 50 °C. After cooling to room temperature, addition of aqueous K_2CO_3 (50 mL) and extraction with Et_2O (2 × 50 mL), the combined organic phases were dried (Na_2SO_4) and the volatiles were evaporated. The resulting orange-colored solid (314 mg) was dissolved in dry CH_2Cl_2 (2 mL), *ortho*-chloranil (263 mg, 1.07 mmol) was added, and the mixture was stirred at r.t. for 10 hours. After addition of water (50 mL) and extraction with Et_2O (50 mL), the combined organic phases were dried over Na_2SO_4 and concentrated. The residue was submitted to column chromatography (silica gel, cyclohexane/EtOAc (40:1), R_f = 0.55). Colorless solid (259 mg, 0.73 mmol, 71% yield), m.p. 149 °C.

^{1}H NMR ($CDCl_3$, 400 MHz): δ = 2.38 (s, 6H, CH_3), 2.39 (s, 6H, CH_3), 7.44–7.49 (m, 1H), 7.53–7.58 (m, 3H), 7.79 (d, $^3J_{H,H}$ = 8.3 Hz, 1H), 7.88 (d, $^3J_{H,H}$ = 8.1 Hz, 1H), 7.93 (d, $^3J_{H,H}$ = 8.3 Hz, 1H), 8.85 (d, $^3J_{H,H}$ = 8.6 Hz, 1H) ppm.

^{13}C NMR ($CDCl_3$, 101 MHz): δ = 20.52 (CH_3), 20.84 (CH_3), 40.71 (q, $^4J_{C,F}$ = 2.0 Hz, $N(CH_3)_2$), 79.14 (q, $^2J_{C,F}$ = 27.0 Hz), 118.12, 121.55, 125.69, 125.77 (q, $J_{C,F}$ = 4.0 Hz), 126.65 (q, $^1J_{C,F}$ = 286.1 Hz, CF_3), 126.94, 129.00, 129.06 (q, $J_{C,F}$ = 1.7 Hz), 130.89, 131.37, 134.08, 135.68, 137.03 (q, $J_{C,F}$ = 1.0 Hz), 137.17 (q, $J_{C,F}$ = 0.7 Hz), 138.41, 139.82 (q, $J_{C,F}$ = 0.9 Hz), 140.82 ppm.

^{19}F NMR ($CDCl_3$): δ = -69.34 ppm.

IR (KBr): $\tilde{\nu}$ = 1479 (w), 1454 (w), 1280 (m), 1260 (m), 1217 (m), 1170 (s), 1137 (vs), 1081 (m), 1037 (s), 1028 (s), 821 (m), 759 (m) cm^{-1}.

MS (CI, 100 eV): *m/z* = 356 (53) [*M*+H]$^+$, 355 (100), 311 (79), 286 (34).

Anal. calcd (%) for $C_{22}H_{20}F_3N$ (355.40): C 74.35, H 5.67, N 3.94; found: C 73.96, H 5.79, N 3.90.

_N,N_-Dimethyl-11-(trifluoromethyl)-10,11-dihydro-5_H_-5,10-[1,2]benzenobenzo[_b_]fluoren-11-amine (11)

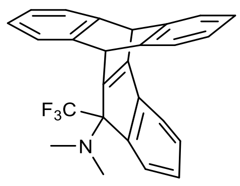

Under an argon atmosphere, anthracene (340 mg, 1.91 mmol) was added to a solution of propyn-1-iminium salt **1a** (698 mg, 1.86 mmol) in acetonitrile (3 mL) and the mixture was stirred at rt for 12 h, then at 55 °C for 2 h (95% conversion into Diels–Alder adduct **10**, which due to its high hydrolytic lability was not isolated, but was identified by ^{1}H NMR; see below). The solvent was replaced by dry toluene (4 mL) and the solution was heated at reflux (105 °C) for 5 days. After cooling to rt, satd aqueous K_2CO_3 was added (50 mL) and the biphasic mixture was extracted with EtOAc (50 mL). The organic phases were combined and dried (Na_2SO_4), then the volatiles were evaporated at reduced pressure. The residue was dissolved in cyclohexane/EtOAc (20:1) and passed over silica gel (≈30 g). Further purification by HPLC (silica gel, cyclohexane/EtOAc (20:1), R_f = 0.45) yielded the product, which was recrystallized from CH_2Cl_2/pentane. Colorless crystals (551 mg, 1.37 mmol, 74% yield), m.p. 120 °C.

Diels–Alder adduct **10**: ^{1}H NMR (CD_3CN, 400 MHz): δ = 3.09 (s, 3H, NCH_3), 3.75 (s, 3H, NCH_3), 5.71 (broadened s, 1H, $C_{sp3}H$), 5.91 (s, 1H, $C_{sp3}H$), 7.07–7.14 (m, 4H), 7.25 (apparent d, 2H), 7.48–7.60 (m, 7H) ppm.

11: ^{1}H NMR ($CDCl_3$, 400 MHz): δ = 2.20 (s, 6H, N(CH_3)$_2$), 5.43 (broadened s, 1H, CH), 5.48 (s, 1H, CH), 6.94–6.98 (m, 2H), 6.98–7.02 (m, 2H), 7.15 (dt, $^3J_{H,H}$ = 7.5 Hz, $^4J_{H,H}$ = 1.1 Hz, 1H), 7.31–7.42 (m, 7H) ppm.

^{13}C NMR (CD_3CN, 101 MHz): δ = 41.39 (q, $^4J_{C,F}$ = 2.0 Hz, NCH_3), 48.80 (CH), 52.01 (q, $J_{C,F}$ = 0.7 Hz, CH), 77.53 (q, $^2J_{C,F}$ = 26.9 Hz), 119.85, 124.14, 124.44, 124.57, 124.68, 125.70, 125.78, 125.81, 125.91, 125.98 (q, $J_{C,F}$ = 1.0 Hz), 126.85 (q, $^1J_{C,F}$ = 284.5 Hz, CF_3), 126.95, 130.35, 140.94, 142.84 (d, $J_{C,F}$ = 1.0 Hz), 146.32, 146.60, 146.76, 147.41, 153.71, 158.81 (q, $J_{C,F}$ = 0.7 Hz) ppm.

^{19}F NMR ($CDCl_3$): δ = -71.13 ppm.

IR (KBr): $\tilde{\nu}$ = 1599 (w), 1456 (m), 1279 (m), 1219 (m), 1147 (s), 1071 (m), 1029 (m), 746 (m), 729 (m), 713 (m) cm^{-1}.

MS (ESI): m/z = 404.16 [M+H]$^+$.

Anal. calcd (%) for $C_{26}H_{20}F_3N$ (403.45): C 77.40, H 5.00, N 3.47; found: C 77.32, H 5.07, N 3.85.

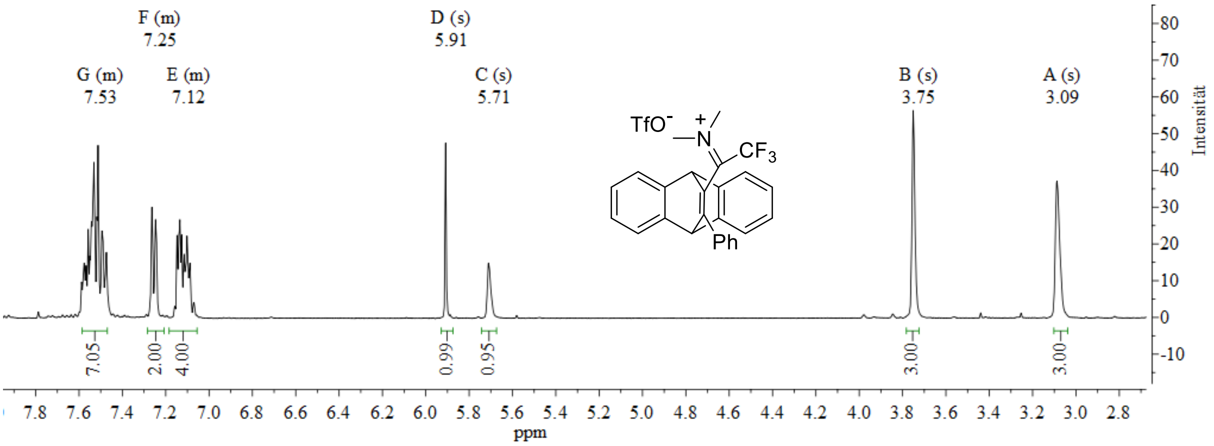

^{1}H NMR spectrum (CD_3CN, 400.13 MHz) of Diels–Alder adduct **10**, obtained from the reaction of **1a** and anthracene (20 °C, 12 h, then 55 °C, 2 h); the product was not purified.

Table: Monitoring of the isomerization **10** → **11** by ^{19}F NMR spectroscopy (see stacked plot of spectra).

Step	Temp-erature	Reaction Time	Solvent	Signal ratio[a] -62.89 ppm	-67.70 ppm	-79.71 ppm
**A**	25 °C	0 h	CH_3CN	1.00	0.00	1.00
**B**	75 °C	7 h	CH_3CN	1.00	0.00	1.00
**C**	75 °C	24 h	CH_3CN	0.98	0.02	1.00
**D**	75 °C	4 d	CH_3CN	0.94	0.06	1.00
**E**	85 °C	2 d	CH_3CN	0.85	0.15	1.00
**F**	105 °C	20 h	toluene	0.48	0.52	1.00
**G**	105 °C	44 h	toluene	0.23	0.77	1.00
**H**	105 °C	5 d	toluene	0.04	0.96	1.00

[a] Signal ratios were obtained by integration; δ-62.89 = **10**, δ-67.70 = *N*-protonated **11**; δ-79.71 = TfO^-.

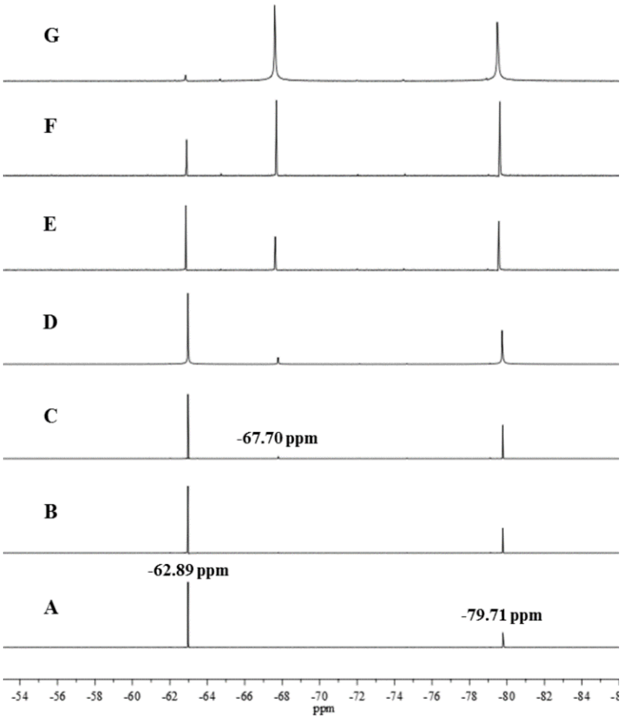

1.3. Reactions of salt 1a with styrene derivatives

General procedure: Under an argon atmosphere, a flame-dried Schlenk tube was charged with propyniminium salt **1a** (1 equiv), dry acetonitrile (10 mL) and styrene or an α- or β-substituted styrene (1 equiv). The stirred solution was heated at 70 °C, until ^{19}F NMR spectra indicated the end of the reaction (12–48 h). After cooling at rt, the reaction mixture was neutralized with saturated aqueous K_2CO_3 (200 mL) and extracted with EtOAc (2 × 150 mL). The combined organic extracts were dried (Na_2SO_4) and the solvent was evaporated. The residue was dissolved in cyclohexane/EtOAc (20:1) and passed over a pad of silica gel (≈30 g), then separated by column chromatography under hydrostatic pressure, LPLC or HPLC.

***N*,*N*-Dimethyl-2-(1-phenylvinyl)-1-(trifluoromethyl)-1*H*-inden-1-amine (12a) and *N*,*N*-dimethyl-11-(trifluoromethyl)-11*H*-benzo[*a*]fluoren-11-amine (13a)**

Prepared from **1a** (770 mg, 2.05 mmol) and styrene (214 mg, 2.05 mmol), 70 °C/48 h. Separation by LPLC (cyclohexane/EtOAc (40:1) yielded **12a** (R_f = 0.60) and **13a** (R_f = 0.39).

12a: colorless solid (459 mg, 1.40 mmol, 68% yield), m.p. 68 °C.

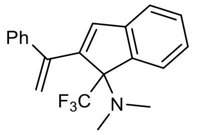

^{1}H NMR ($CDCl_3$, 400 MHz): δ = 2.42 (s, 6H, $N(CH_3)_2$), 5.41 (d, $^2J_{H,H}$ = 2.0 Hz, 1H, CH^A), 6.46 (s, 1H, 3-H_{indene}), 6.57 (broadened signal, 1H of CH^B), 7.18 (d, $^3J_{H,H}$ = 7.4 Hz, 1H), 7.23 (t, $^3J_{H,H}$ = 7.5 Hz, 7.32 (d, $^3J_{H,H}$ = 7.5 Hz, 1H), 7.35–7.39 (m, 5H), 7.61 (d, $^3J_{H,H}$ = 7.5 Hz, 1H) ppm; assignment of $=CH_2$ signals by H,H-COSY.

^{13}C NMR ($CDCl_3$, 126 MHz): δ = 40.04 (q, $^4J_{C,F}$ = 1.7 Hz, NCH_3), 80.27 (q, $^2J_{C,F}$ = 26.8 Hz, C-1), 118.46 (q, $J_{C,F}$ = 1.9 Hz), 122.20, 125.82 (q, $^1J_{C,F}$ = 286.1 Hz, CF_3), 125.90, 126.55 (q, $J_{C,F}$ = 1.4 Hz),

127.49, 128.26, 128.85, 129.37, 135.57 (q, $^{4}J_{C,F}$ = 1.2 Hz, C-3), 139.09, 142.99, 143.30, 143.63, 145.96 ppm.

^{19}F NMR ($CDCl_3$): δ = -69.74 ppm.

IR (KBr): $\tilde{\nu}$ = 1600 (m), 1459 (m), 1278 (m), 1216 (m), 1175 (m), 1141 (s), 1043 (s), 977 (m), 779 (m), 757 (m), 717 (m), 696 (m) cm^{-1}.

MS (ESI): m/z = 330.14 [M+H]$^+$.

Anal. calcd (%) for $C_{20}H_{18}NF_3N$ (329.37): C 72.93, H 5.51, N 4.25; found: C 73.02, H 5.61, N 4.10.

13a: colorless solid (42 mg, 0.13 mmol, 6% yield), m.p. 99 °C.

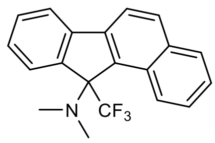

^{1}H NMR ($CDCl_3$, 500 MHz): δ = 2.39 (s, 6H, $N(CH_3)_2$), 7.34 (dt, $^{3}J_{H,H}$ = 7.6 Hz, $^{4}J_{H,H}$ = 1.1 Hz, 1H), 7.47–7.52 (m, 2H), 7.55–7.59 (m, 1H), 7.77–7.81 (m, 2H), 7.82 (d, $^{3}J_{H,H}$ = 8.3 Hz, 1H), 7.89 (d, $^{3}J_{H,H}$ = 7.7 Hz, 1H), 7.95 (d, $^{3}J_{H,H}$ = 8.3 Hz, 1H), 8.87 (d, $^{3}J_{H,H}$ = 8.7 Hz, 1H) ppm.

^{13}C NMR ($CDCl_3$, 125.77 MHz): δ = 40.49 (q, $^{4}J_{C,F}$ = 2.2 Hz, CH_3), 79.13 (q, $^{2}J_{C,F}$ = 27.1 Hz, C-11), 118.02, 120.15, 125.71 (q, $J_{C,F}$ = 4.0 Hz), 125.83, 126.35 (q, $^{1}J_{C,F}$ = 286.2 Hz, CF_3), 126.88, 126.93, 127.63 (q, $J_{C,F}$ =1.5 Hz), 128.85, 129.71, 130.68, 131.34, 134.21, 137.12 (d, $J_{C,F}$ = 1.1 Hz), 139.36, 139.50, 142.77 ppm.

^{19}F NMR ($CDCl_3$): δ = -69.20 ppm.

IR (KBr): $\tilde{\nu}$ = 1579 (w), 1477 (m), 1464 (m), 1261 (m), 1214 (m), 1176 (m), 1144 (s), 1093 (m), 1050 (m), 1035 (m), 825 (m), 765 (m), 754 (m) cm^{-1}.

HRMS (ESI): m/z = 328.13128 [M+H]$^+$; calcd (%) for $[C_{20}H_{17}F_3N]^+$: 328.13131; 283.07332 [M–$N(CH_3)_2$]$^+$, calcd (%) for $[C_{18}H_{10}F_3]^+$: 283.07291.

Anal. calcd (%) for $C_{20}H_{16}F_3N$ (327.12): C 73.38, H 4.93, N 4.28; found: C 72.88, H 5.17, N 4.26.

N,N,3-Trimethyl-2-(1-phenylvinyl)-1-(trifluoromethyl)-1*H*-inden-1-amine (12b)

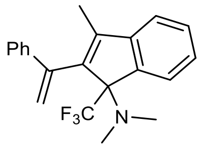

Prepared from salt **1a** (880 mg, 2.35 mmol) and α-methylstyrene (277 mg, 2.35 mmol); 70 °C/12 h. Purification by column chromatography (cyclohexane/EtOAc (10:1); R_f = 0.61). Colorless oil (742 mg, 2.16 mmol, 92% yield), which slowly crystallized on standing.

^{1}H NMR ($CDCl_3$, 500 MHz): δ = 1.69 (s, 3H, CH_3), 2.28 (s, 6H, $N(CH_3)_2$), 5.63 (d, $^{2}J_{H,H}$ = 2.0 Hz, 1H, =CH^A), 6.01 (d, $^{2}J_{H,H}$ = 2.0 Hz, 1H, =CH^B), 7.19–7.24 (m, 3H), 7.24–7.29 (m, 2H), 7.31–7.35 (m, 3H), 7.50 (d, $^{3}J_{H,H}$ = 7.8 Hz, 1H) ppm.

^{13}C NMR ($CDCl_3$, 126 MHz): δ = 12.71 (CH_3), 40.14 (q, $^{4}J_{C,F}$ = 2.1 Hz, NCH_3), 80.20 (q, $^{2}J_{C,F}$ = 26.2 Hz, C-1), 119.39, 119.73, 125.17 (q, $J_{C,F}$ = 1.6 Hz), 125.93 (q, $^{1}J_{C,F}$ = 285.8 Hz, CF_3), 126.21, 127.29, 127.35, 128.38, 129.20, 138.44, 138.95, 142.02 (q, $J_{C,F}$ = 0.8 Hz), 142.61, 143.13, 145.66 ppm.

^{19}F NMR ($CDCl_3$): δ = -69.11 ppm.

IR (NaCl): $\tilde{\nu}$ = 1601 (m), 1570 (m), 1491 (m), 1470 (m), 1382 (m), 1346 (m), 1278 (s), 1216 (s), 1141 (s, broad), 1072 (m), 953 (m), 937 (m), 911 (m), 805 (m), 774 (s), 755 (s), 728 (s), 701 (s) cm^{-1}.

MS (ESI): m/z = 344.16 [M+H]$^+$.

Anal. calcd (%) for $C_{21}H_{20}NF_3N$ (343.39): C 73.45, H 5.87, N 4.08; found: C 72.95, H 6.04, N 4.03.

_N,N_-Dimethyl-3-phenyl-2-(1-phenylvinyl)-1-(trifluoromethyl)-1_H_-inden-1-amine (12c) and _N,N_-dimethyl-5-phenyl-11-(trifluoromethyl)-11_H_-benzo[_a_]fluoren-11-amine (13c)

Prepared from salt **1a** (1.30 g, 3.46 mmol) and 1,1-diphenylethene (624 mg, 3.46 mmol); 70 °C/24 h. After extraction with EtOAc, the solvent was evaporated and the residue was dissolved in *n*-pentane. The major part of indene **12c** precipitated when the solution was concentrated by slow solvent evaporation at rt. The mother liquor was collected by decantation and processed further by HPLC (cyclohexane/EtOAc (40:1)) to furnish **13c** (R_f = 0.52) and another batch of **12c** (R_f = 0.36).

12c: colorless crystals (1.23 g, 3.11 mmol, 90% yield); m.p. 113–114 °C.

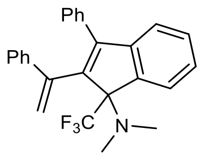

^{1}H NMR ($CDCl_3$, 400 MHz): δ = 2.45 (s, 6H, $N(CH_3)_2$), 5.63 (d, $^2J_{H,H}$ = 2.0 Hz, 1H, =CH^A), 6.36 (d, $^2J_{H,H}$ = 2.2 Hz, 1H, =CH^B), 6.94–7.00 (m, 3H), 7.05–7.20 (m, 8H), 7.27–7.35 (m, 2H), 7.63 (d, $^2J_{H,H}$ = 7.4 Hz, 1H) ppm.

^{13}C NMR ($CDCl_3$, 101 MHz): δ = 40.21 (q, $^4J_{C,F}$ = 2.1 Hz, NCH_3), 80.45 (q, $^2J_{C,F}$ = 26.5 Hz, C-1), 120.61, 121.49, 125.87 (q, $J_{C,F}$ = 1.6 Hz), 126.03 (q, $^1J_{C,F}$ = 285.8 Hz, CF_3), 126.38, 126.82, 127.66, 127.77, 127.94, 127.99, 129.19, 129.26, 134.53, 138.75, 139.70, 141.99, 143.07, 145.06, 145.86 (q, $J_{C,F}$ = 1.1 Hz) ppm.

^{19}F NMR ($CDCl_3$): δ = -68.85 ppm.

IR (KBr): $\tilde{\nu}$ = 1598 (w), 1574 (w), 1492 (w), 1445 (m), 1280 (m), 1212 (m), 1168 (m), 1141 (s), 1073 (w), 1041 (m), 981 (m), 921 (m), 899 (m), 778 (m), 755 (m), 725 (m), 696 (s) cm^{-1}.

MS (CI, 100 eV): *m/z* (%) = 406 (48) $[M+H]^+$, 405 (100), 386 (15), 361(33).

MS (ESI): *m/z* = 406.18 $[M+H]^+$.

Anal. calcd (%) for $C_{26}H_{22}F_3N$ (405.46): C 77.02, H 5.47, N 3.45; found: C 76.95, H 5.59, N 3.52.

13c: colorless solid (89 mg, 0.22 mmol, 6% yield), m.p. 67 °C.

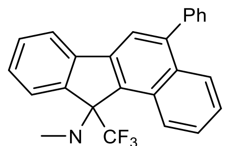

^{1}H NMR ($CDCl_3$, 400 MHz): δ = 2.43 (s, 6H, $N(CH_3)_2$), 7.35 (dt, $^3J_{H,H}$ = 7.6 Hz, $^4J_{H,H}$ = 1.1 Hz, 1H), 7.39–7.44 (m, 1H), 7.45–7.60 (m, 7H), 7.75 (s, 1H), 7.75–7.78 (m, 1H), 7.80 (d, $^3J_{H,H}$ = 7.6 Hz, 1H), 7.89 (d, $^3J_{H,H}$ = 8.4 Hz, 1H), 8.96 (d, $^3J_{H,H}$ = 8.6 Hz, 1H) ppm.

^{13}C NMR ($CDCl_3$, 126 MHz): δ = 40.55 (q, $J_{C,F}$ = 1.9 Hz, CH_3), 79.30 (q, $^2J_{C,F}$ = 27.1 Hz, C-11), 119.19, 120.14, 125.85 (q, $J_{C,F}$ = 3.9 Hz), 125.84, 126.37 (q, $^1J_{C,F}$ = 286.4 Hz, CF_3), 126.65, 127.01, 127.12, 127.63 (q, $J_{C,F}$ = 1.5 Hz), 127.69, 128.47, 129.74, 130.23, 130.99, 132.41, 136.30, 138.72, 139.60, 140.96, 142.66, 143.44 ppm.

^{19}F NMR ($CDCl_3$): δ = -69.03 ppm.

IR (KBr): $\tilde{\nu}$ = 1590 (w), 1472 (w), 1263 (m), 1215 (m), 1147 (s), 1034 (m), 761 (m), 701 (m) cm^{-1}.

HRMS (ESI): *m/z* = 404.16202 $[M+H]^+$; calcd for $[C_{26}H_{21}F_3N]^+$: 404.16261. $C_{26}H_{20}F_3N$ (403.45).

(*E*)-*N*,*N*-Dimethyl-2-(1-phenylprop-1-en-1-yl)-1-(trifluoromethyl)-1*H*-inden-1-amine (12d)

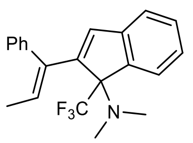

Prepared from salt **1a** (1.05 g, 2.80 mmol) and *trans*-*β*-methylstyrene (331 mg, 2.80 mmol); 70 °C/24 h. LPLC (cyclohexane/EtOAc (40:1), R_f = 0.68)) furnished a colorless solid 560 mg, 1.63 mmol, 58%), m.p. 90 °C.

^{1}H NMR ($CDCl_3$, 500 MHz): δ = 1.63 (dd, $^3J_{H,H}$ = 7.1 Hz, $^5J_{H,H} \approx$ 0.7 Hz, 3H, CH_3), 2.41 (s, 6H, $N(CH_3)_2$), 6.00 (q, $^3J_{H,H} \approx$ 0.8 Hz, 1H, =CH), 7.06 (d, $^3J_{H,H}$ = 7.4 Hz, 1H), 7.12–7.19 (m, 4H), 7.25 (dt, $^3J_{H,H}$ = 7.5 Hz, $^4J_{H,H}$ = 1.1 Hz, 1H), 7.29–7.34 (m, 1H), 7.37–7.42 (m, 2H), 7.54 (d, $^3J_{H,H}$ = 7.0 Hz, 1H) ppm.

^{13}C NMR ($CDCl_3$, 126 MHz): δ = 15.91 (CH_3), 40.12 (q, $^4J_{C,F}$ = 1.8 Hz, $N(CH_3)_2$), 80.18 (q, $^2J_{C,F}$ = 26.6 Hz, C-1), 121.66, 125.18, 125.91 (q, $^1J_{C,F}$ = 286.1 Hz), 126.62 (q, $J_{C,F}$ = 1.8 Hz), 126.87, 127.83 (q, $J_{C,F}$ = 2.5 Hz), 128.36, 129.26, 129.91, 133.25 (q, $J_{C,F}$ = 1.2 Hz), 136.74, 138.85, 140.48, 144.14, 147.77 ppm.

^{19}F NMR ($CDCl_3$ MHz): δ = -69.71 ppm.

IR (KBr): $\tilde{\nu}$ = 1600 (w), 1462 (m), 1441 (m), 1268 (m), 1214 (m), 1149 (vs), 1054 (s), 991 (m), 932 (m), 758 (m), 703 (m) cm^{-1}.

MS (ESI): *m/z* = 344.16 [*M*+H]$^+$.

Anal. calcd (%) for $C_{21}H_{20}F_3N$ (343.39): C 73.45, H 5.87, N 4.08; found: C 73.59, H 5.92, N 4.11.

2,2,2-Trifluoro-1-(2-phenyl-2a,3,4,8b-tetrahydrocyclobuta[*a*]naphthalen-1-yl)ethan-1-one (18)

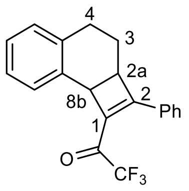

Prepared from salt **1a** (1.05 g, 2.80 mmol) and 1,2-dihydronaphthalene (365 mg, 2.80 mmol); 70 °C/24 h. LPLC (silica gel, cyclohexane/EtOAc (20:1, R_f = 0.60) furnished a very viscous yellow oil (763 mg, 2.32 mmol, 83% yield).

^{1}H NMR ($CDCl_3$, 400 MHz): δ = 1.60–1.70 (m, 1H of $CH_2C\mathit{H_2}CH$), 2.31–2.37 (m, 1H of $CH_2C\mathit{H_2}CH$), 2.50–2.60 (m, 2H, $C\mathit{H_2}CH_2CH$), 3.95–4.00 (apparent t, 1H, 2a-H), 4.47–4.49 (d, $^3J_{H,H}$ = 3.8 Hz, 1H, 8b-H), 7.10 (d, $^3J_{H,H}$ = 7.0 Hz, 1H), 7.15–7.27 (m, 2H), 7.37 (d, $^3J_{H,H}$ = 7.3 Hz, 1H), 7.45–7.55 (m, 3H), 8.03–8.08 (m, 2H) ppm.

^{13}C NMR ($CDCl_3$, 126 MHz): δ = 25.72 (C-3), 26.44 (C-4), 42.00 (C-2a), 43.13 (C-8b), 116.24 (q, $^1J_{C,F}$ = 291.2 Hz, CF_3), 126.42, 126.95, 128.45, 128.86, 129.72, 130.44 (q, $^3J_{C,F}$ =1.7 Hz), 130.72, 131.41, 132.11, 134.92, 139.31, 167.29, 176.13 (q, $^2J_{C,F}$ = 36.1 Hz, C=O) ppm.

^{19}F NMR ($CDCl_3$): δ = -78.32 ppm.

IR (NaCl): $\tilde{\nu}$ = 1690 (s), 1586 (s), 1564 (s), 1491 (m), 1450 (m), 1337 (m), 1265 (m), 1211 (s), 1146 (s), 1112 (m), 1014 (s), 895 (s), 773 (m), 755 (s), 692 (s) cm^{-1}.

MS (ESI): *m/z* = 329.12 [*M*+H]$^+$.

Anal. calcd (%) for $C_{20}H_{15}F_3O$ (328.33): C 73.16, H 4.61; found: C 72.91, H 4.61.

(*Z*)-2-(4-Bromophenyl)-*N*,*N*-dimethyl-1-phenyl-2-(3-phenyl-2-(trifluoromethyl)cyclopenta-2,4-dien-1-ylidene)ethan-1-amine (19)

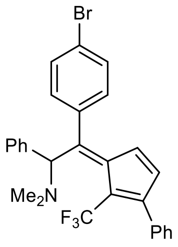

Under an argon atmosphere, (*E*,*E*)-1,4-diphenylbuta-1,3-diene (360 mg, 1.75 mmol) was added to a solution of iminium salt **1c** (792 mg, 1.75 mmol) in dry acetonitrile (4 mL). The diene was not dissolved completely at rt. The stirred mixture was heated at 45 °C for 2 h, then at 70 °C for 40 h. After cooling to rt, it was neutralized with satd aqueous Na_2CO_3 (100 mL), whereby a color change from red to yellow was observed. After extraction with EtOAc (150 mL), the aqueous layer was saturated with NaCl and extracted with EtOAc (100 mL). The organic extracts were combined, dried (Na_2SO_4), and the solvent was evaporated. The oily residue was fractionated by LPLC (eluent cyclohexane/EtOAc (40:1), R_f = 0.78) followed by HPLC (gradient elution with cyclohexane (100 → 90% v/v)/EtOAc, 25 min) to furnish **19**, which was recrystallized from EtOAc/*n*-pentane at r.t. Yellow crystals (185 mg, 0.36 mmol, 21% yield), m. p. 156–156.5 °C.

2. NMR (^{1}H, ^{13}C, ^{19}F) and IR spectra of synthesized compounds

2-Trifluoroacetyl-3-phenylbicyclo[2.2.1]hepta-2,5-diene (3)

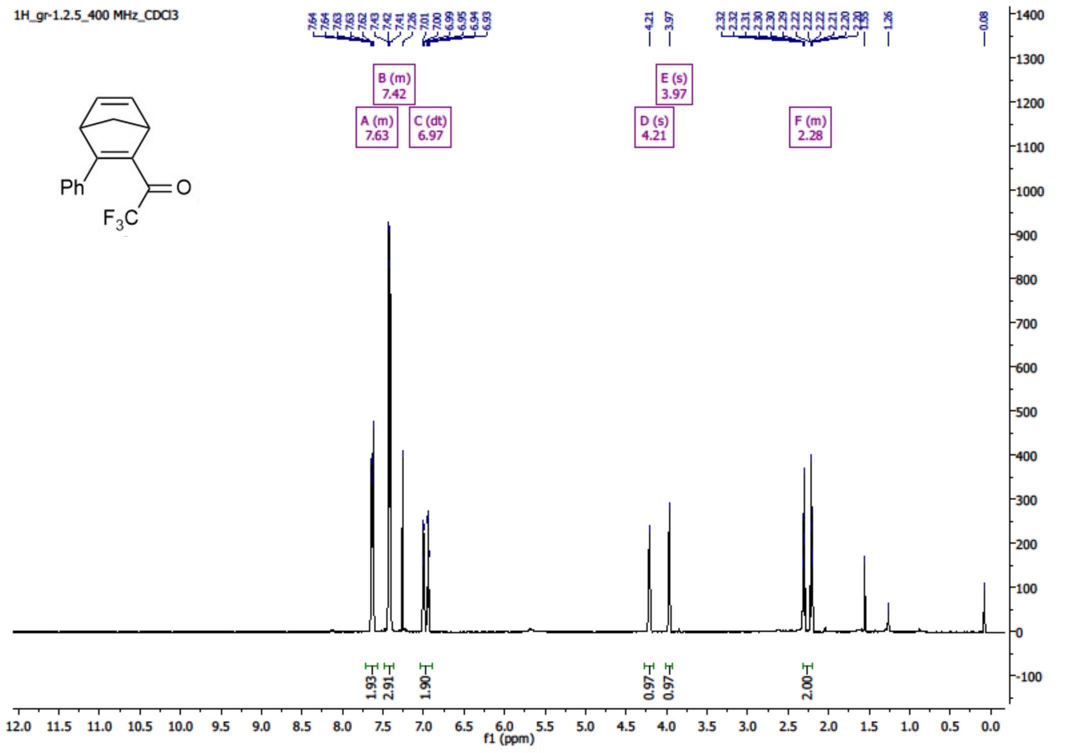

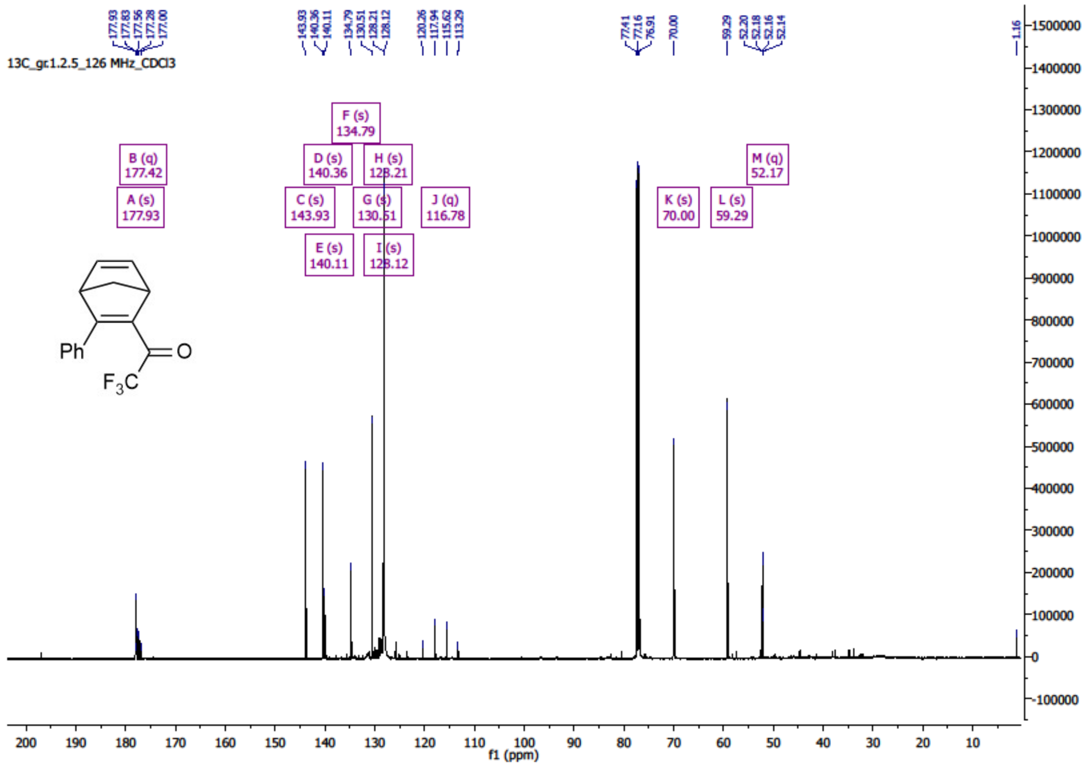

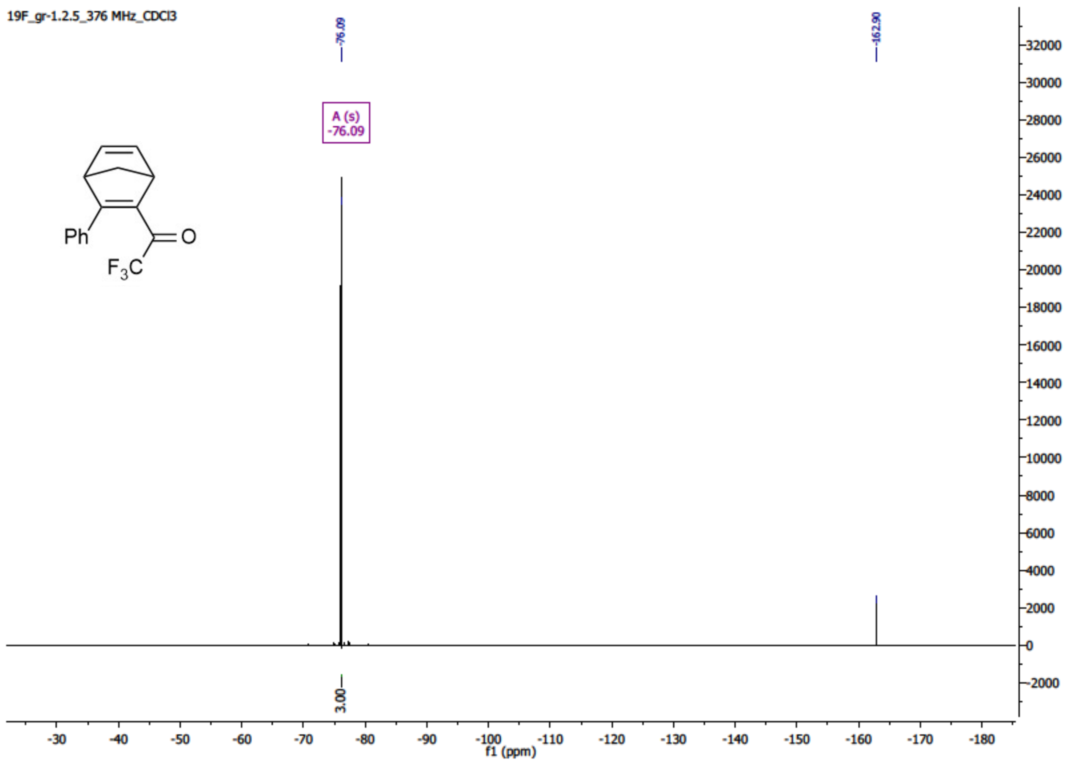
19F_gr-1.2.5_376 MHz_CDCl3
-76.09
-162.90
A (s)
-76.09
3.00
Ph
O
F3C
f1 (ppm)

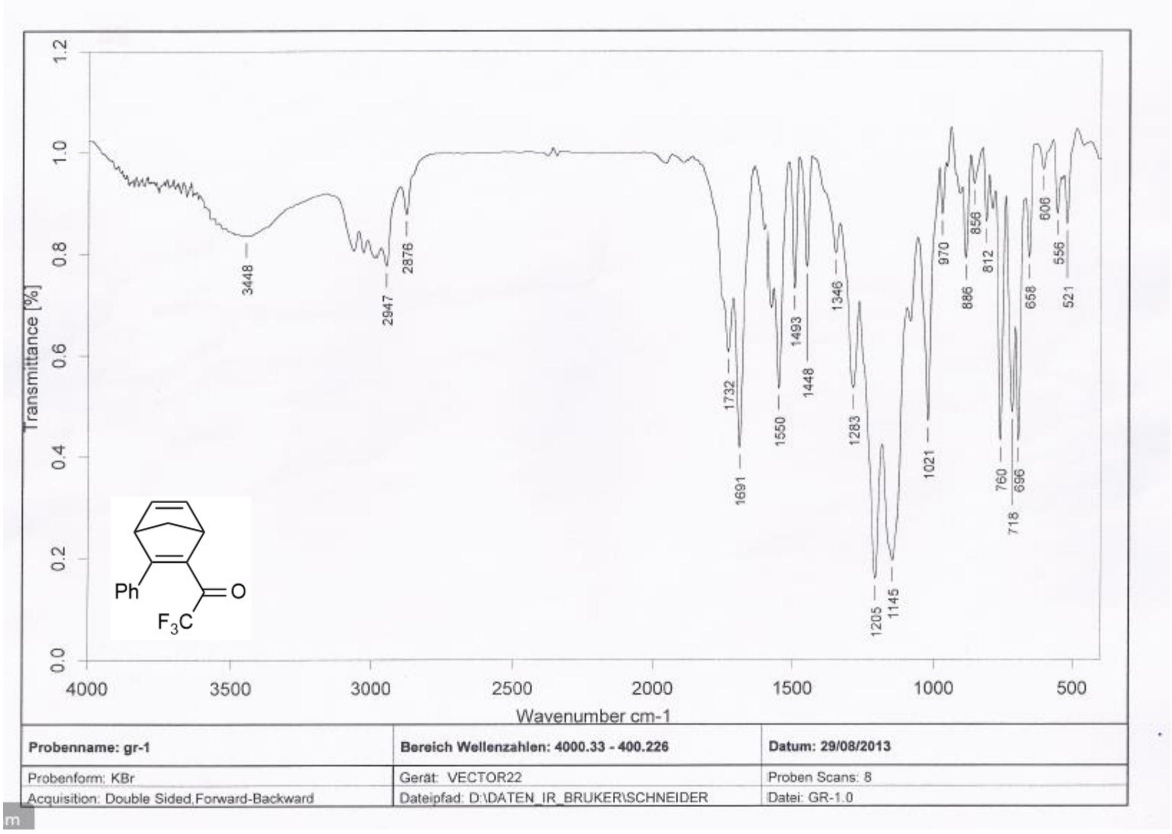
Transmittance [%]
Wavenumber cm-1
3448
2947
2876
1732
1691
1550
1493
1448
1346
1283
1205
1145
1021
970
886
856
812
760
718
696
658
606
556
521
Ph
O
F3C
Probenname: gr-1
Bereich Wellenzahlen: 4000.33 - 400.226
Datum: 29/08/2013
Probenform: KBr
Gerät: VECTOR22
Proben Scans: 8
Acquisition: Double Sided,Forward-Backward
Dateipfad: D:\DATEN_IR_BRUKER\SCHNEIDER
Datei: GR-1.0

N-(1-(4,5-Dimethyl-3,6-dihydro-[1,1'-biphenyl]-2-yl)-2,2,2-trifluoroethylidene)-*N*-methylmethanaminium triflate (4-Ch)

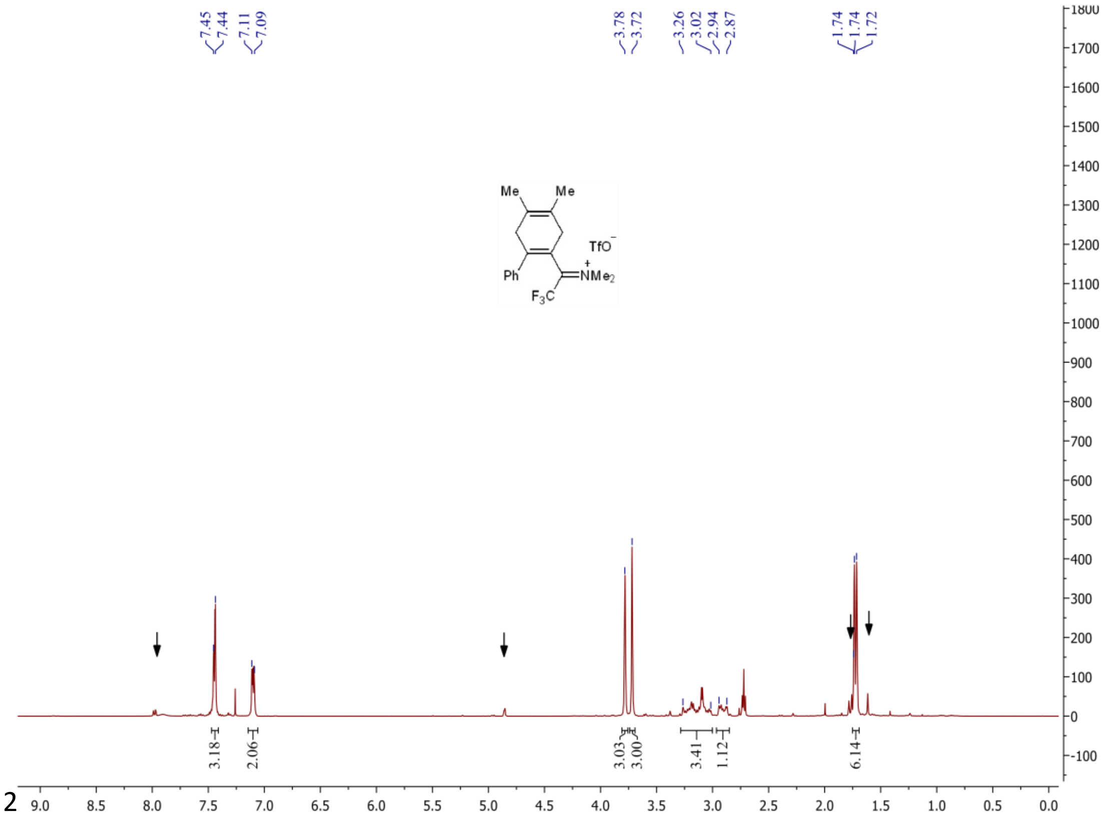

^{1}H NMR ($CDCl_3$, 400 MHz) of crude **4-Ch**. Signals marked with an arrow are attributed to traces of **4-Cb**.

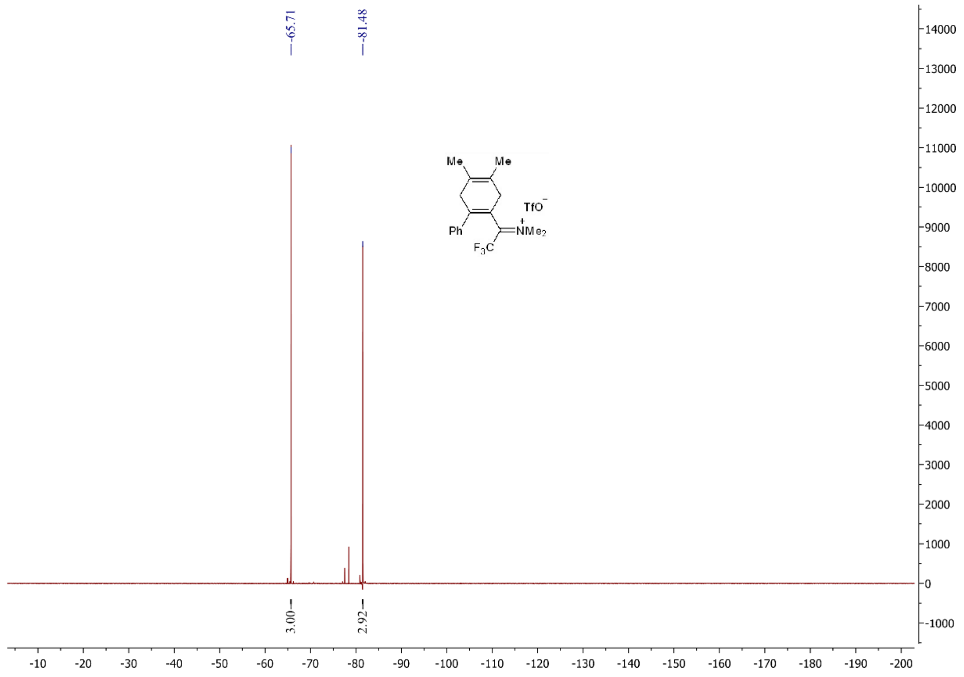

^{19}F NMR($CDCl_3$) of crude **4-Ch**.

1-(4,5-Dimethyl-3,6-dihydro-[1,1'-biphenyl]-2-yl)-2,2,2-trifluoroethan-1-one (5-Ch)

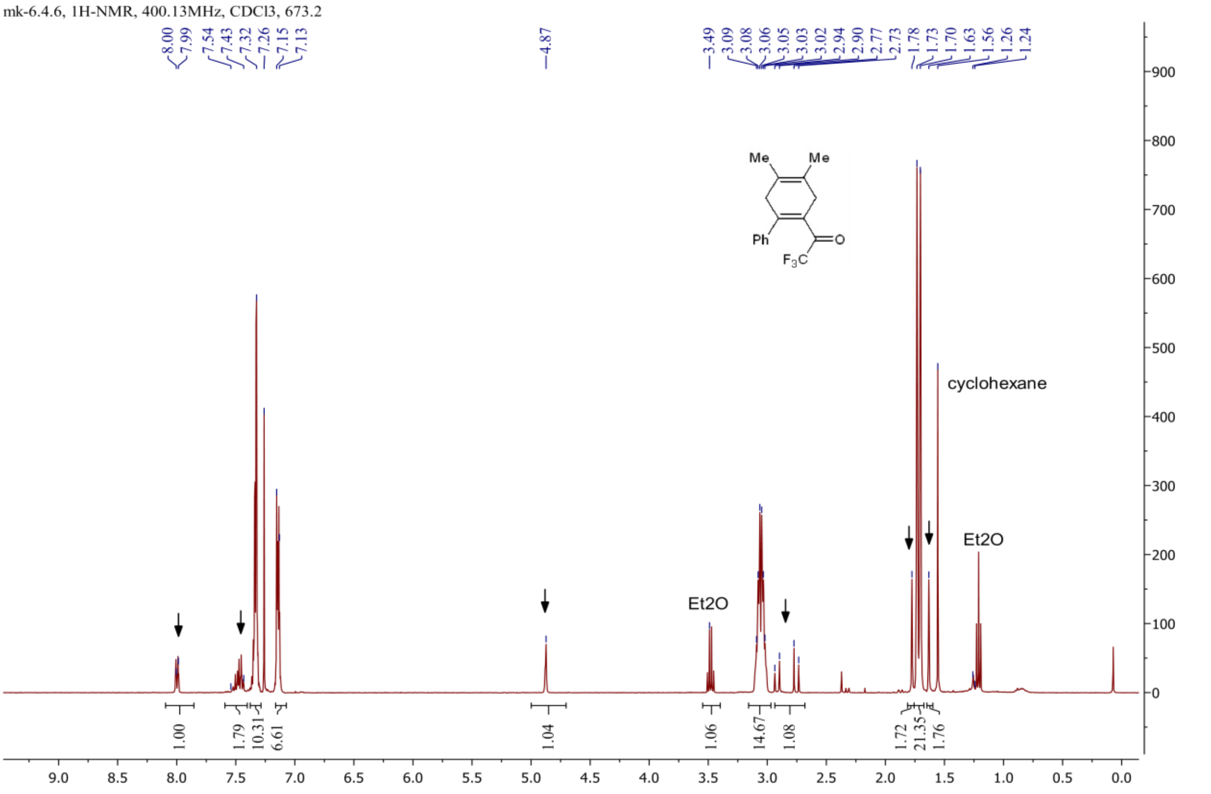

Signals marked with an arrow belong to 2,2,2-trifluoro-1-(4-methyl-2-phenyl-4-(prop-1-en-2-yl)cyclobut-1-en-1-yl)ethan-1-one (**5-Cb**).

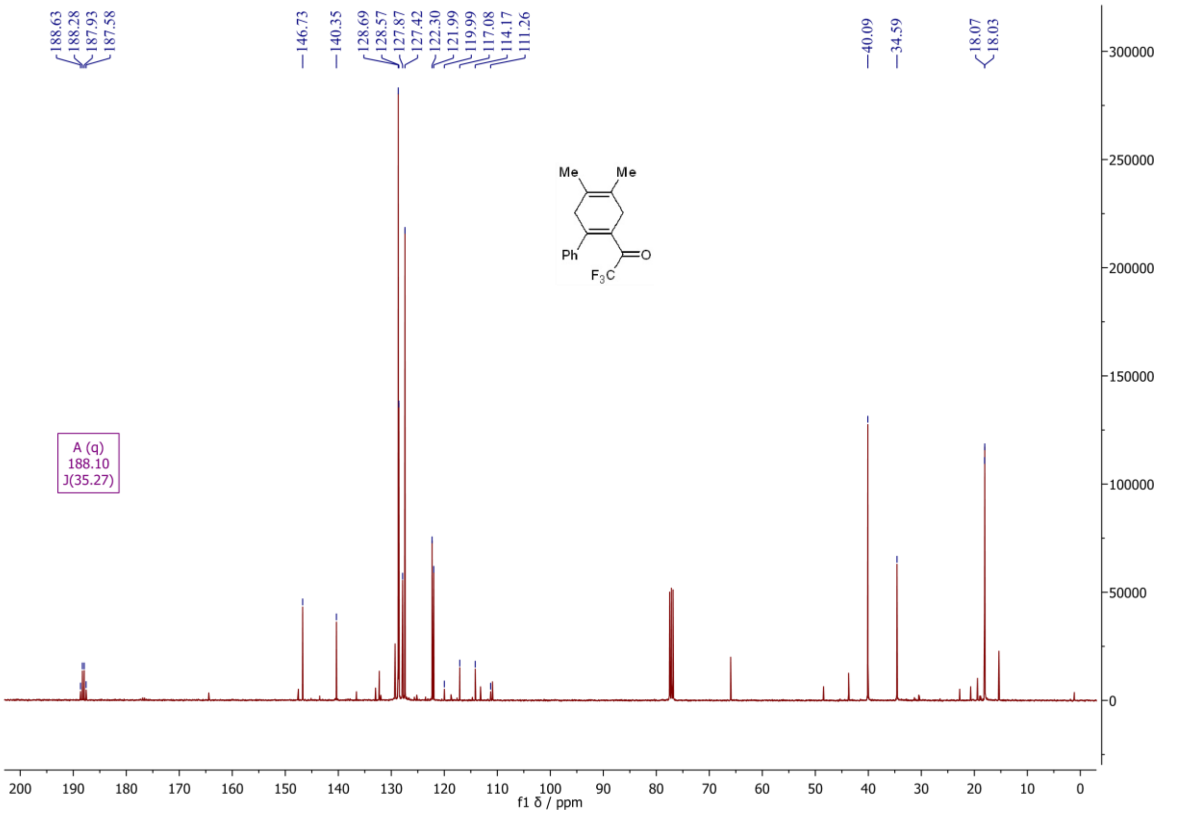

^{13}C NMR ($CDCl_3$, 100.61 MHz) of **5-Ch** containing a small amount of **5-Cb**.

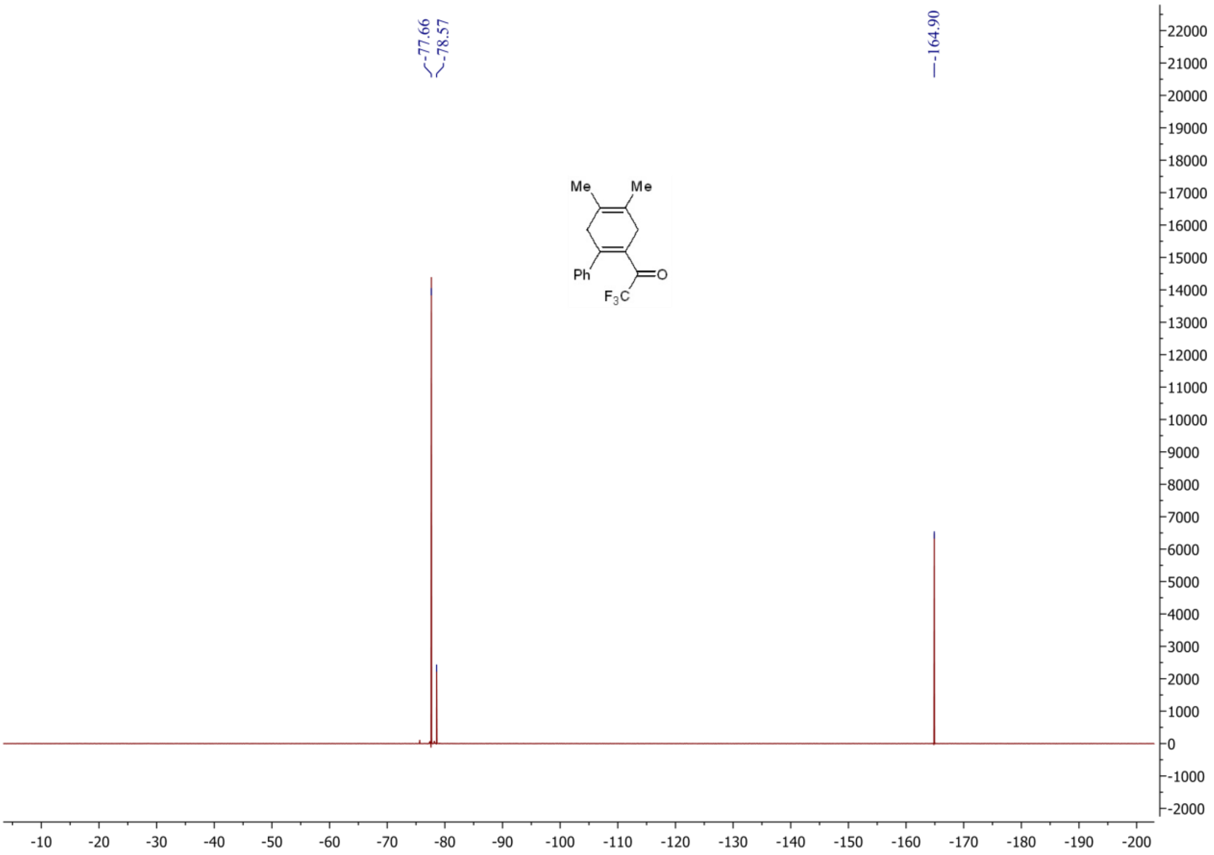

^{19}F NMR ($CDCl_3$): The signal at δ 77.66 ppm belongs to **5-Ch**, the signal at δ 78.57 ppm to **5-Cb**.

1-(4,5-Dimethyl-[1,1'-biphenyl]-2-yl)-2,2,2-trifluoroethan-1-one (6)

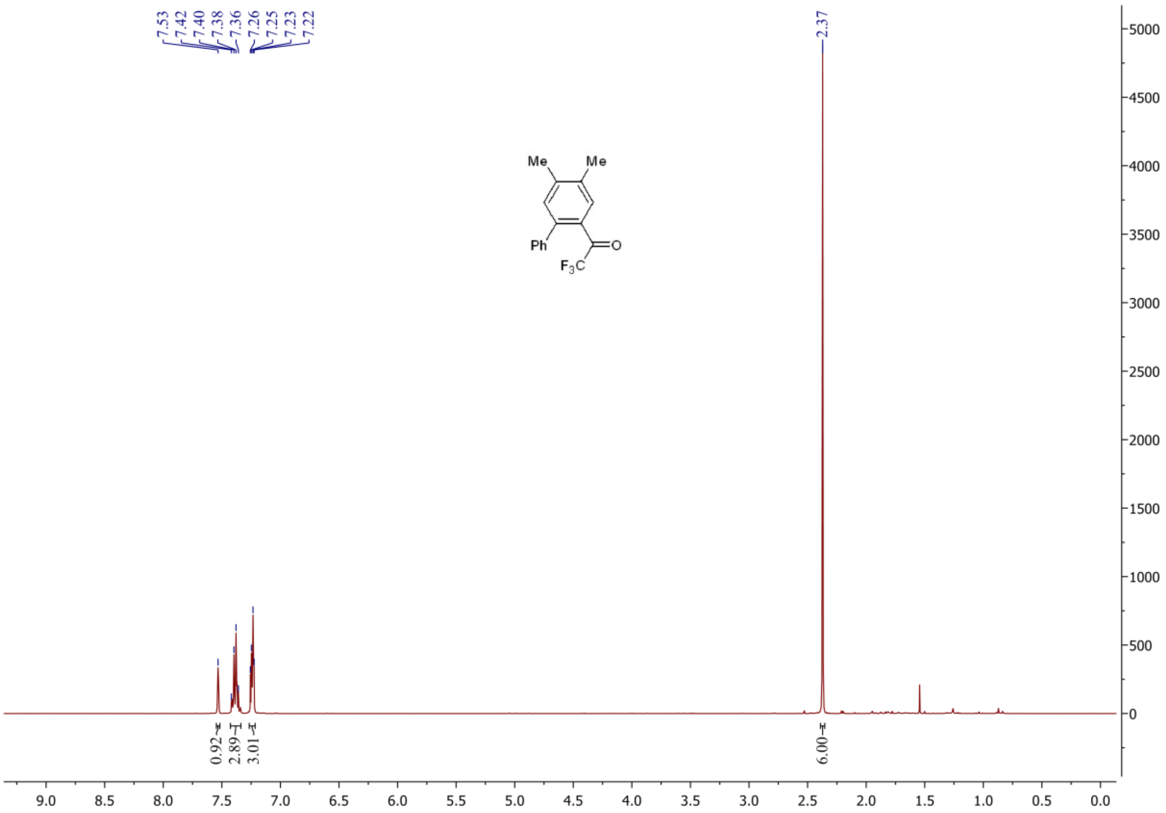

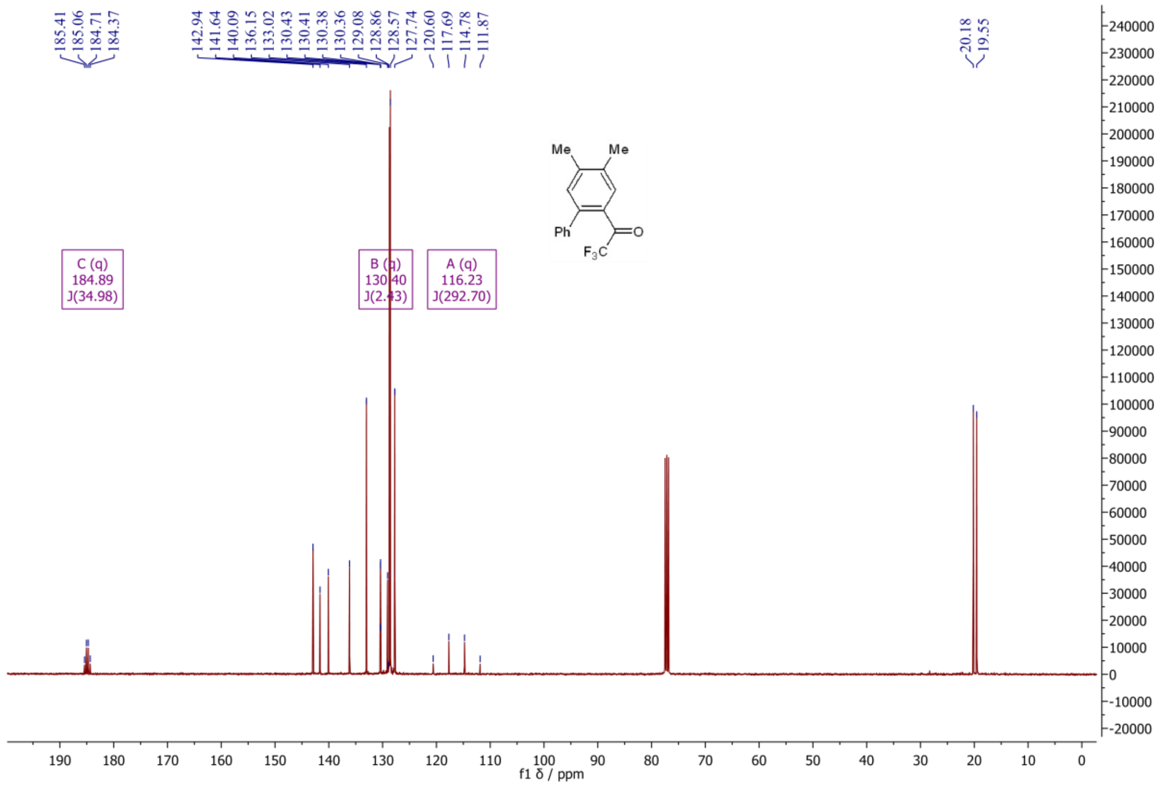

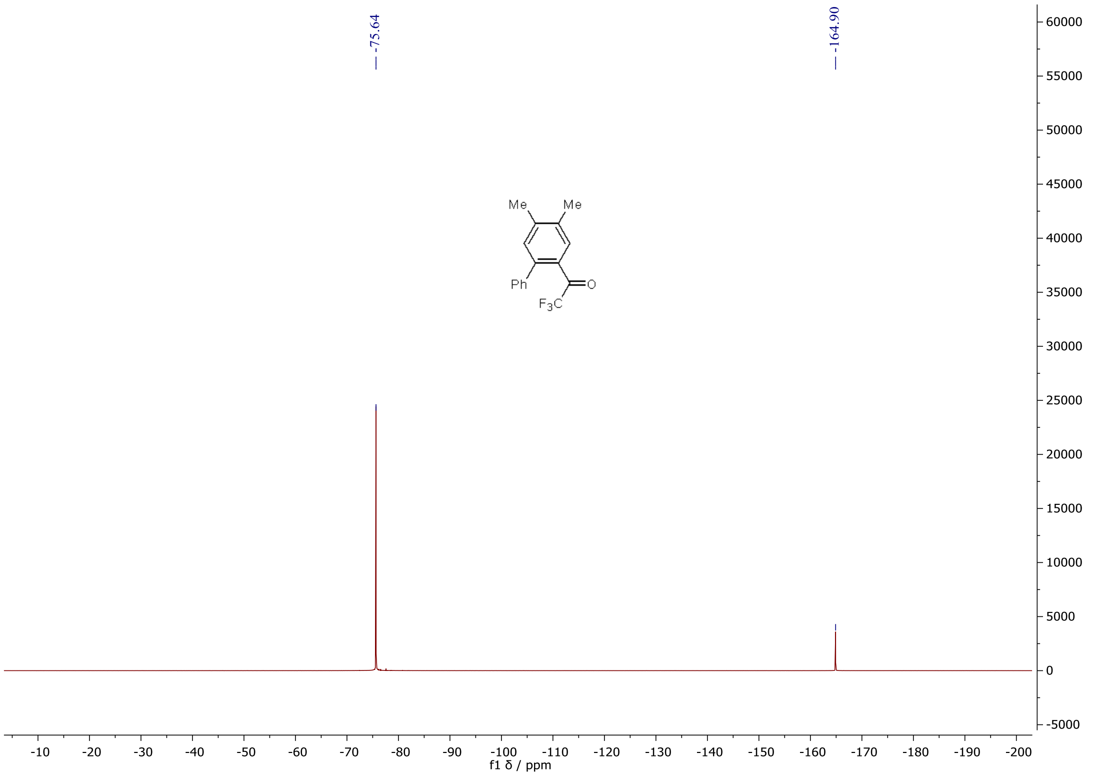

^{19}F NMR ($CDCl_3$) of **6**.

***N*,*N*,2,3-Tetramethyl-9-(trifluoromethyl)-4,9-dihydro-1*H*-fluoren-9-amine (7)**

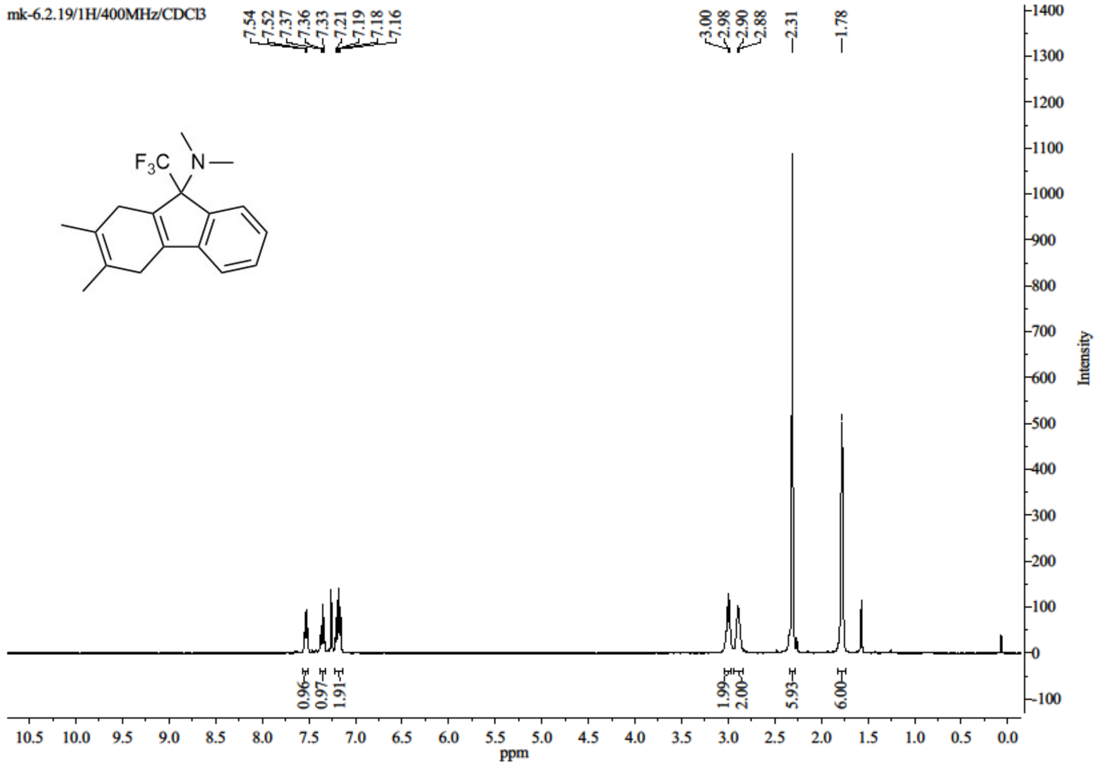

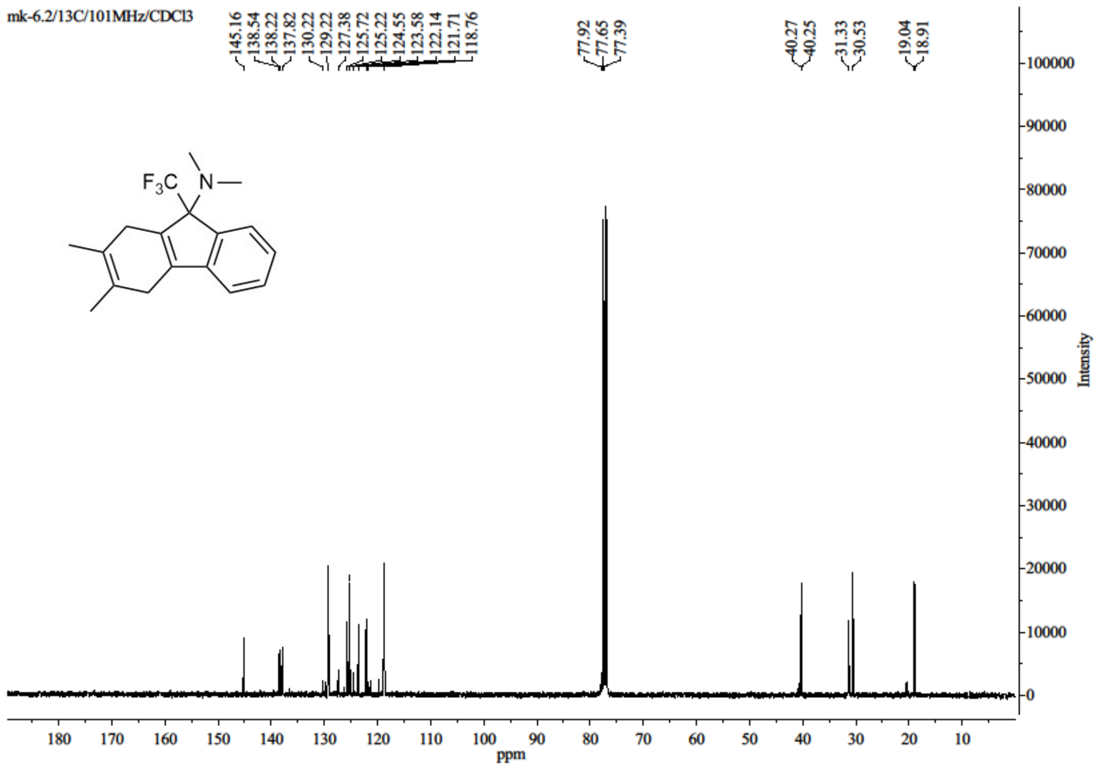

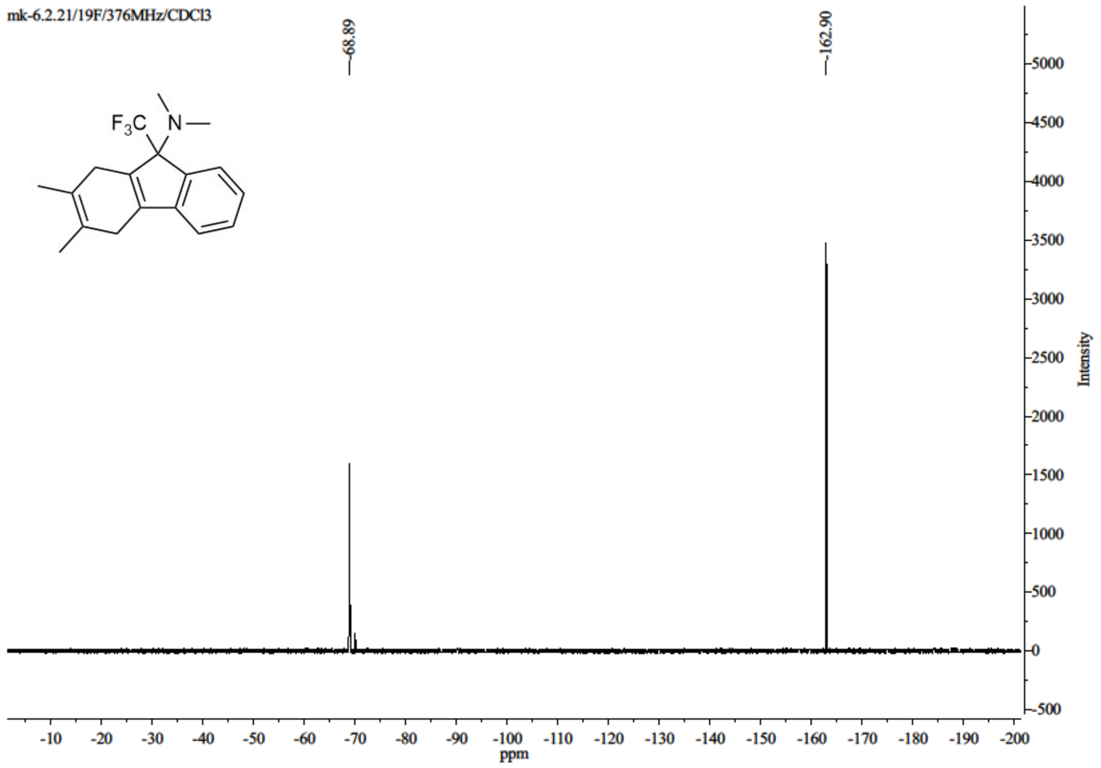
mk-6.2.21/19F/376MHz/CDCl3
-68.89
-162.90
F3C
N
Intensity
ppm

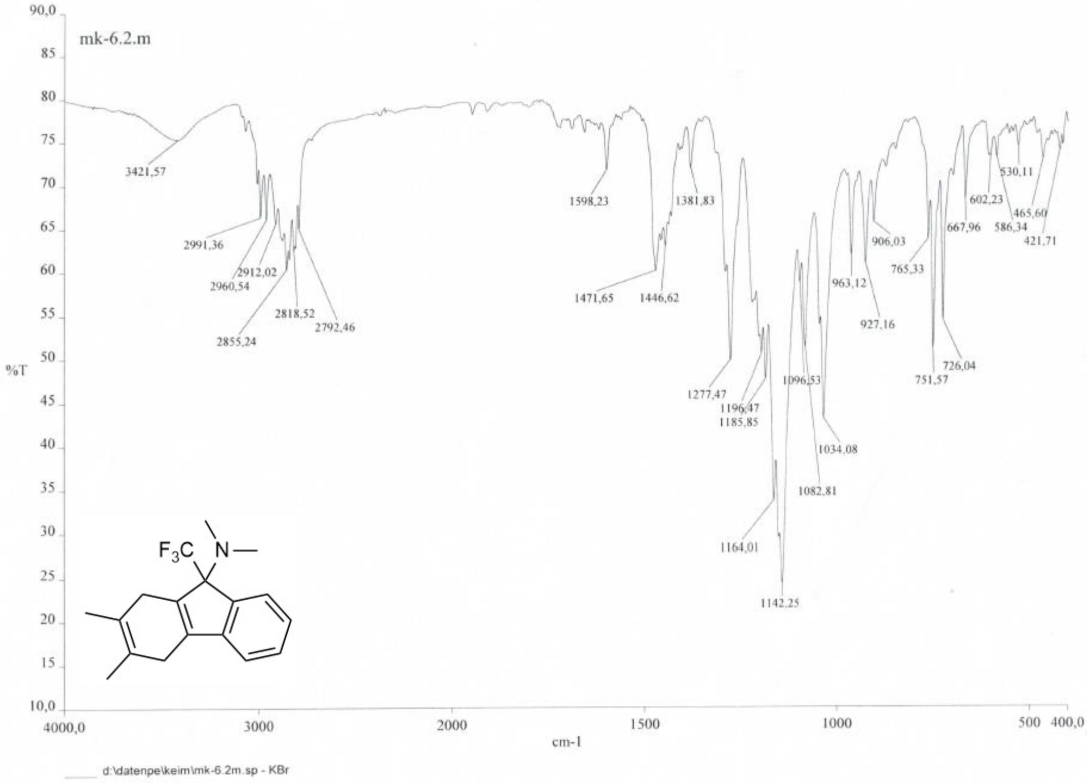
mk-6.2.m
%T
cm-1
3421,57
2991,36
2912,02
2960,54
2855,24
2818,52
2792,46
1598,23
1381,83
1471,65
1446,62
1277,47
1196,47
1185,85
1164,01
1142,25
1096,53
1082,81
1034,08
963,12
927,16
906,03
765,33
751,57
726,04
667,96
602,23
586,34
530,11
465,60
421,71
F3C
N
d:\datenpe\keim\mk-6.2m.sp - KBr

N,N,2,3-Tetramethyl-9-(trifluoromethyl)-9H-fluoren-9-amine (8)

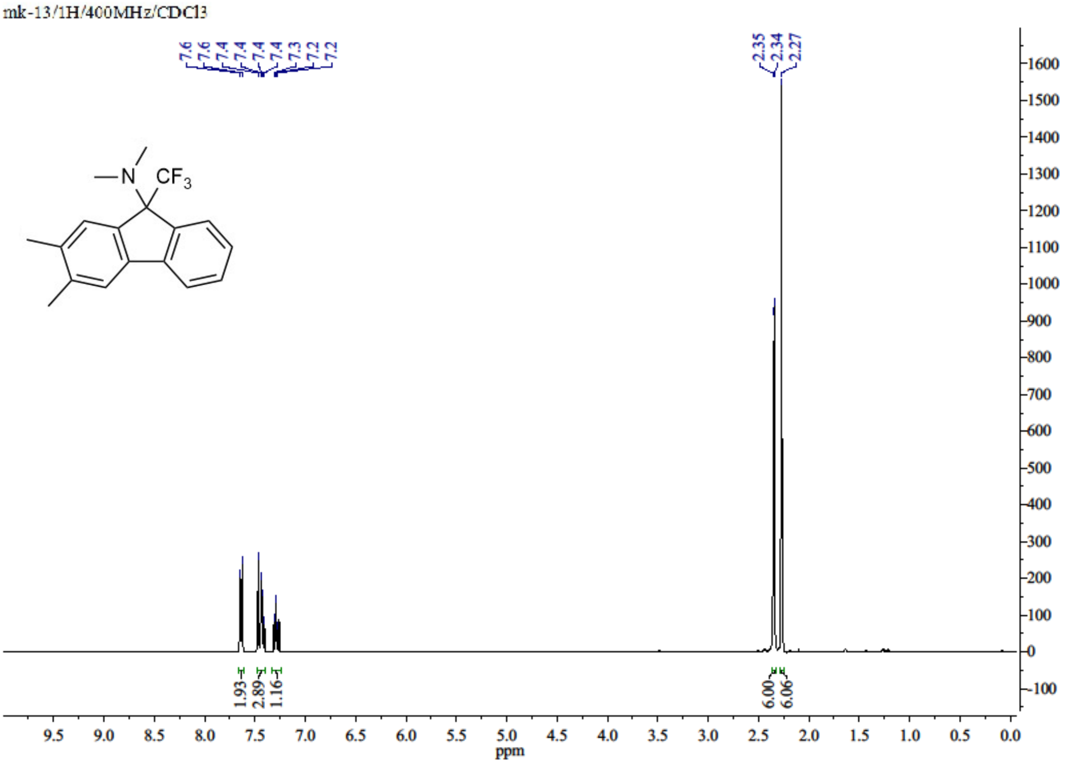

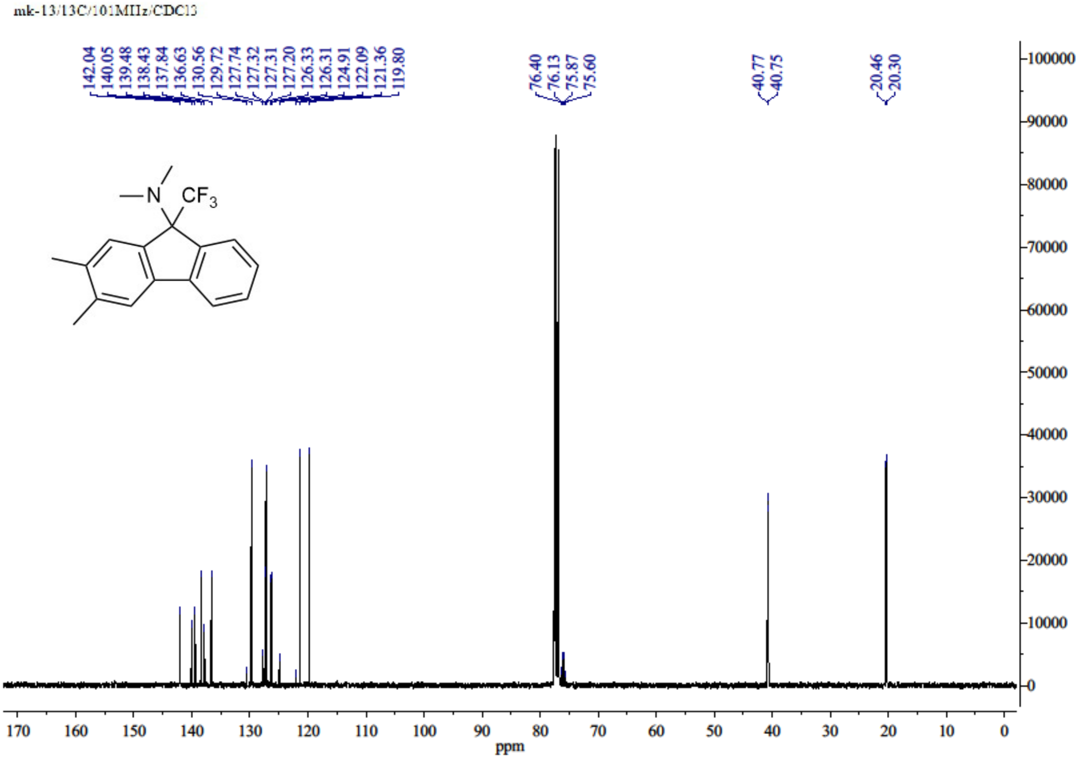

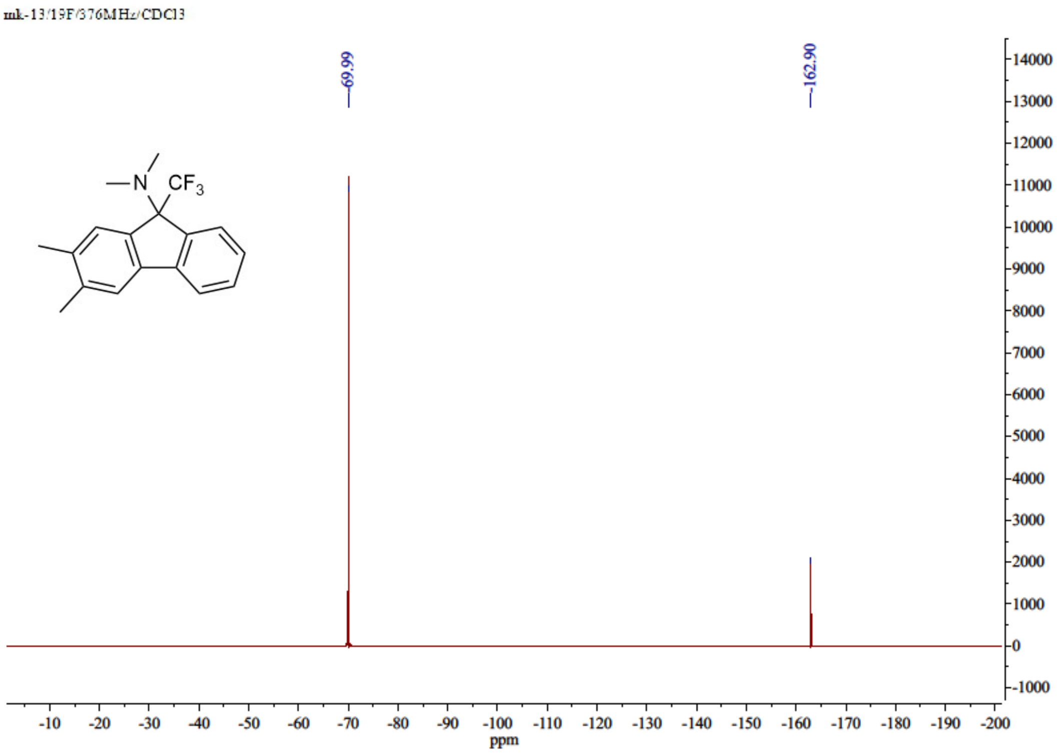

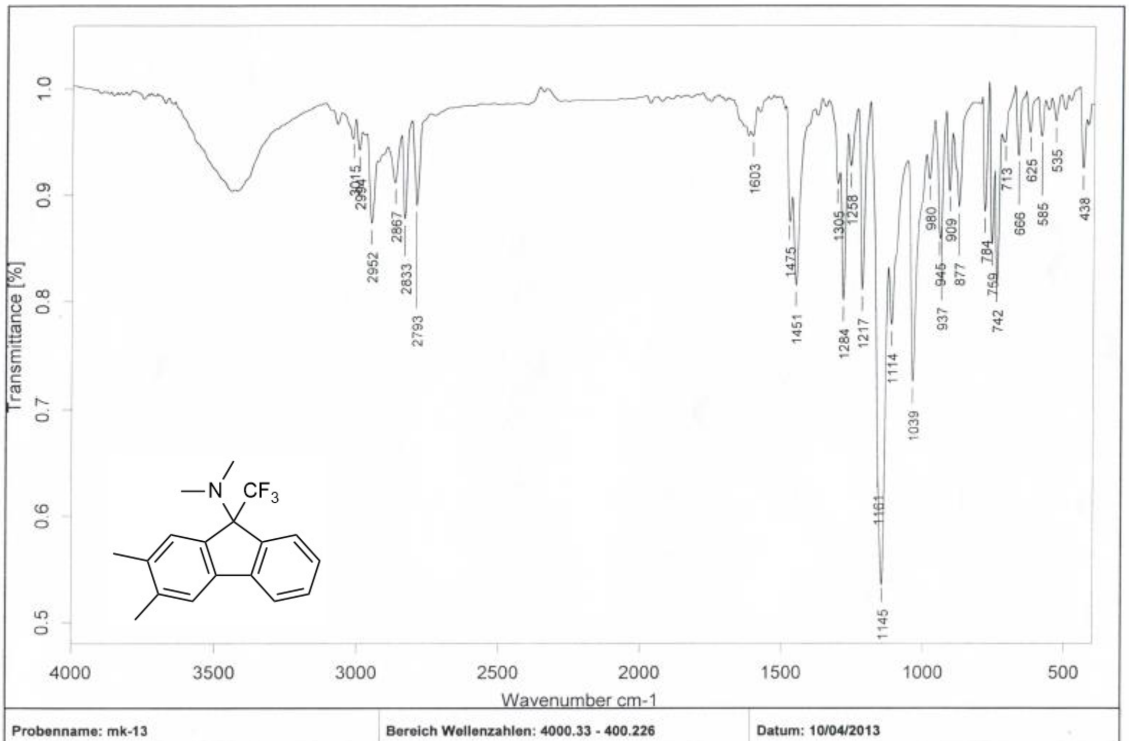

Probenname: mk-13	Bereich Wellenzahlen: 4000.33 - 400.226	Datum: 10/04/2013
Probenform: KBr	Gerät: VECTOR22	Proben Scans: 10
Acquisition: Double Sided,Forward-Backward	Dateipfad: D:\DATEN_IR_BRUKER\SCHNEIDER	Datei: MK-13.1

_N_,_N_,8,9-Tetramethyl-11-(trifluoromethyl)-11_H_-benzo[_a_]fluoren-11-amine (9)

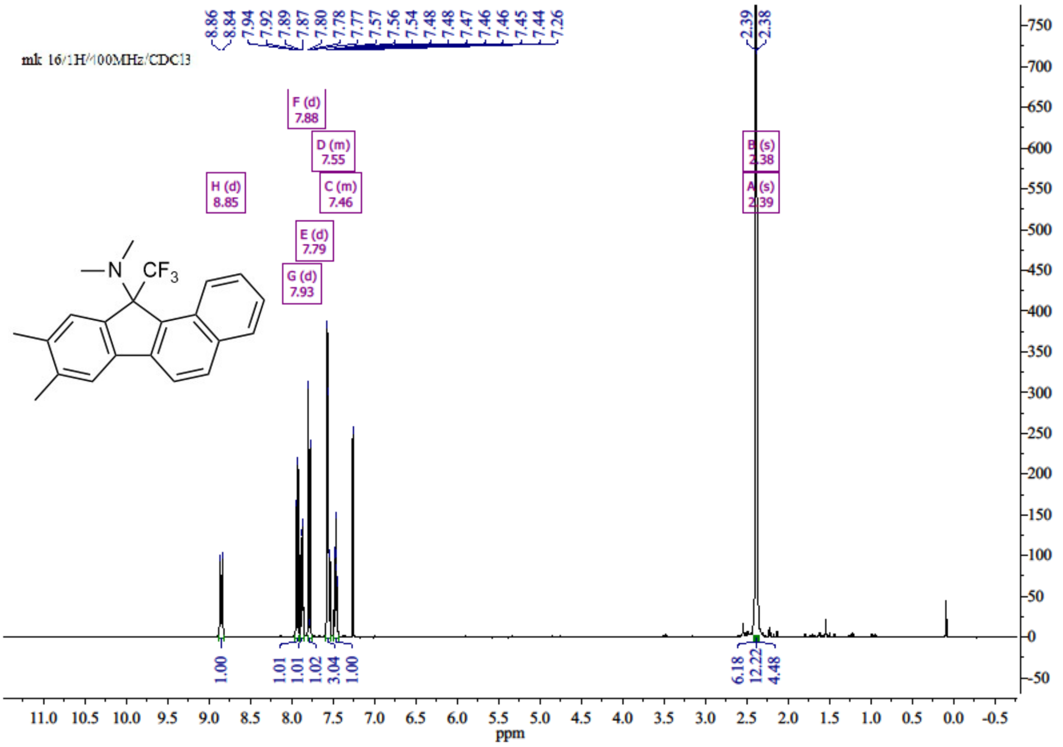

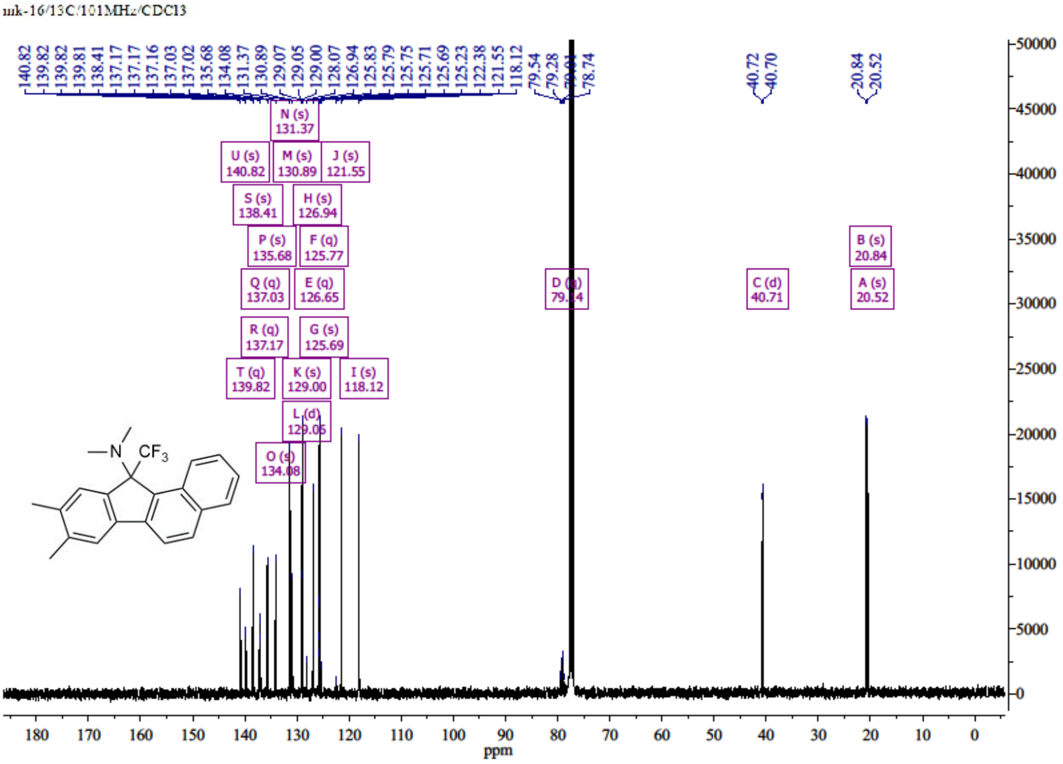

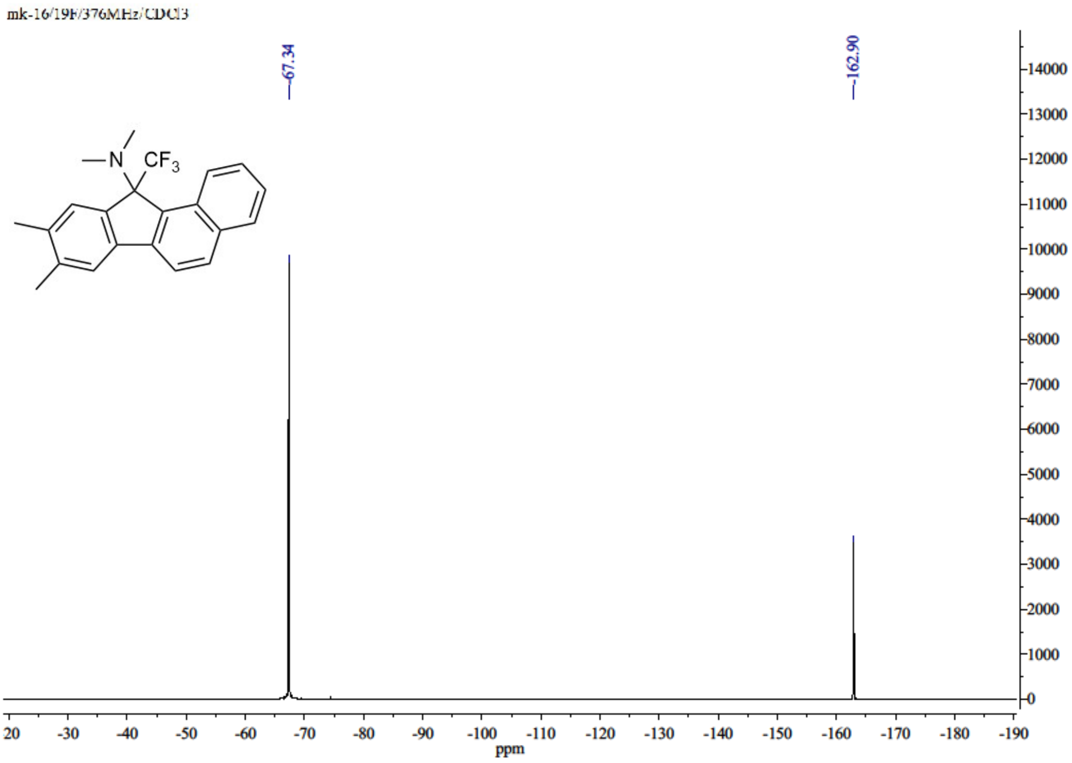
mk-16/19F/376MHz/CDCl3
-67.34
-162.90
N
CF3
ppm

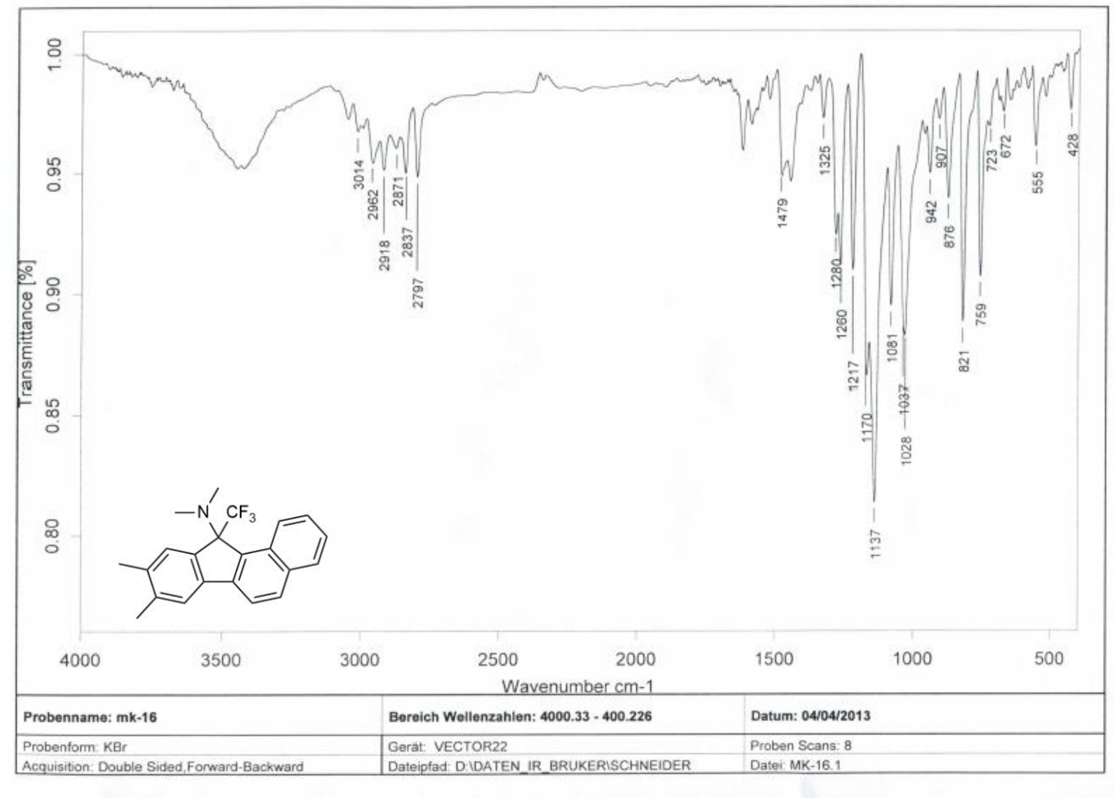
Transmittance [%]
Wavenumber cm-1
N
CF3
3014
2962
2918
2871
2837
2797
1479
1325
1280
1260
1217
1170
1137
1081
1037
1028
942
907
876
821
759
723
672
555
428
Probenname: mk-16
Bereich Wellenzahlen: 4000.33 - 400.226
Datum: 04/04/2013
Probenform: KBr
Gerät: VECTOR22
Proben Scans: 8
Acquisition: Double Sided,Forward-Backward
Dateipfad: D:\DATEN_IR_BRUKER\SCHNEIDER
Datei: MK-16.1

***N*,*N*-Dimethyl-11-(trifluoromethyl)-10,11-dihydro-5*H*-5,10-[1,2]benzenobenzo[*b*]fluoren-11-amine (11)**

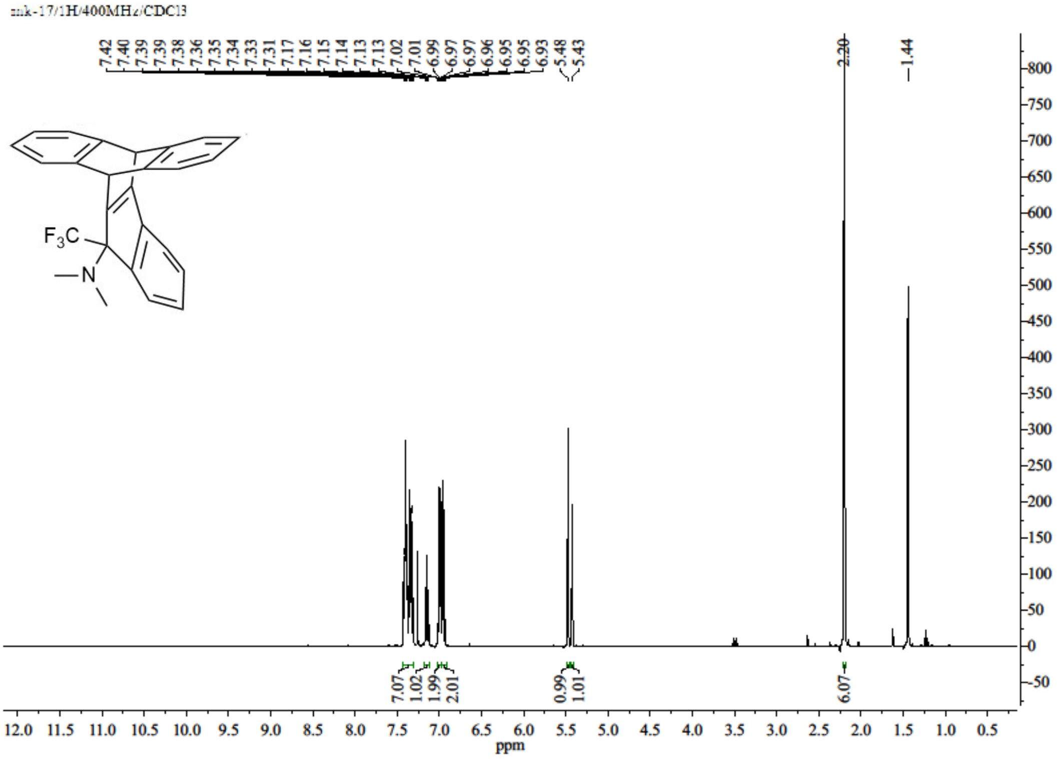

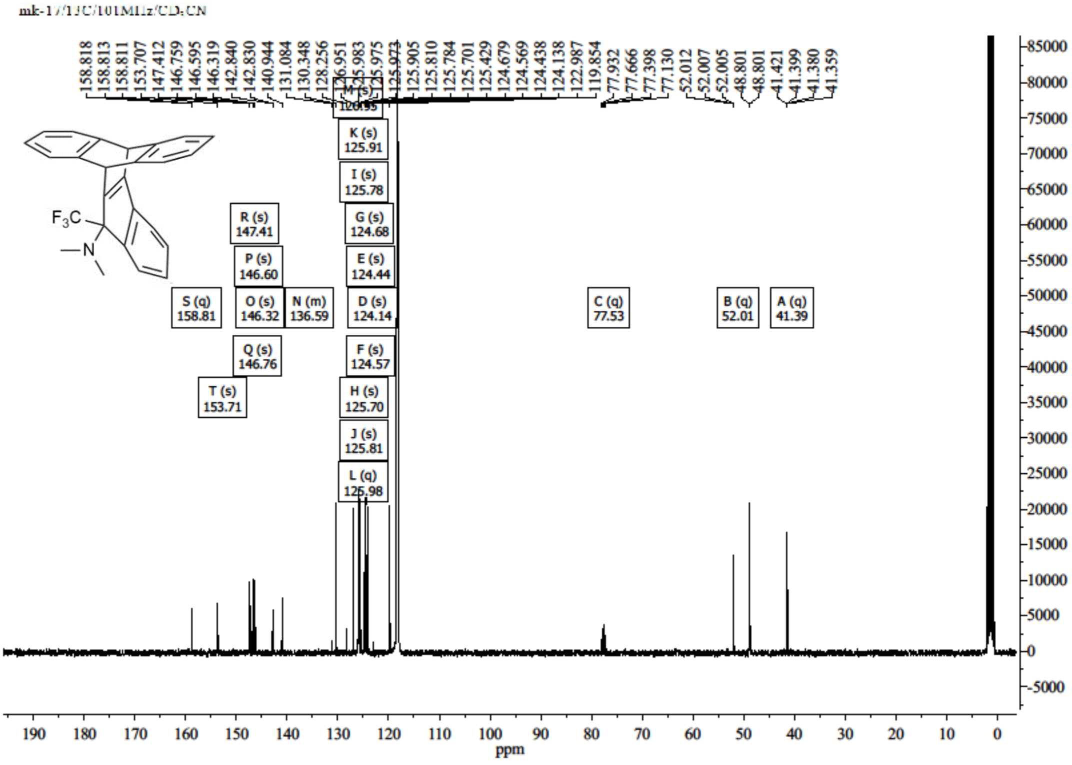

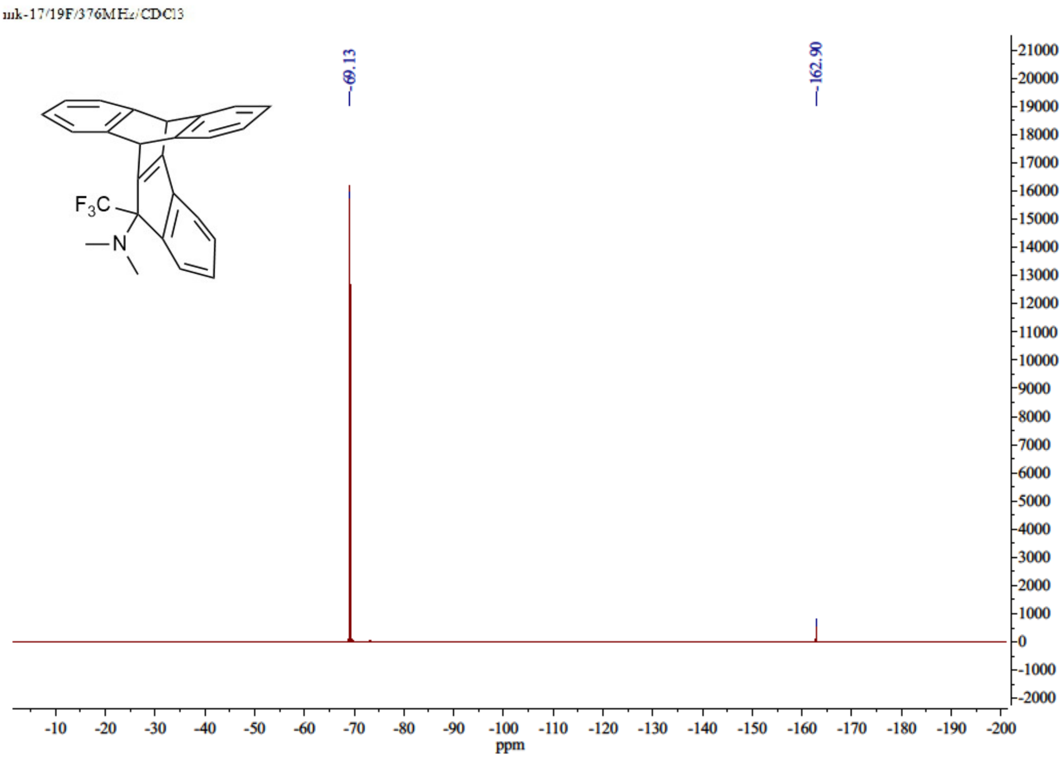

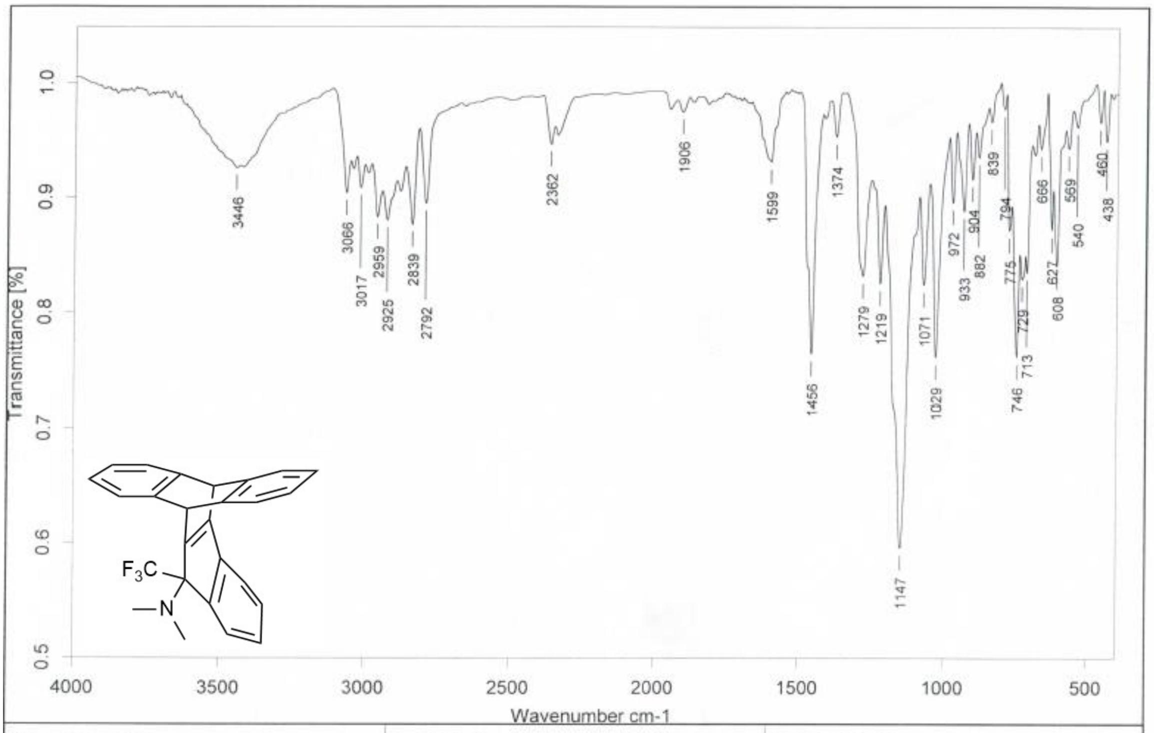

Probenname: mk-17	Bereich Wellenzahlen: 4000.33 - 400.226	Datum: 23/04/2013
Probenform: KBr	Gerät: VECTOR22	Proben Scans: 8
Acquisition: Double Sided,Forward-Backward	Dateipfad: D:\DATEN_IR_BRUKER\SCHNEIDER	Datei: TJ-TEST4.3.1.1

N,N-Dimethyl-2-(1-phenylvinyl)-1-(trifluoromethyl)-1H-inden-1-amine (12a)

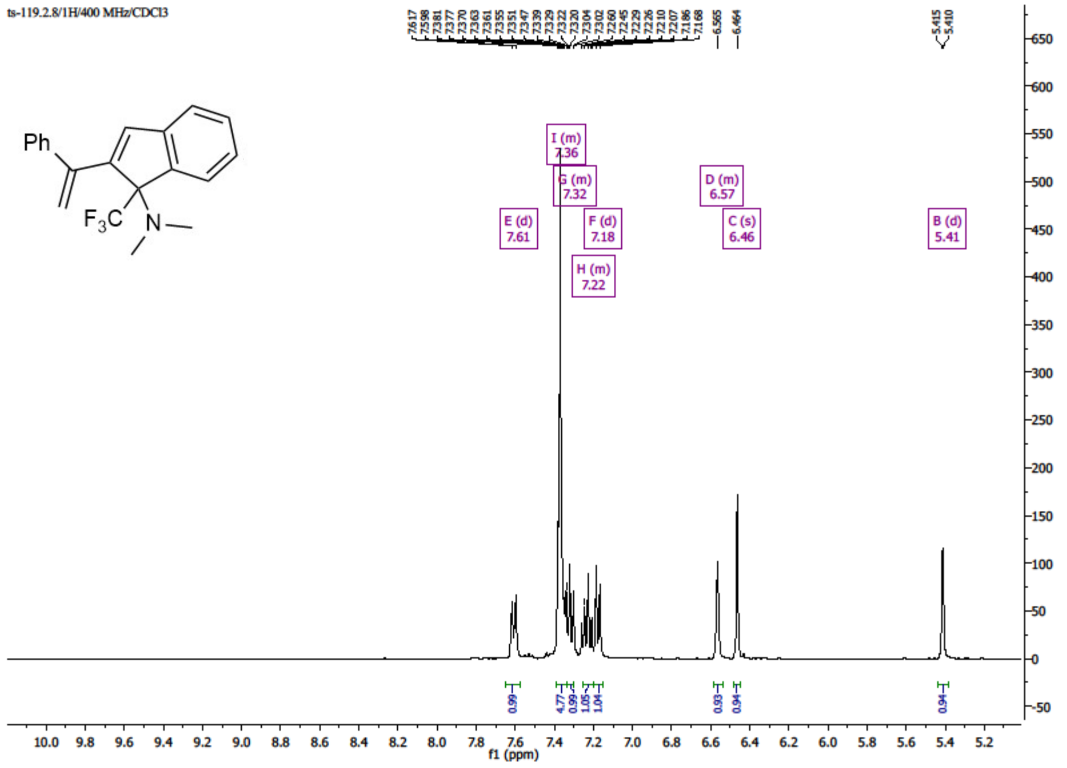

H,H-COSY NMR of **12a**, showing cross-peaks for the =CH^aH^b protons:

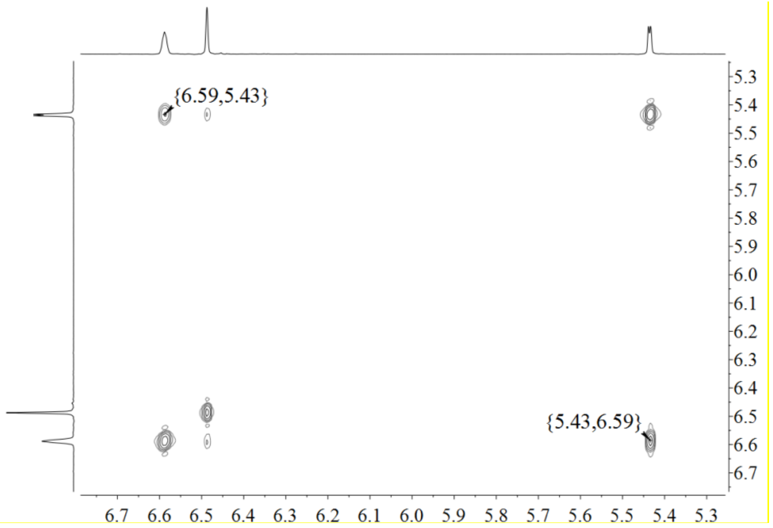

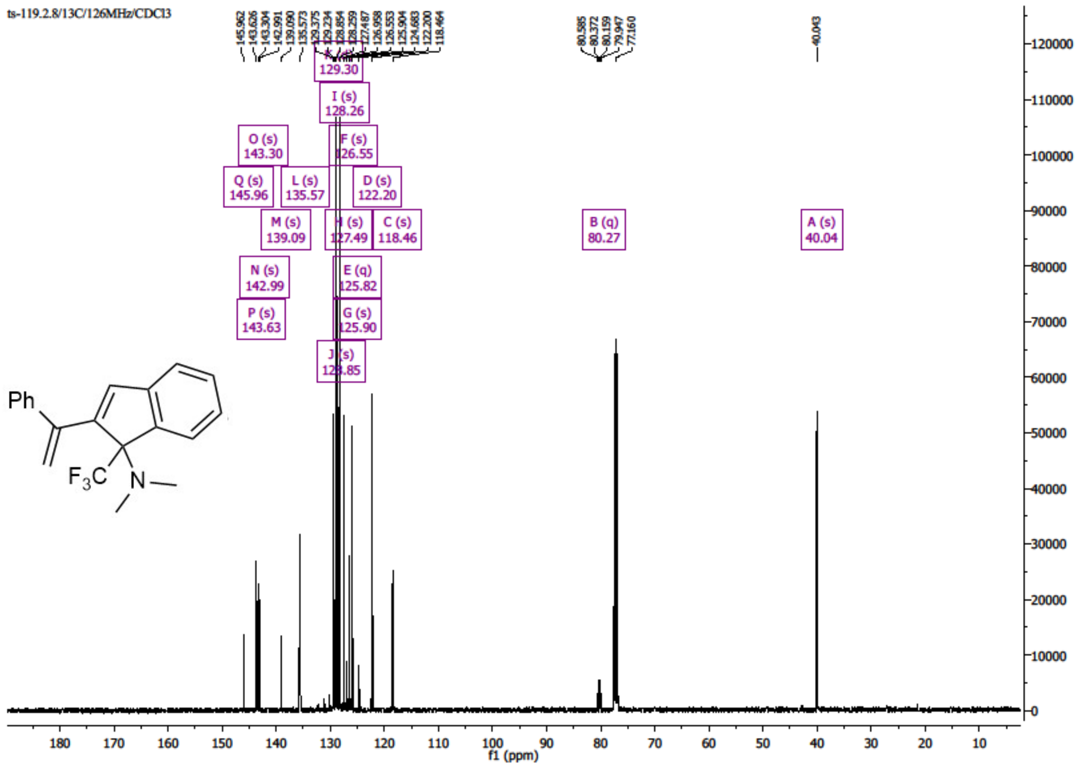
ts-119.2.8/13C/126MHz/CDCl3
145.962
143.626
143.304
142.991
139.090
135.573
129.375
129.234
128.854
128.259
127.487
126.958
126.553
125.904
124.683
122.200
118.464
80.585
80.372
80.159
79.947
77.160
40.043
Q (s) 145.96
P (s) 143.63
O (s) 143.30
N (s) 142.99
M (s) 139.09
L (s) 135.57
129.30
I (s) 128.26
F (s) 126.55
D (s) 122.20
C (s) 118.46
E (q) 125.82
G (s) 125.90
B (q) 80.27
A (s) 40.04
Ph
F3C
N
f1 (ppm)

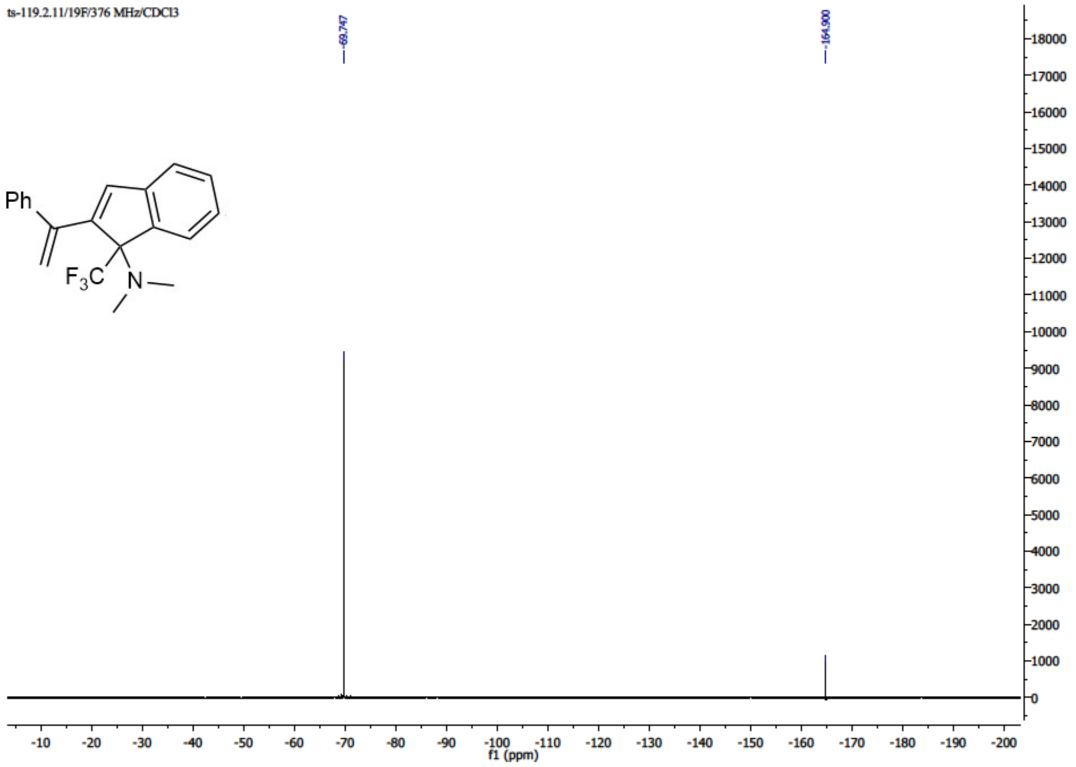
ts-119.2.11/19F/376 MHz/CDCl3
-69.747
-164.900
Ph
F3C
N
f1 (ppm)

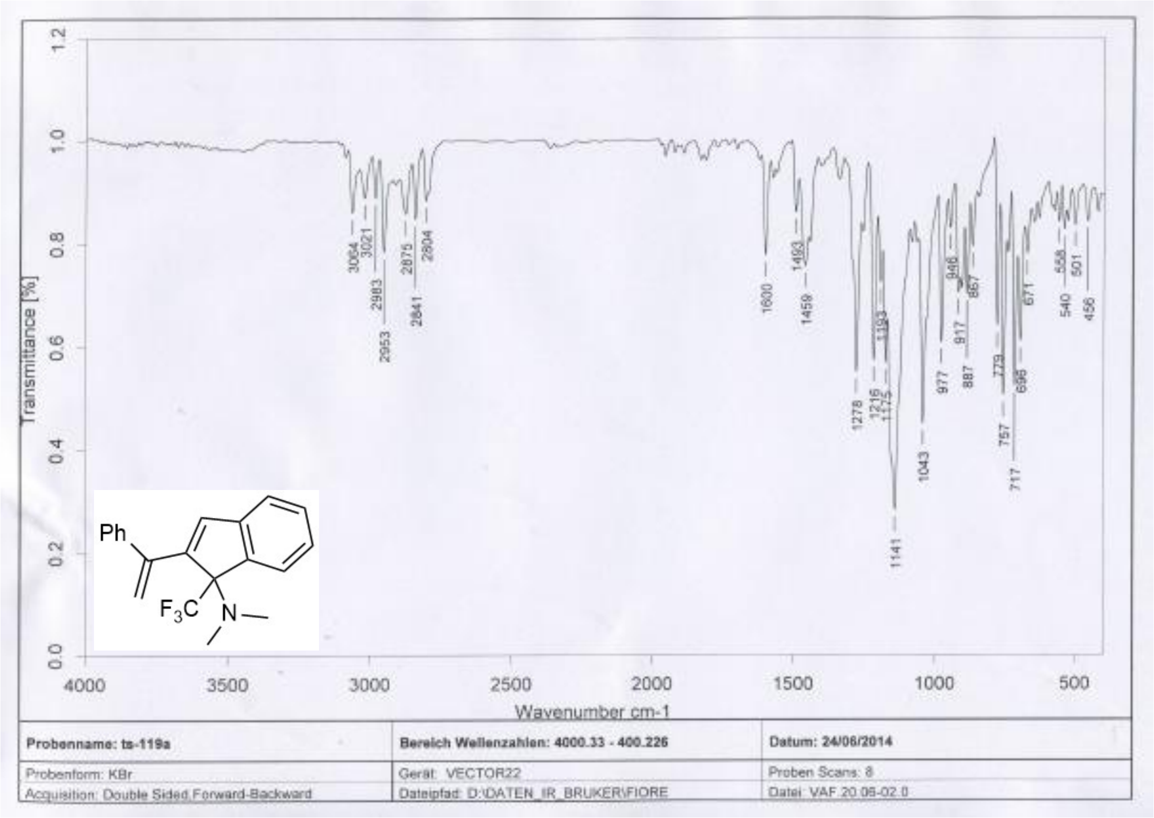
Transmittance [%]
Wavenumber cm-1
Ph
F_3C
N
Probenname: ts-119a
Bereich Wellenzahlen: 4000.33 - 400.226
Datum: 24/06/2014
Probenform: KBr
Gerät: VECTOR22
Proben Scans: 8
Acquisition: Double Sided,Forward-Backward
Dateipfad: D:\DATEN_IR_BRUKER\FIORE
Datei: VAF 20.06-02.0

N,N-Dimethyl-11-(trifluoromethyl)-11H-benzo[a]fluoren-11-amine (13a)

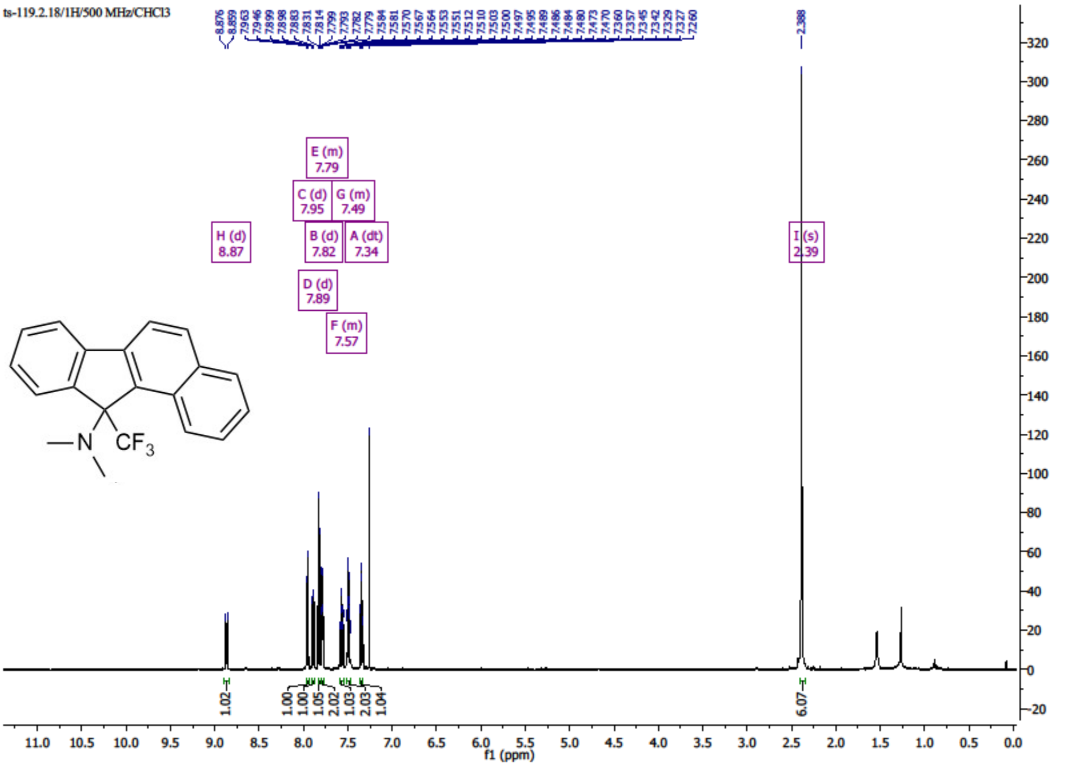

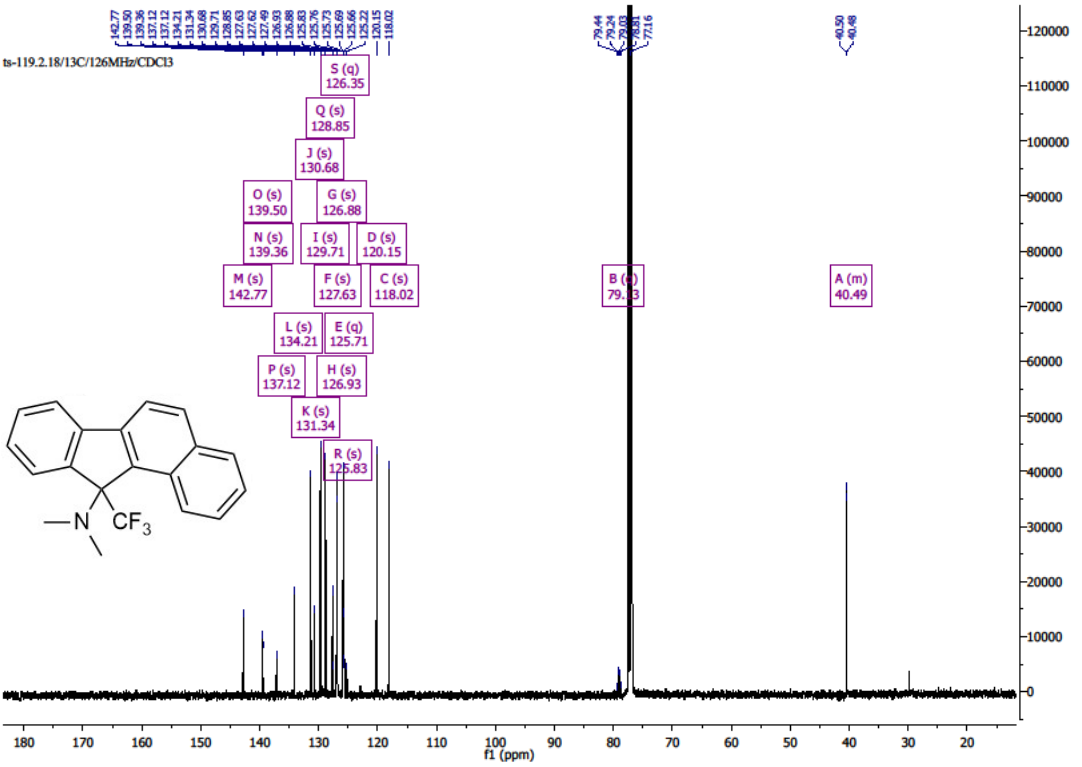

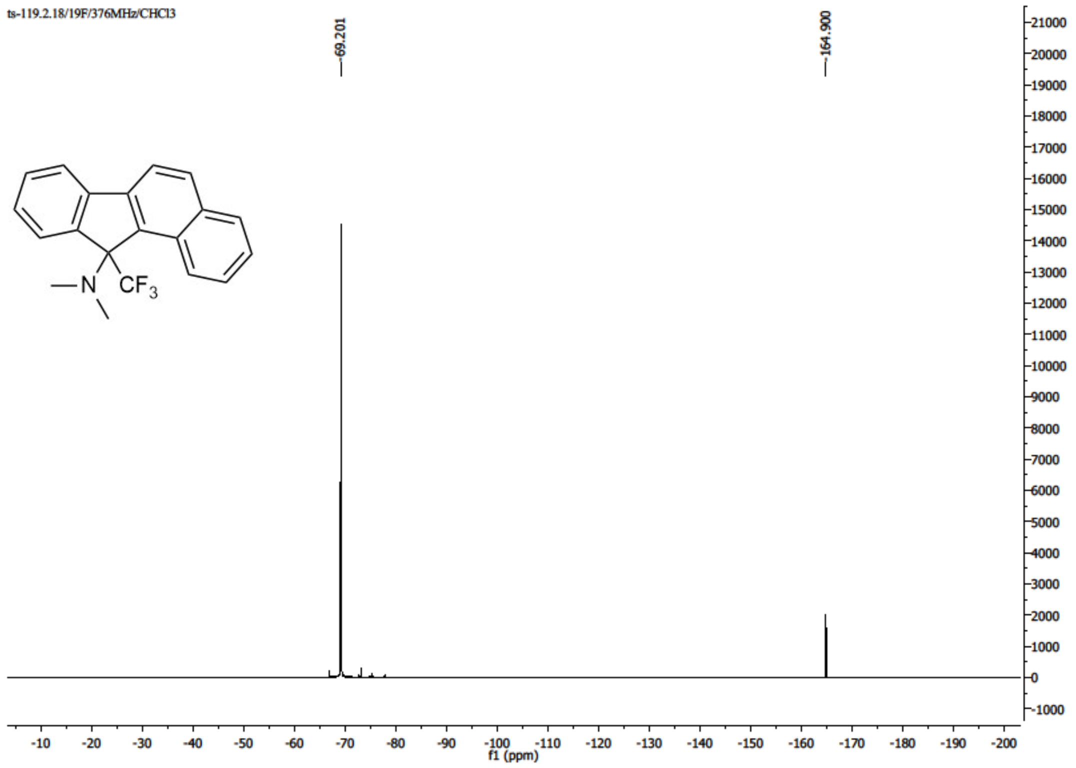
ts-119.2.18/19F/376MHz/CHCl3
—69.201
—164.900
N
CF3
f1 (ppm)

N,N,3-Trimethyl-2-(1-phenylvinyl)-1-(trifluoromethyl)-1H-inden-1-amine (12b)

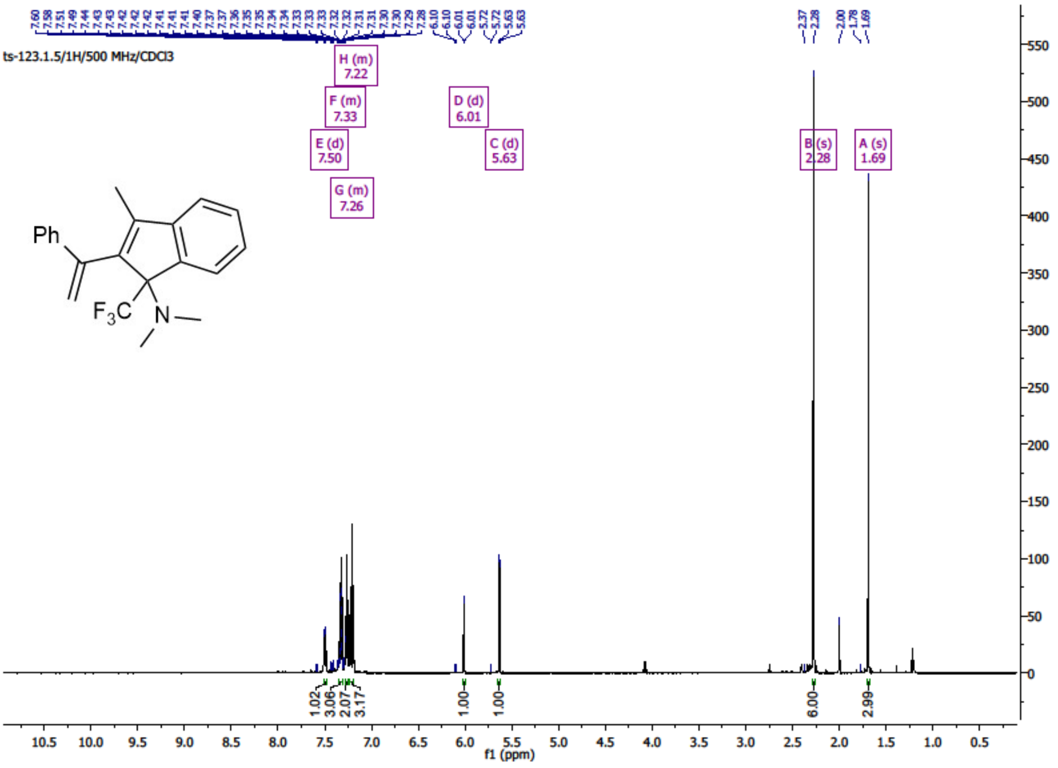

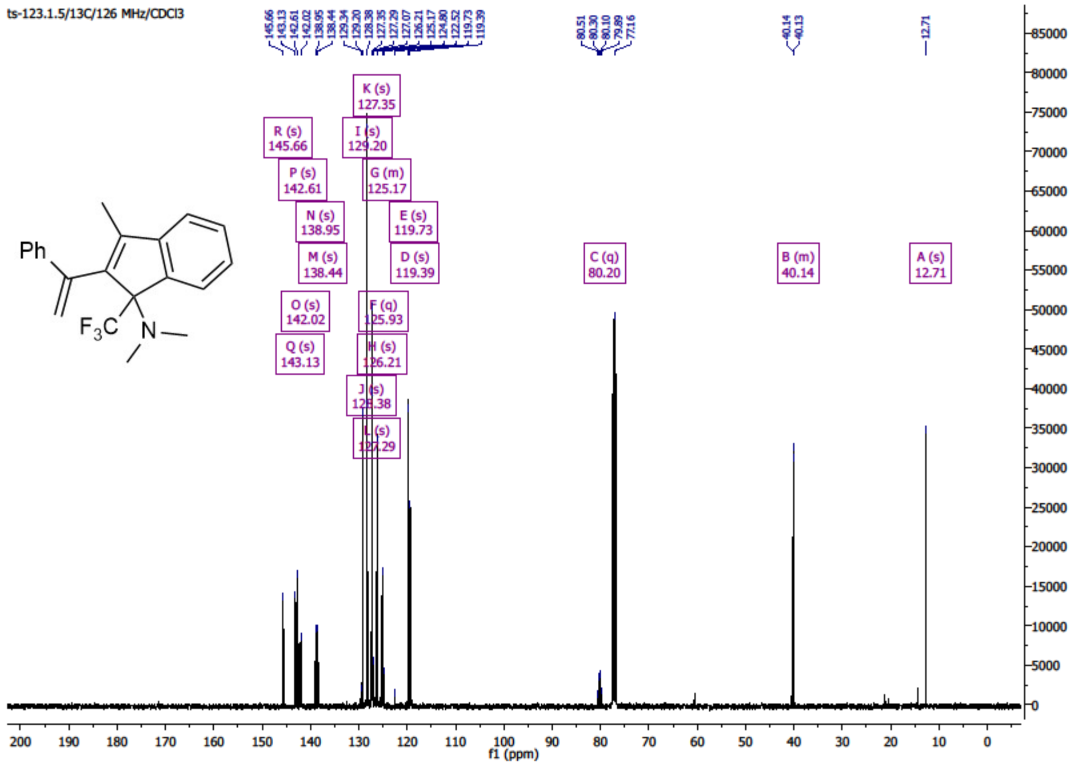

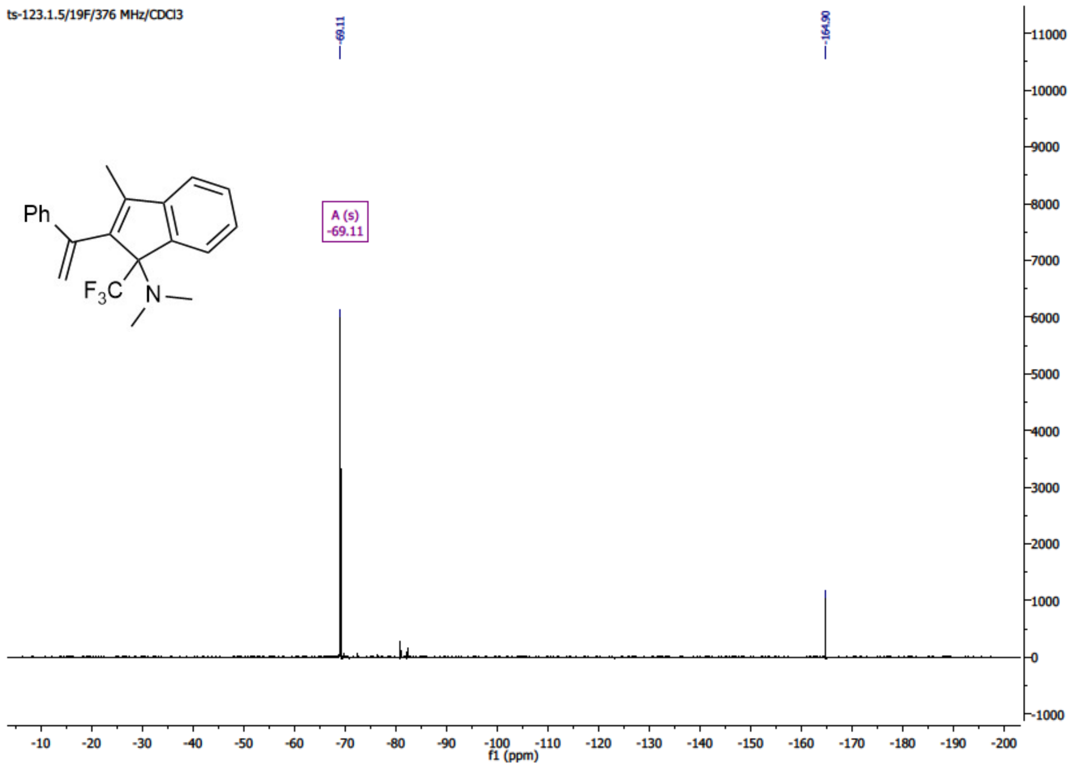

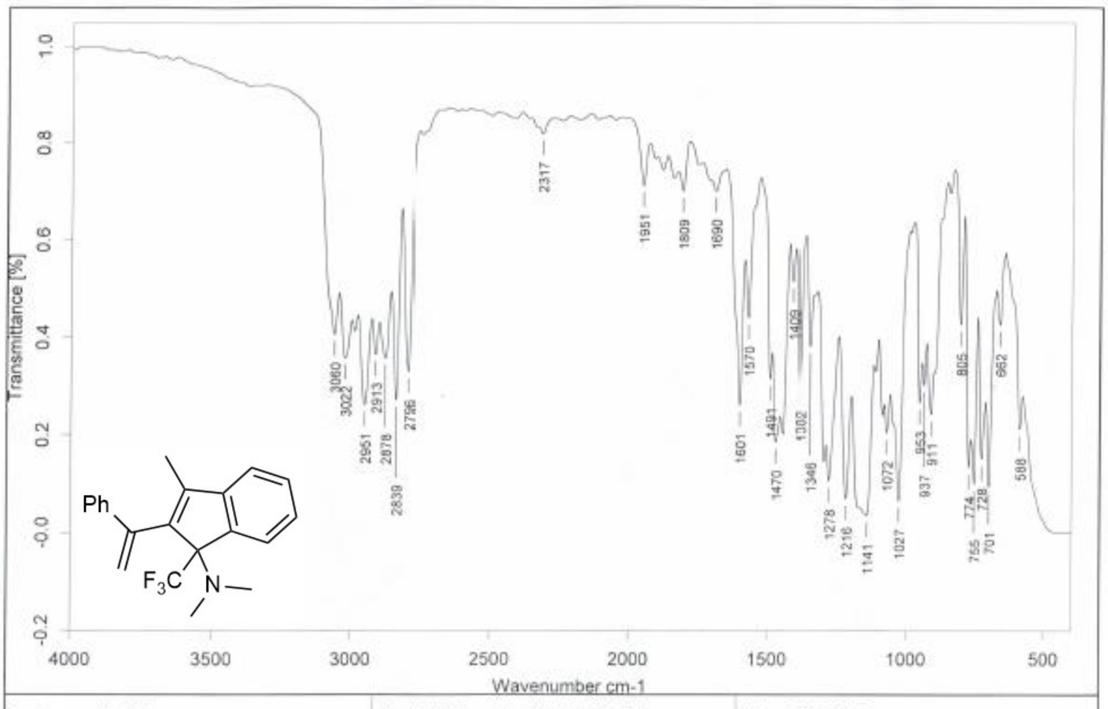

Probenname: ts-123	Bereich Wellenzahlen: 4000.33 - 400.226	Datum: 13/05/2014
Probenform: NaCl	Gerät: VECTOR22	Proben Scans: 10
Acquisition: Double Sided,Forward-Backward	Dateipfad: D:\DATEN_IR_BRUKER\KEIM	Datei: MH-34.1

***N*,*N*-Dimethyl-3-phenyl-2-(1-phenylvinyl)-1-(trifluoromethyl)-1*H*-inden-1-amine (12c)**

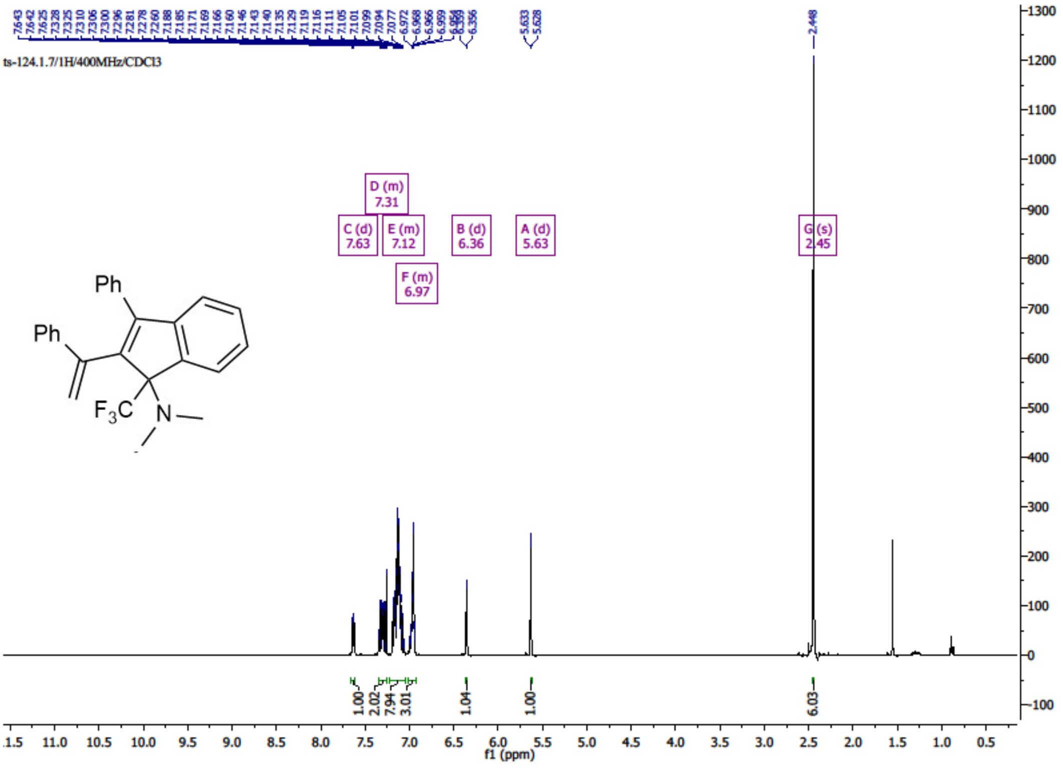

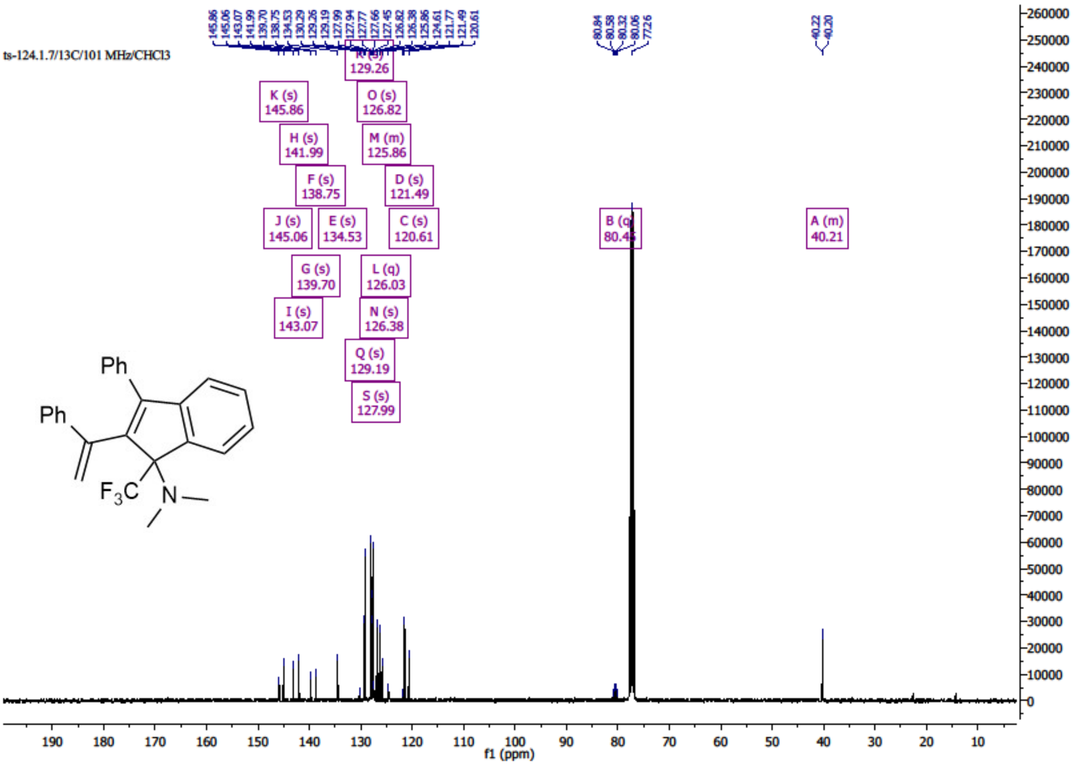

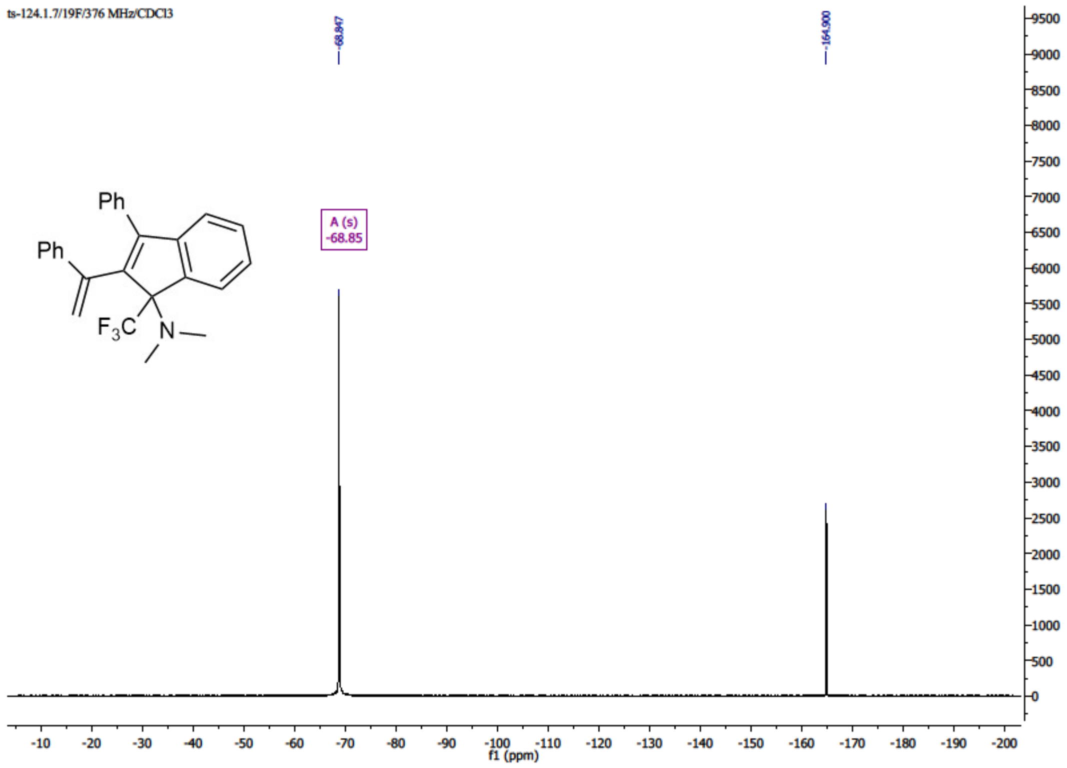
ts-124.1.7/19F/376 MHz/CDCl3
-68.847
-164.900
A (s)
-68.85
Ph
Ph
F3C
N
f1 (ppm)

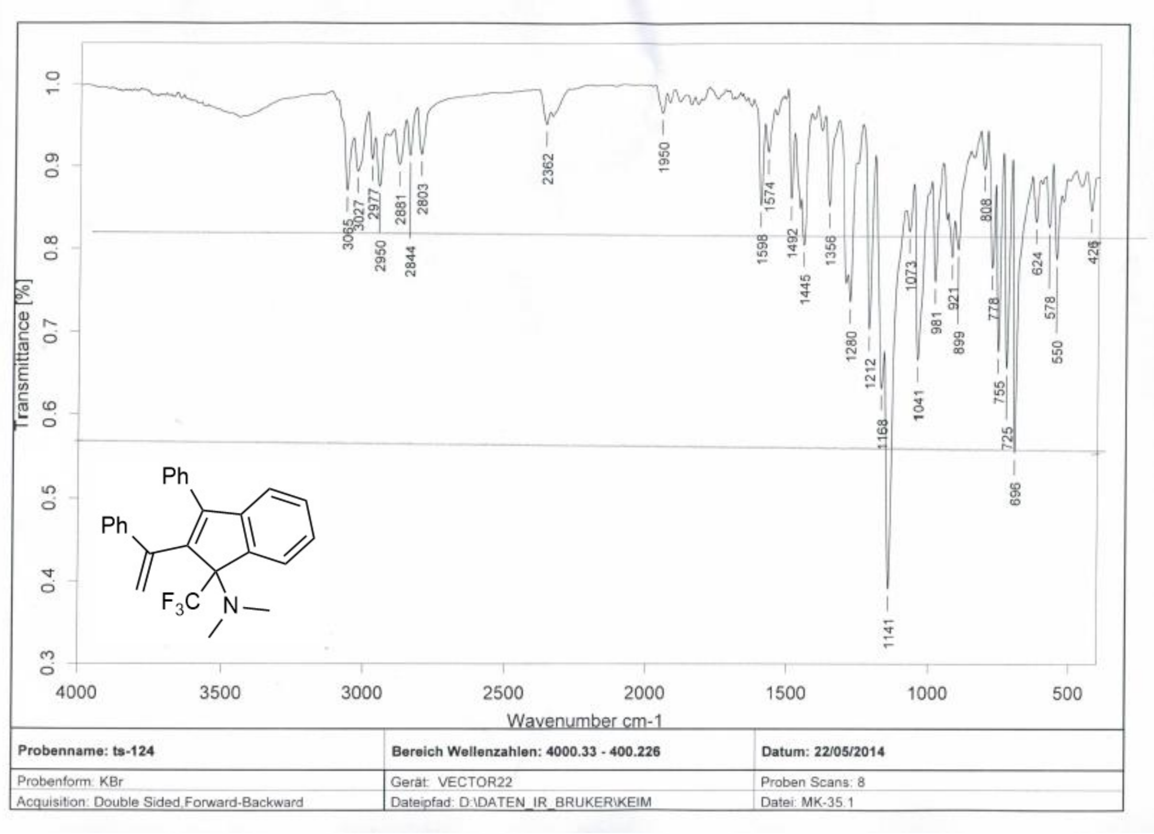
Transmittance [%]
Wavenumber cm-1
3065
3027
2977
2950
2881
2844
2803
2362
1950
1598
1574
1492
1445
1356
1280
1212
1168
1141
1073
1041
981
921
899
808
778
755
725
696
624
578
550
426
Ph
Ph
F3C
N
Probenname: ts-124
Bereich Wellenzahlen: 4000.33 - 400.226
Datum: 22/05/2014
Probenform: KBr
Gerät: VECTOR22
Proben Scans: 8
Acquisition: Double Sided,Forward-Backward
Dateipfad: D:\DATEN_IR_BRUKER\KEIM
Datei: MK-35.1

***N*,*N*-dimethyl-5-phenyl-11-(trifluoromethyl)-11*H*-benzo[*a*]fluoren-11-amine (13c)**

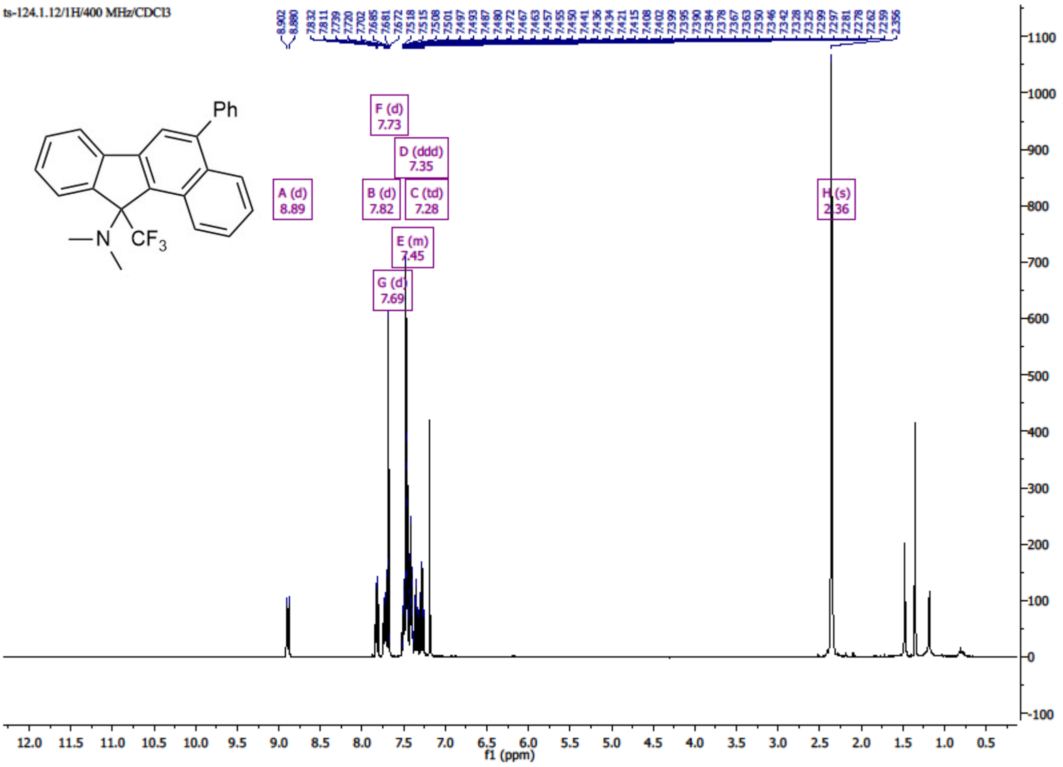

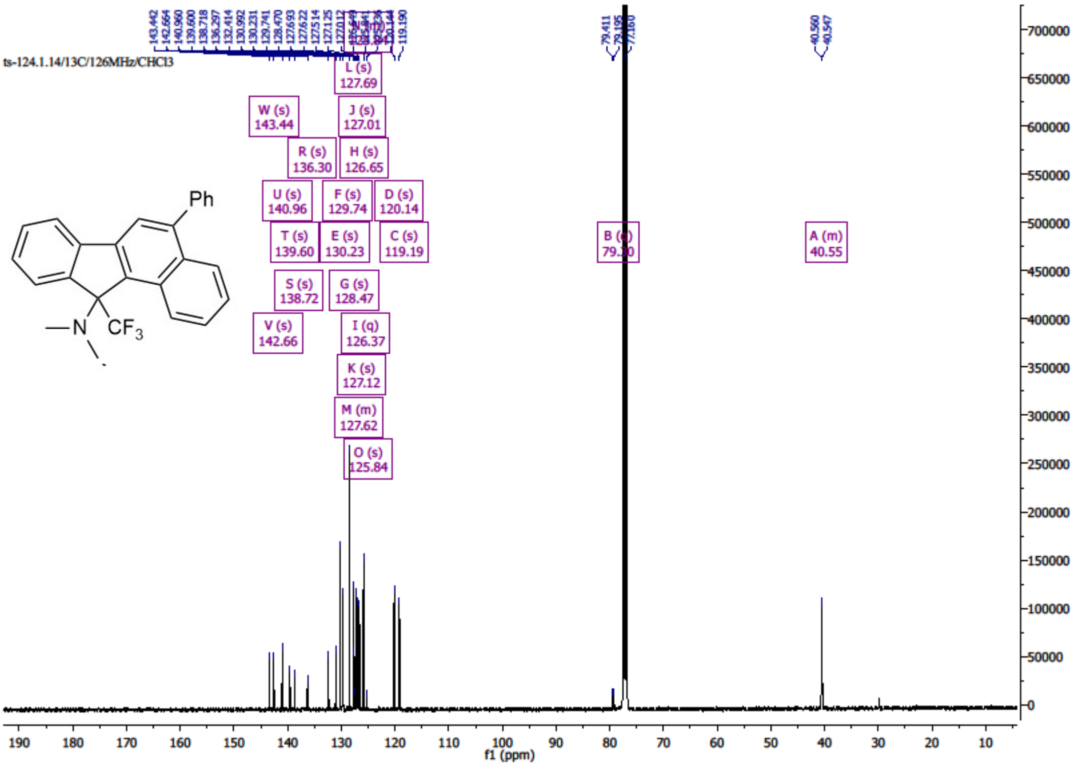

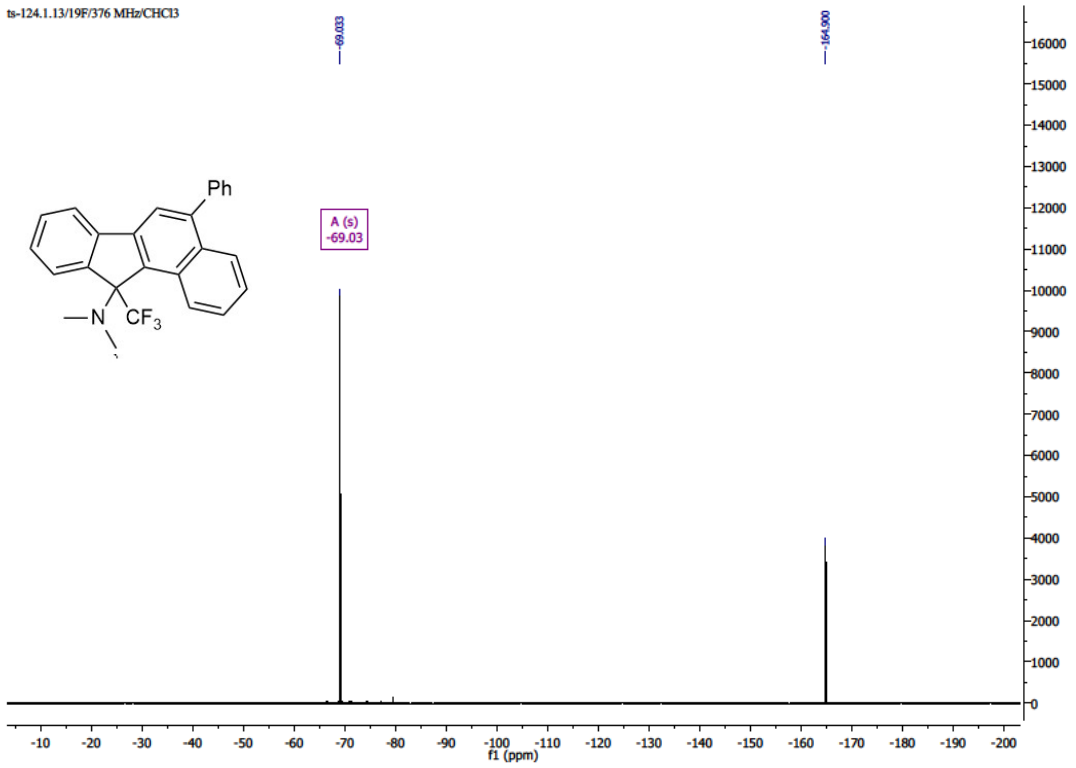

ts-124.1.13/19F/376 MHz/CHCl3
-69.033
-164.900
A (s)
-69.03
Ph
N
CF3
f1 (ppm)

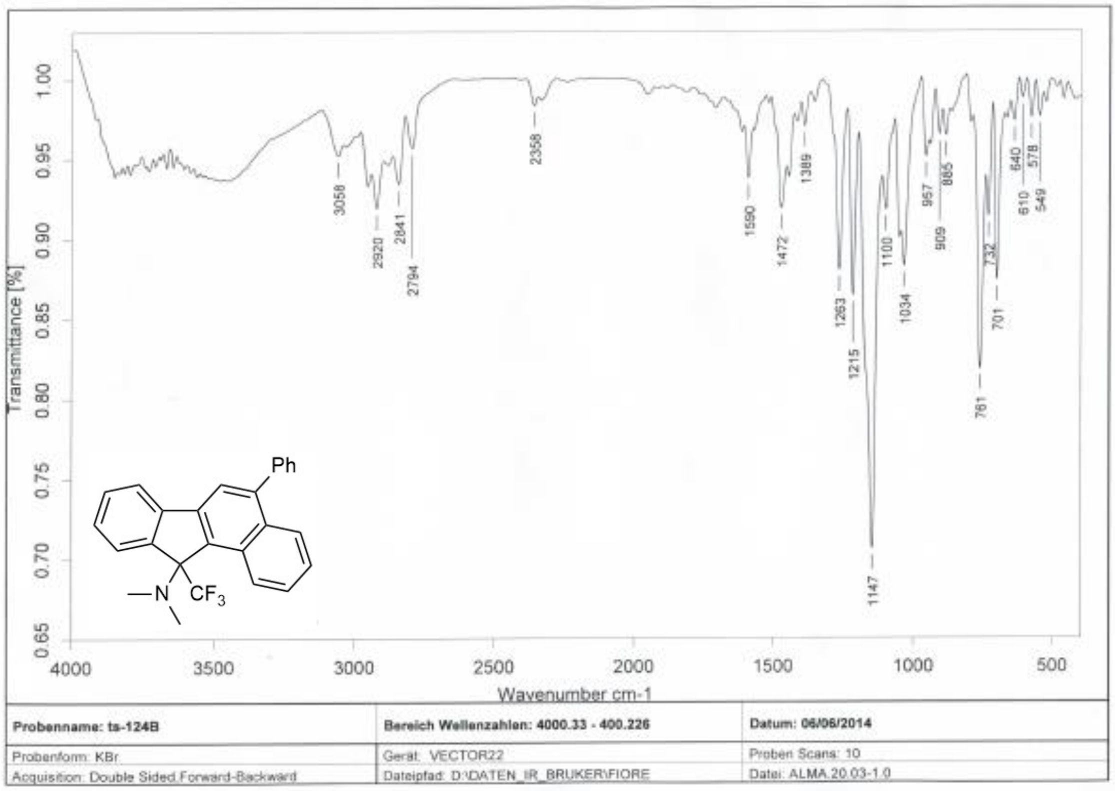

Transmittance [%]
Wavenumber cm-1
Ph
N
CF3
Probenname: ts-124B
Bereich Wellenzahlen: 4000.33 - 400.226
Datum: 06/06/2014
Probenform: KBr
Gerät: VECTOR22
Proben Scans: 10
Acquisition: Double Sided,Forward-Backward
Dateipfad: D:\DATEN_IR_BRUKER\FIORE
Datei: ALMA.20.03-1.0

(*E*)-*N*,*N*-Dimethyl-2-(1-phenylprop-1-en-1-yl)-1-(trifluoromethyl)-1*H*-inden-1-amine (12d)

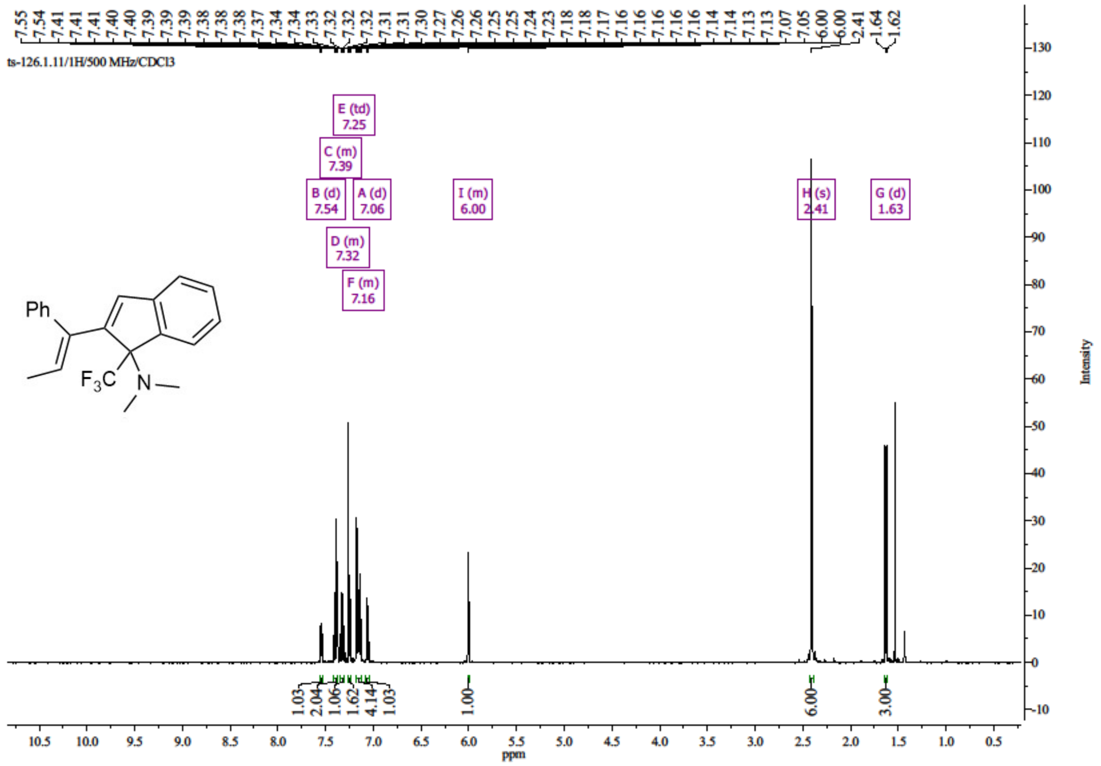

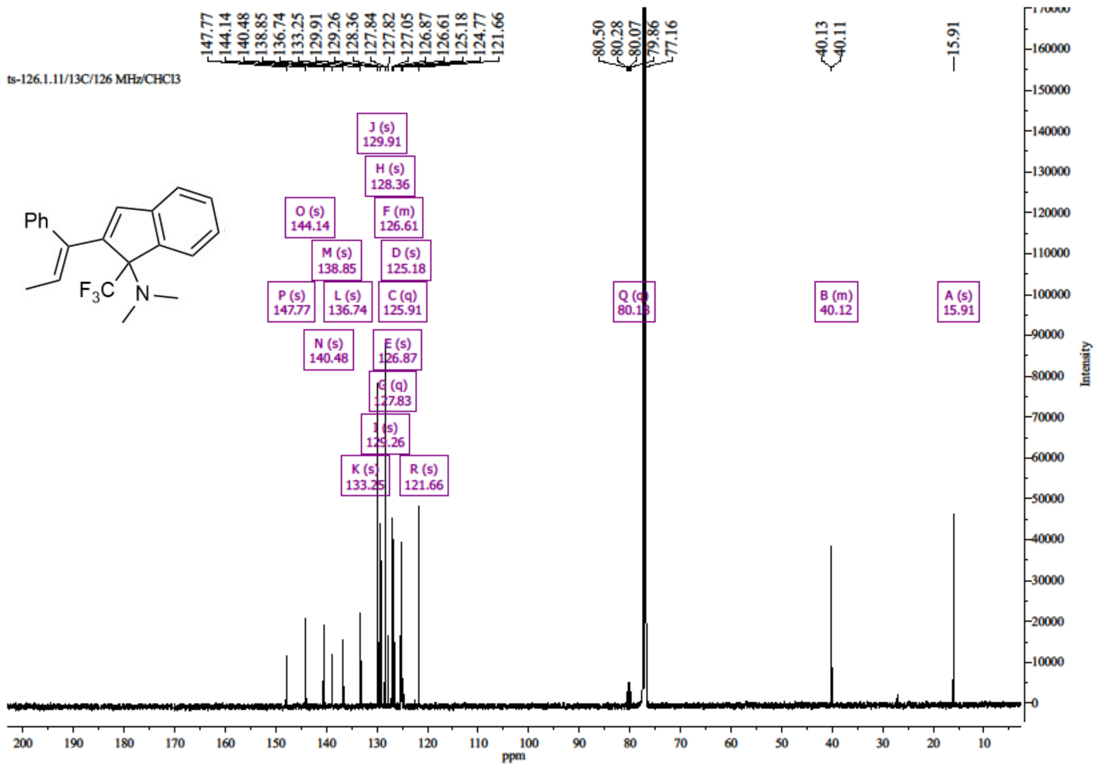

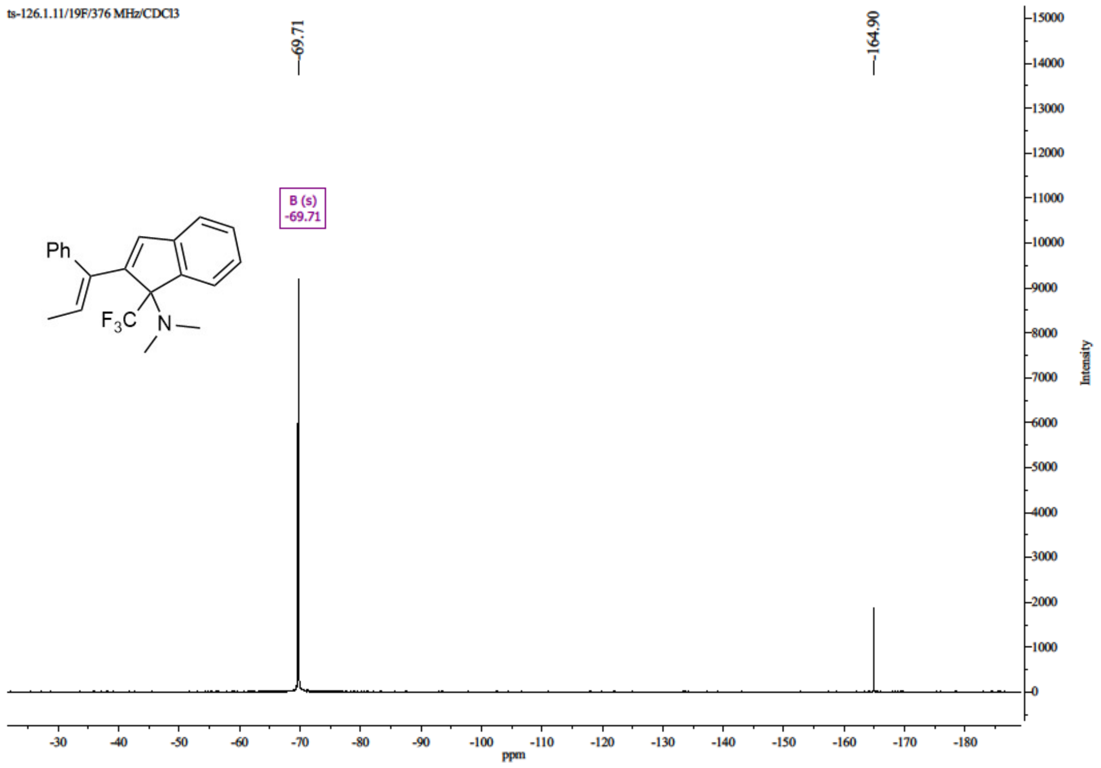
ts-126.1.11/19F/376 MHz/CDCl3
-69.71
-164.90
B (s)
-69.71
Ph
F3C
N
ppm
Intensity

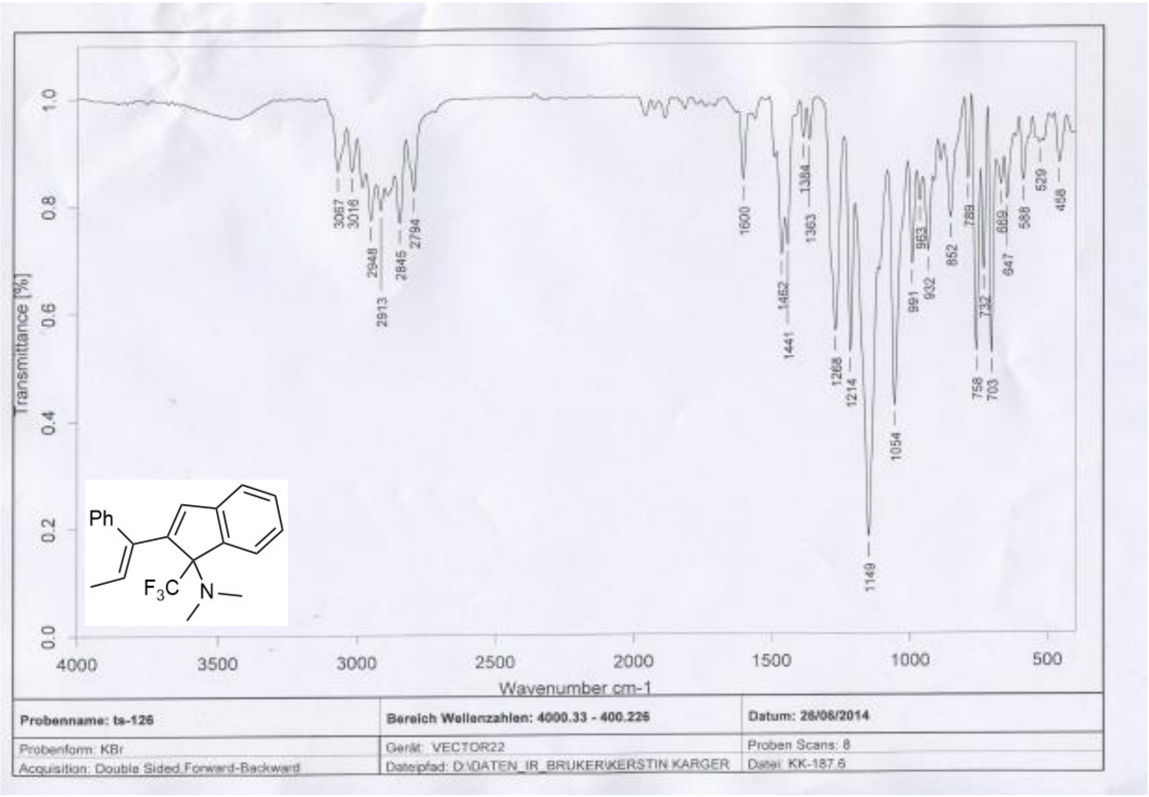
Transmittance [%]
Wavenumber cm-1
Ph
F3C
N
Probenname: ts-126
Bereich Wellenzahlen: 4000.33 - 400.226
Datum: 26/06/2014
Probenform: KBr
Gerät: VECTOR22
Proben Scans: 8
Acquisition: Double Sided Forward-Backward
Dateipfad: D:\DATEN_IR_BRUKER\KERSTIN KARGER
Datei: KK-187.6

2,2,2-Trifluoro-1-(2-phenyl-2a,3,4,8b-tetrahydrocyclobuta[*a*]naphthalen-1-yl)ethan-1-one 18

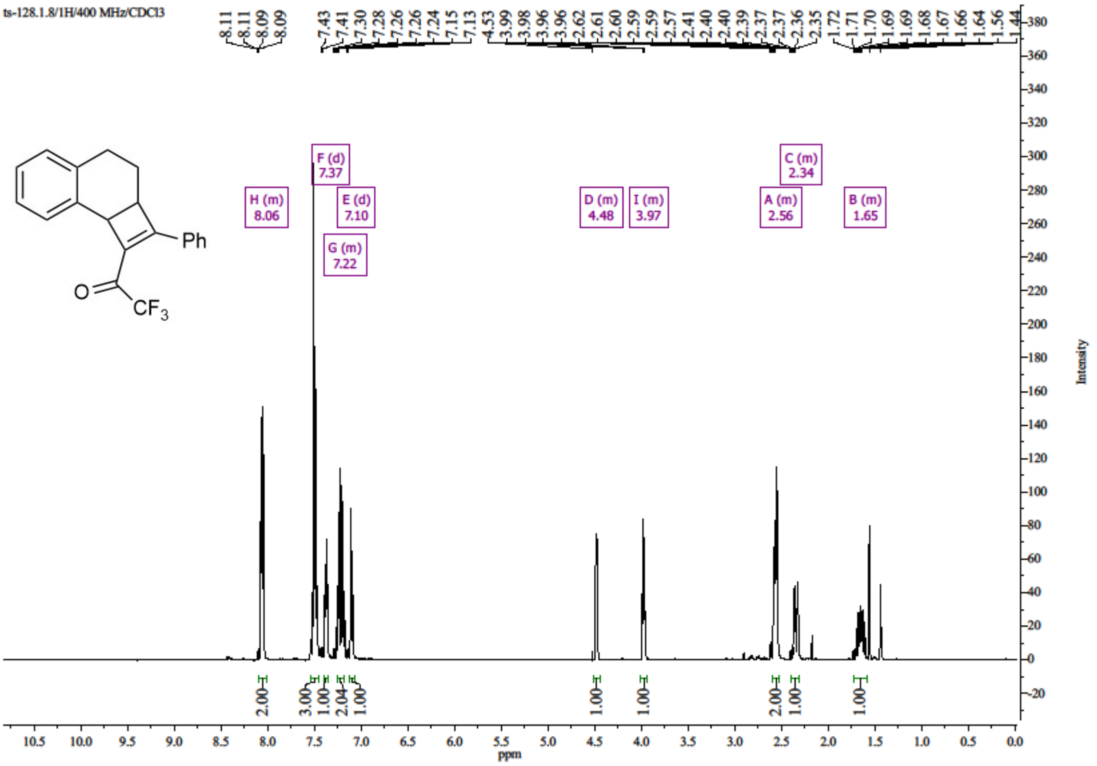

H,H-COSY NMR of **18**:

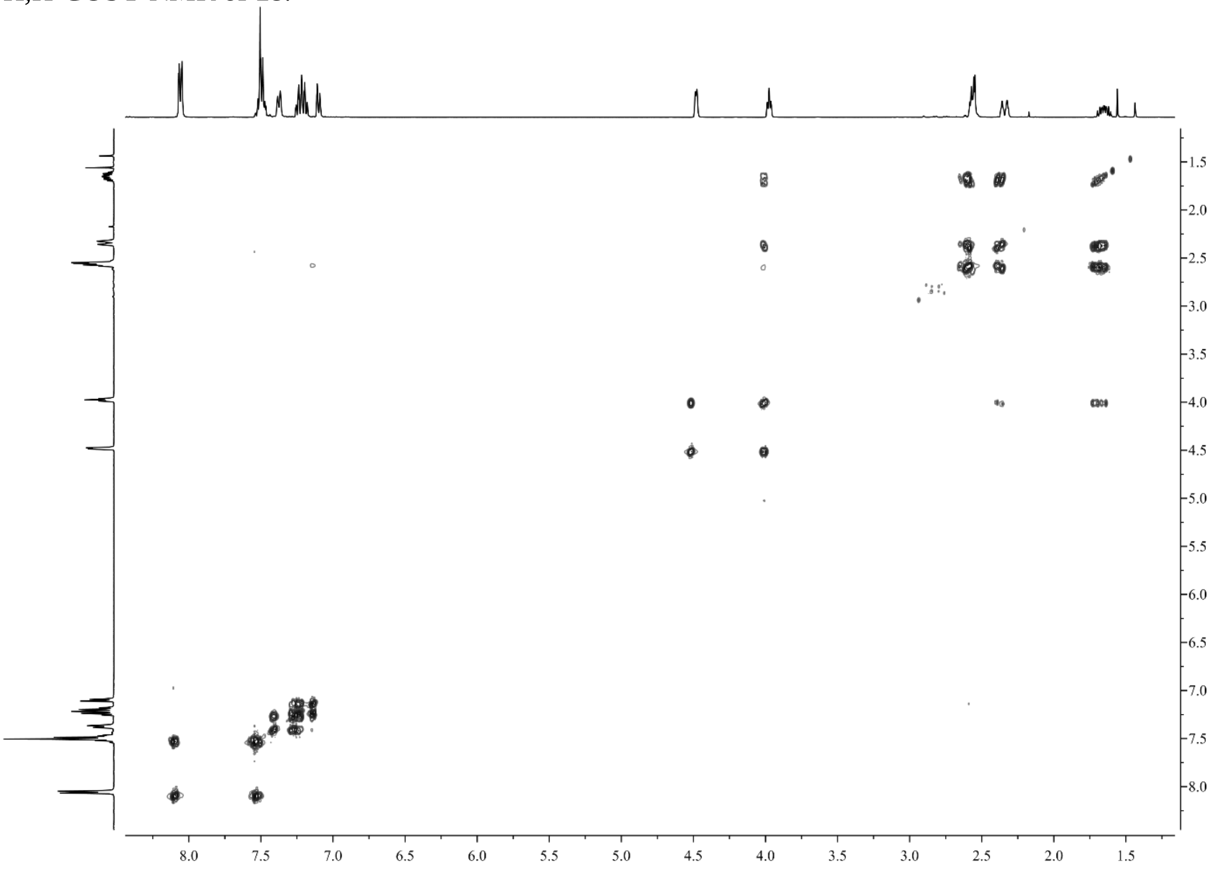

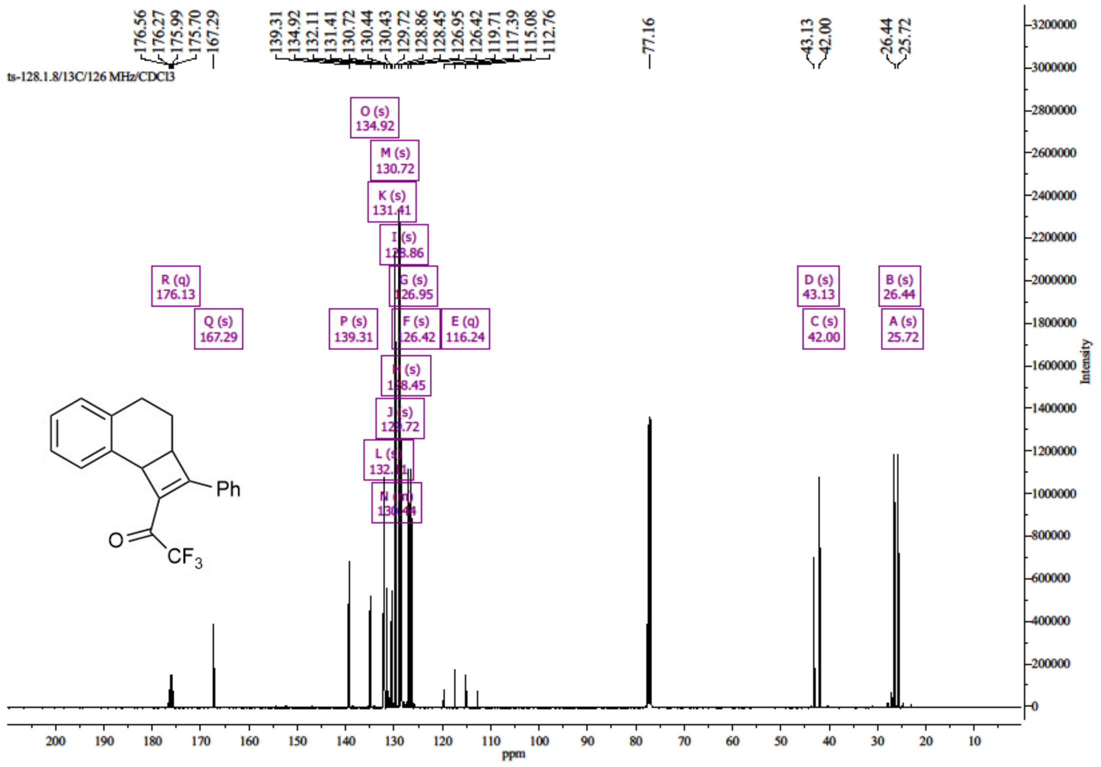
ts-128.1.8/13C/126 MHz/CDCl3
176.56
176.27
175.99
175.70
167.29
139.31
134.92
132.11
131.41
130.72
130.44
130.43
129.72
128.86
128.45
126.95
126.42
119.71
117.39
115.08
112.76
77.16
43.13
42.00
26.44
25.72
R (q) 176.13
Q (s) 167.29
P (s) 139.31
O (s) 134.92
M (s) 130.72
K (s) 131.41
G (s) 126.95
F (s) 126.42
E (q) 116.24
D (s) 43.13
C (s) 42.00
B (s) 26.44
A (s) 25.72
Ph
O
CF3
ppm
Intensity

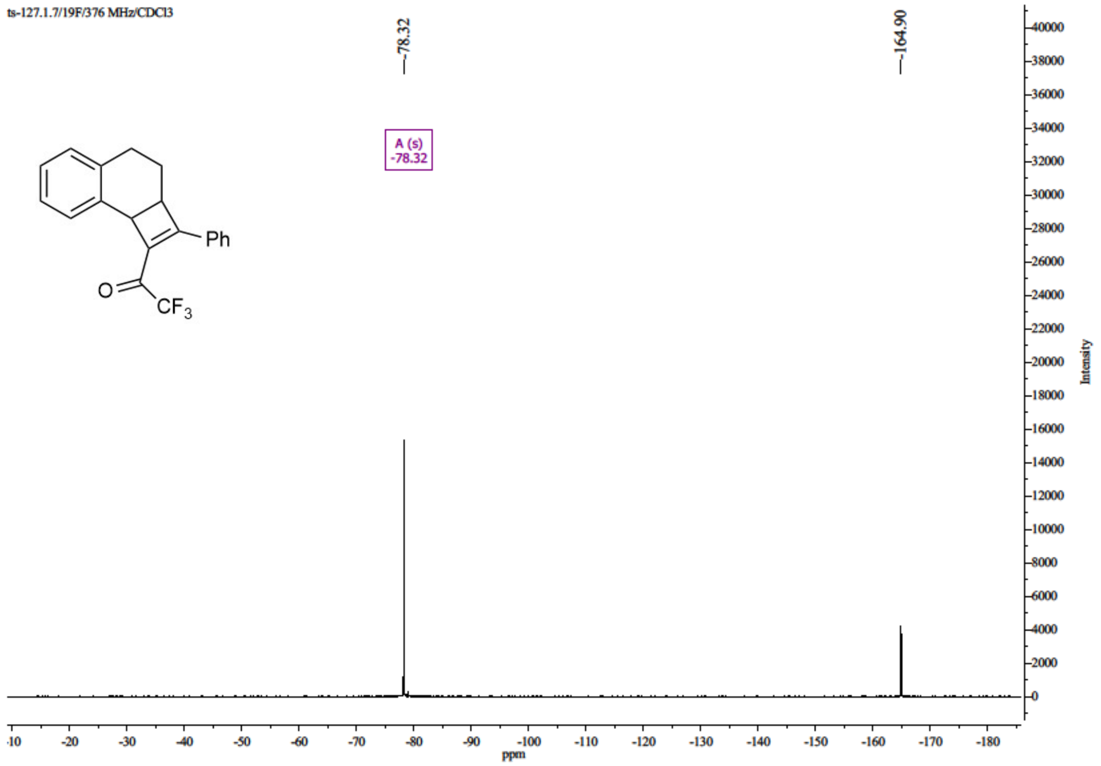
ts-127.1.7/19F/376 MHz/CDCl3
-78.32
-164.90
A (s) -78.32
Ph
O
CF3
ppm
Intensity

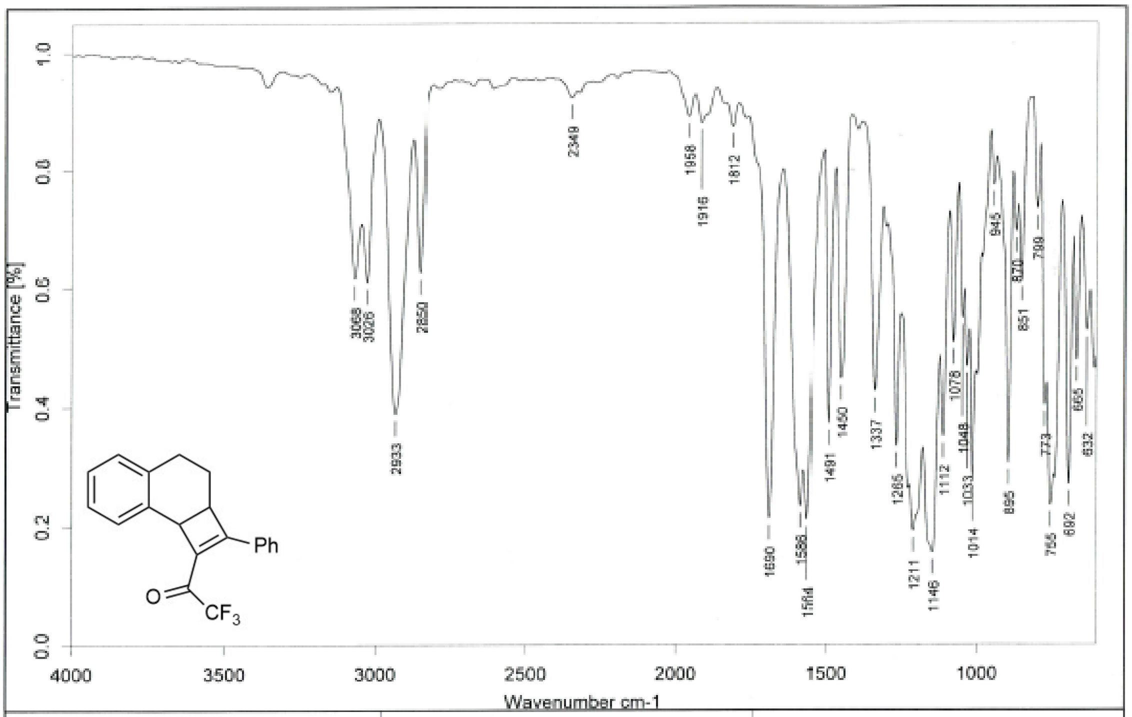

Probenname: ts-129	Bereich Wellenzahlen: 4000.33 - 400.226	Datum: 21/07/2014
Probenform: NaCl	Gerät: VECTOR22	Proben Scans: 8
Acquisition: Double Sided,Forward-Backward	Dateipfad: D:\DATEN_IR_BRUKER\OC3\CHRISTIANE	Datei: CSE-R27-180714.2

(*Z*)-2-(4-Bromophenyl)-*N*,*N*-dimethyl-1-phenyl-2-(3-phenyl-2-(trifluoromethyl)cyclopenta-2,4-dien-1-yliden)ethan-1-amine (19)

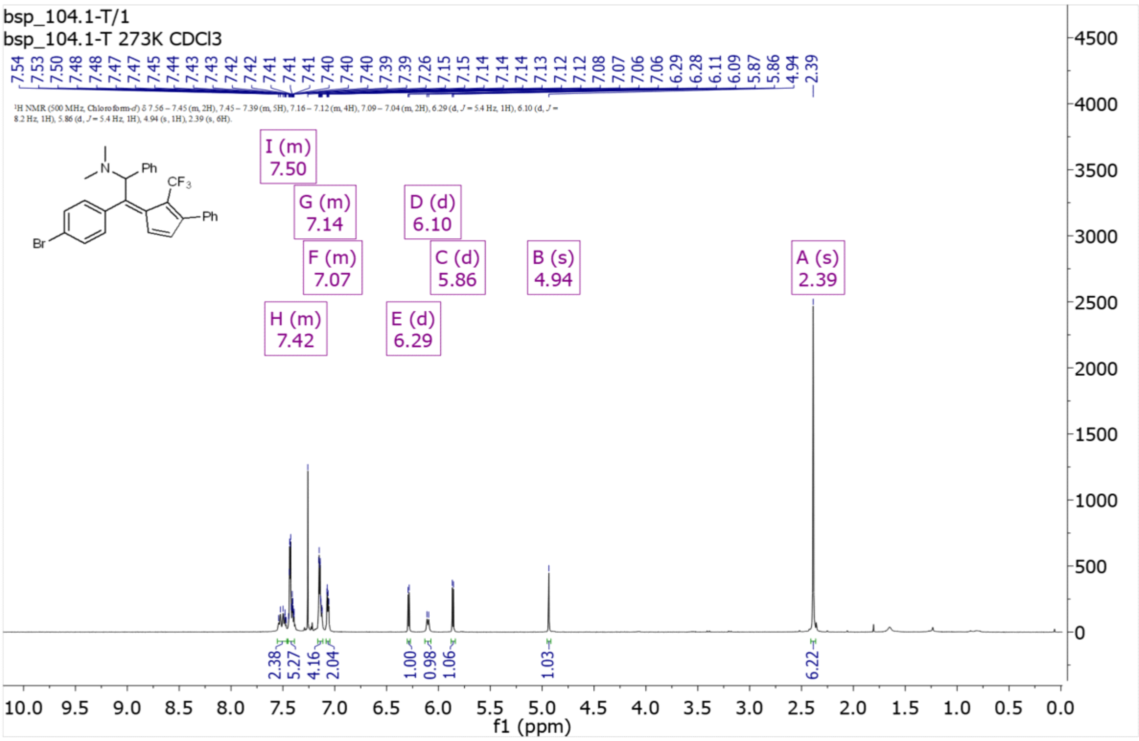

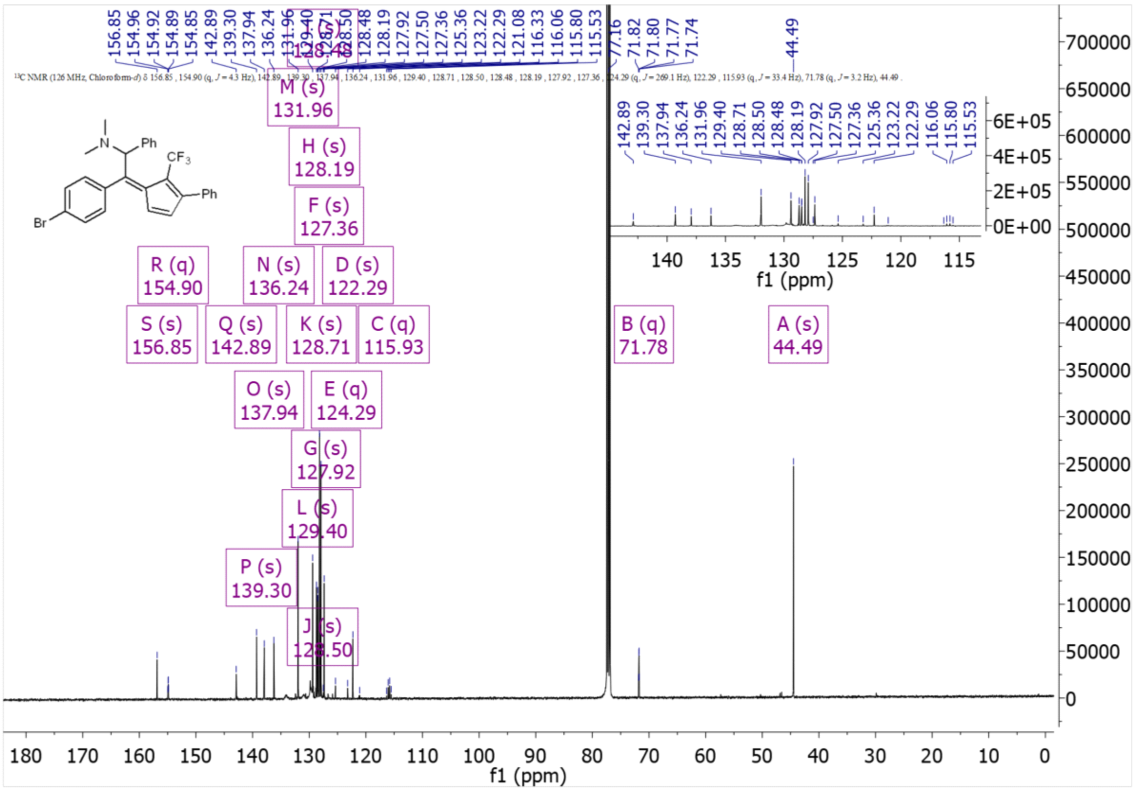

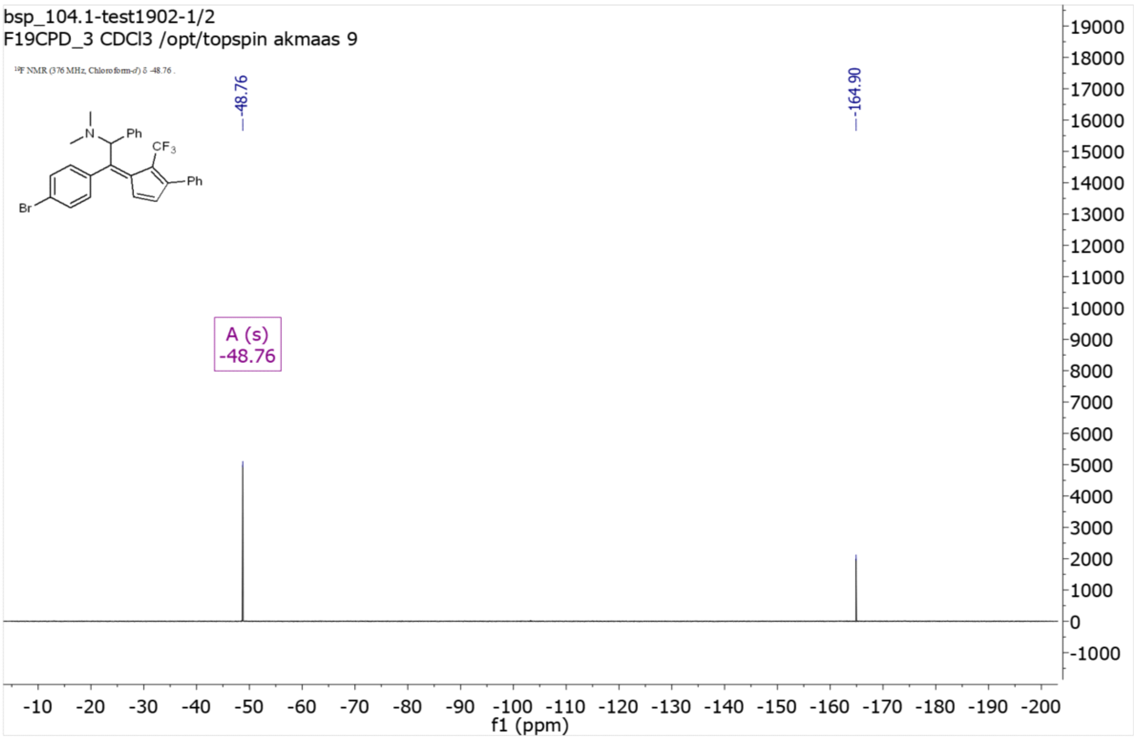
bsp_104.1-test1902-1/2
F19CPD_3 CDCl3 /opt/topspin akmaas 9
19F NMR (376 MHz, Chloroform-d) δ -48.76 .
-48.76
-164.90
N
Ph
CF3
Ph
Br
A (s)
-48.76
f1 (ppm)

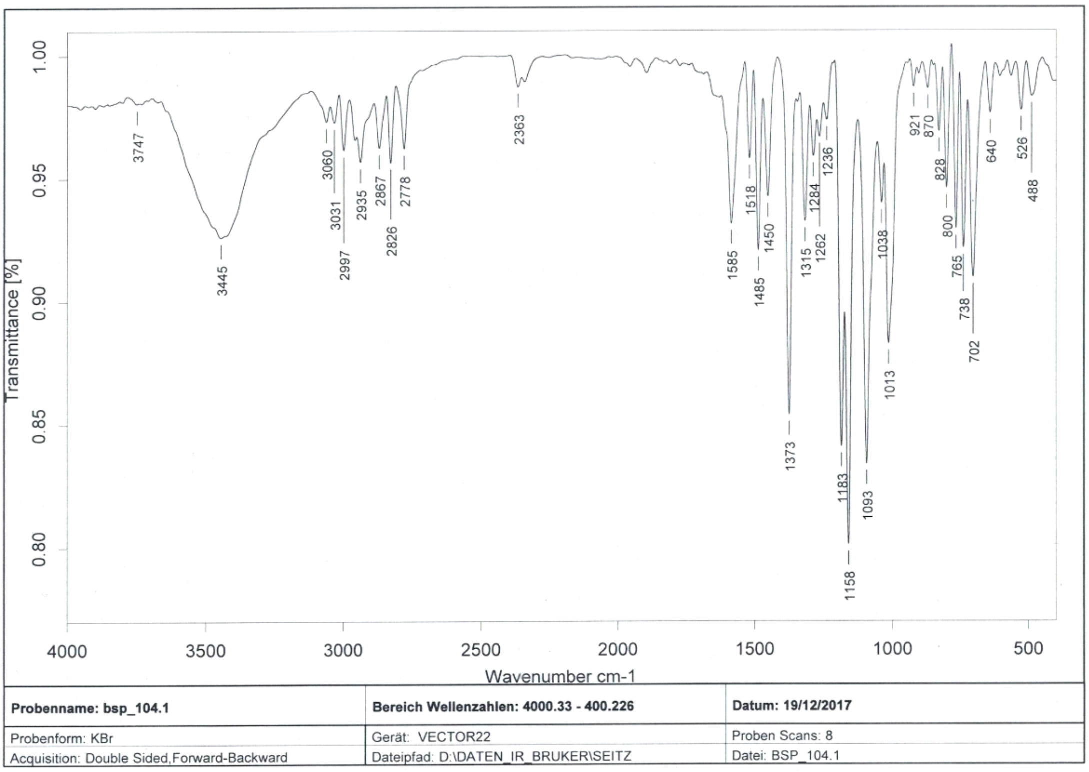
Transmittance [%]
Wavenumber cm-1
3747
3445
3060
3031
2997
2935
2867
2826
2778
2363
1585
1518
1485
1450
1373
1315
1284
1262
1236
1183
1158
1093
1038
1013
921
870
828
800
765
738
702
640
526
488
Probenname: bsp_104.1
Bereich Wellenzahlen: 4000.33 - 400.226
Datum: 19/12/2017
Probenform: KBr
Gerät: VECTOR22
Proben Scans: 8
Acquisition: Double Sided,Forward-Backward
Dateipfad: D:\DATEN_IR_BRUKER\SEITZ
Datei: BSP_104.1

3. References

[1] T. Schneider, M. Keim, M. Schiwek, G. Maas, *J. Fluorine Chem.* **2020**, *235*, 109567; DOI: 10.1016/j.fluchem.2020.109567.
