# Supplementary material for: Reactions of 3-aryl-1-(trifluoromethyl)prop-2-yn-1-iminium salts with 1,3-dienes and styrenes
Source: Beilstein J Org Chem. 2020 Aug 24;16:2064–72. doi: 10.3762/bjoc.16.173 (PMC7476585; doi:10.3762/bjoc.16.173)
Supplement: File 2 — Crystal and structure refinement data for compounds 11, 12c, 12d and 19. [file Beilstein_J_Org_Chem-16-2064-s002.pdf]

BEILSTEIN JOURNAL OF ORGANIC CHEMISTRY

Supporting Information

for

Reactions of 3-aryl-1-(trifluoromethyl)prop-2-yn-1-iminium salts with 1,3-dienes and styrenes

Thomas Schneider, Michael Keim, Bianca Seitz and Gerhard Maas

Beilstein J. Org. Chem. **2020,** *16,* 2064–2072. doi:10.3762/bjoc.16.173

Crystal and structure refinement data for compounds 11, 12c, 12d and 19

License and Terms: This is a supporting information file under the terms of the Creative Commons Attribution License (https://creativecommons.org/licenses/by/4.0). Please note that the reuse, redistribution and reproduction in particular requires that the authors and source are credited.
The license is subject to the *Beilstein Journal of Organic Chemistry* terms and conditions: (https://www.beilstein-journals.org/bjoc)

General Information

The data collection on single crystals was performed on an Oxford Diffraction Rigaku instrument (SuperNova, Dual Source, Atlas CCD, Mo K_α or Cu K_α radiation). For structure solution and refinement, the following programs were used: SHELXS97 [1] SHELXL-2014 and SHELXL-2018/3 [2]. Molecule plot: ORTEP-3 [3]. CCDC contains the Supplementary crystallographic data for this paper have been deposited (CCDC numbers are found in the Tables. These data can be obtained free of charge from The Cambridge Crystallographic Data Centre via www.ccdc.cam.ac.uk/getstructures.

[1] G. M. Sheldrick, A short history of SHELX, Acta Crystallogr. A64 (2008) 112–122.

[2] G. M. Sheldrick, Crystal structure refinement with SHELXL, Acta Crystallogr. C71 (2015) 3–8.

[3] L. Farrugia, WinGX and ORTEP for Windows: an update, ORTEP-3 for Windows, J. Appl. Crystallogr. 45 (2012) 849–854.

Table 1. Crystal data and structure refinement for *N*,*N*-Dimethyl-11-(trifluoromethyl)-10,11-dihydro-5*H*-5,10-[1,2]benzenobenzo[*b*]fluoren-11-amine (**11**)

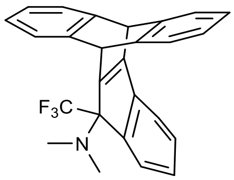

Crystallization from *n*-pentane.

Special details: The unit cell contains two symmetry-independent molecules in the asymmetric unit.

Identification code	TS317	
Empirical formula	C26 H20 F3 N	
Formula weight	403.43	
Temperature	150(2) K	
Wavelength	0.71073 Å	
Crystal system	orthorhombic	
Space group	*P n a* 2_1	
Unit cell dimensions	*a* = 15.2843(4) Å	α = 90°.
	b = 7.9425(2) Å	β = 90°.
	c = 32.9795(11) Å	γ = 90°.
Volume	4003.6(2) $Å^3$	
Z	8	
Density (calculated)	1.339 Mg/m^3	
Absorption coefficient	0.097 mm^{-1}	
F(000)	1680	
Crystal size	0.22 x 0.15 x 0.06 mm^3	
Theta range for data collection	2.890 to 26.369°.	
Index ranges	-19<=*h*<=16, -9<=*k*<=5, -41<=*l*<=35	
Reflections collected	14620	
Independent reflections	6348 [*R*(int) = 0.0298]	
Completeness to theta = 25.242°	99.9 %	
Refinement method	Full-matrix least-squares on F^2	
Data / restraints / parameters	6348 / 1 / 545	
Goodness-of-fit on F^2	1.060	
Final *R* indices [*I*>2sigma(*I*)]	*R*1 = 0.0421, *wR*2 = 0.0964	
R indices (all data)	*R*1 = 0.0521, *wR*2 = 0.1023	
Absolute structure parameter	-0.1(4) (Flack test)	
Extinction coefficient	n/a	
Largest diff. peak and hole	0.207 and -0.207 e.$Å^{-3}$	
CCDC number	2003232	

Table 2. Crystal data and structure refinement for
N,*N*,3-Trimethyl-2-(1-phenylvinyl)-1-(trifluoromethyl)-1*H*-inden-1-amine (**12c**)

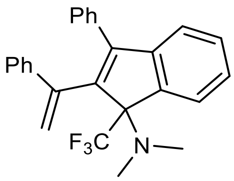

Crystallization from *n*-pentane.
Special details: The positions of the olefinic $=CH_2$ group were taken from a difference Fourier map and refined with isotropic temperature factors. The unit cell contains two symmetry-independent molecules in the asymmetric unit.

Identification code	ts266	
Empirical formula	C26 H22 F3 N	
Formula weight	405.45	
Temperature	180(2) K	
Wavelength	0.71073 Å	
Crystal system	monoclinic	
Space group	P 1 2_1/n 1	
Unit cell dimensions	a = 8.5110(3) Å	α = 90°.
	b = 12.6247(5) Å	β = 94.363(3)°.
	c = 19.6928(6) Å	γ = 90°.
Volume	2109.83(13) $Å^3$	
Z	4	
Density (calculated)	1.276 Mg/m^3	
Absorption coefficient	0.092 mm^{-1}	
F(000)	848	
Crystal size	0.26 x 0.16 x 0.16 mm^3	
Theta range for data collection	2.89 to 26.37°.	
Index ranges	$-10<=h<=10$, $-15<=k<=15$, $-24<=l<=24$	
Reflections collected	12136	
Independent reflections	4304 [R(int) = 0.0306]	
Completeness to theta = 26.37°	99.9 %	
Absorption correction	Semi-empirical from equivalents	
Max. and min. transmission	1.00000 and 0.83558	
Refinement method	Full-matrix least-squares on F^2	
Data / restraints / parameters	4304 / 0 / 281	
Goodness-of-fit on F^2	1.088	
Final R indices [I>2sigma(I)]	$R1$ = 0.0445, $wR2$ = 0.1043	
R indices (all data)	$R1$ = 0.0566, $wR2$ = 0.1115	
Largest diff. peak and hole	0.201 and -0.243 e.$Å^{-3}$	
CCDC number	2003243	

Table 3. Crystal data and structure refinement for
(*E*)-*N*,*N*-Dimethyl-2-(1-phenylprop-1-en-1-yl)-1-(trifluoromethyl)-1*H*-inden-1-amine (**12d**)

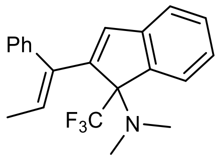

Crystallization from methanol.
Special details: The acentric unit cell ($P2_1$) contains both the (*S*) and the (*R*) enantiomer.

Identification code	ts332	
Empirical formula	C21 H20 F3 N	
Formula weight	343.38	
Temperature	150(2) K	
Wavelength	1.54184 Å	
Crystal system	monoclinic	
Space group	$P2_1$	
Unit cell dimensions	*a* = 14.4968(7) Å	α = 90°.
	b = 7.1146(4) Å	β = 102.817(5)°.
	c = 17.5030(11) Å	γ = 90°.
Volume	1760.26(18) Å^3	
Z	4	
Density (calculated)	1.296 Mg/m^3	
Absorption coefficient	0.811 mm^{-1}	
F(000)	720	
Crystal size	0.21 x 0.16 x 0.07 mm^3	
Theta range for data collection	4.482 to 66.598°.	
Index ranges	-11<=*h*<=17, -8<=*k*<=7, -20<=*l*<=20	
Reflections collected	6431	
Independent reflections	4683 [*R*(int) = 0.0431]	
Completeness to theta = 66.598°	99.8 %	
Absorption correction	Semi-empirical from equivalents	
Max. and min. transmission	1.00000 and 0.82671	
Refinement method	Full-matrix least-squares on F^2	
Data / restraints / parameters	4683 / 1 / 457	
Goodness-of-fit on F^2	1.051	
Final *R* indices [*I*>2sigma(*I*)]	*R*1 = 0.0693, *wR*2 = 0.1890	
R indices (all data)	*R*1 = 0.0798, *wR*2 = 0.1994	
Absolute structure parameter	-0.7(3)	
Extinction coefficient	n/a	
Largest diff. peak and hole	0.562 and -0.245 e.Å^{-3}	
CCDC number	2003241	

Table 4. Crystal data and structure refinement for (*Z*)-2-(4-Bromophenyl)-*N*,*N*-dimethyl-1-phenyl-2-(3-phenyl-2-(trifluoromethyl)cyclopenta-2,4-dien-1-yliden)ethan-1-amine (**19**)

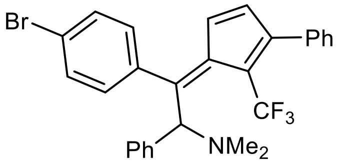

Crystallization from *n*-hexane/ethyl acetate by a vapor diffusion method.

Identification code	BS384	
Empirical formula	C28 H23 Br F3 N	
Formula weight	510.38	
Temperature	150(2) K	
Wavelength	0.71073 Å	
Crystal system	monoclinic	
Space group	$P2_1/n$	
Unit cell dimensions	a = 11.592(4) Å	α = 90°.
	b = 16.124(7) Å	β = 108.008(13)°.
	c = 13.149(5) Å	γ = 90°.
Volume	2337.2(16) Å^3	
Z	4	
Density (calculated)	1.450 Mg/m^3	
Absorption coefficient	1.799 mm^{-1}	
F(000)	1040	
Crystal size	0.259 x 0.212 x 0.144 mm^3	
Theta range for data collection	2.051 to 32.450°.	
Index ranges	-17<=h<=17, -24<=k<=24, -19<=l<=19	
Reflections collected	60248	
Independent reflections	8360 [R(int) = 0.0654]	
Completeness to theta = 25.242°	99.8 %	
Refinement method	Full-matrix least-squares on F^2	
Data / restraints / parameters	8360 / 0 / 300	
Goodness-of-fit on F^2	1.014	
Final R indices [I>2sigma(I)]	R1 = 0.0436, wR2 = 0.0928	
R indices (all data)	R1 = 0.0719, wR2 = 0.1035	
Extinction coefficient	n/a	
Largest diff. peak and hole	0.485 and -0.478 e.Å^{-3}	
CCDC number	2003246	
